# Initial therapeutic anticoagulation with rivaroxaban compared to prophylactic therapy with heparins in moderate to severe COVID-19: results of the COVID-PREVENT randomized controlled trial

**DOI:** 10.1007/s00392-023-02240-1

**Published:** 2023-07-05

**Authors:** Ursula Rauch-Kröhnert, Marianna Puccini, Marius Placzek, Jan Beyer-Westendorf, Kai Jakobs, Julian Friebel, Selina Hein, Mirko Seidel, Burkert Pieske, Steffen Massberg, Martin Witzenrath, Andreas Zeiher, Tim Friede, Stefan D. Anker, Ulf Landmesser

**Affiliations:** 1 Department of Cardiology, Angiology and Intensive Care Medicine, Deutsches Herzzentrum der Charité, Charite Universitätsmedizin Berlin, Campus Benjamin Franklin, Deutsches Herzzentrum der Charité, Hindenburgdamm 30, 12203 Berlin, Germany; 2 DZHK (German Centre for Cardiovascular Research), Partner Site Berlin, Berlin, Germany; 3 Friede Springer Cardiovascular Prevention Center @ Charite Universitätsmedizin Berlin, Berlin, Germany; 4 DZHK (German Centre for Cardiovascular Research), Partner Site Göttingen, Göttingen, Germany; 5 Department of Medical Statistics, University Medical Center Göttingen, Göttingen, Germany; 6 Department of Medicine I, Universitätsklinikum “Carl Gustav Carus” Dresden, Dresden, Germany; 7 Berlin Institute of Health at Charité-Universitätsmedizin Berlin, Berlin, Germany; 8 Katholisches Klinikum Koblenz-Montabaur, Koblenz, Germany; 9 BG Klinikum Unfallkrankenhaus Berlin, Berlin, Germany; 10 German Heart Center Berlin, Berlin, Germany; 11 Department of Cardiology Campus Virchow Klinikum, Charité-Universitätsmedizin Berlin, Berlin, Germany; 12 Department of Medicine I, LMU Klinikum, Ludwig-Maximilians-Universität München, Munich, Germany; 13 DZHK (German Centre for Cardiovascular Research), Partner Site Munich, Munich, Germany; 14 Department of Infectious Diseases and Respiratory Medicine, Charité-Universitätsmedizin Berlin, Berlin, Germany; 15 Division of Cardiology, Department of Medicine III, University Hospital Frankfurt, Goethe University Frankfurt am Main, Frankfurt, Germany; 16 DZHK (German Centre for Cardiovascular Research), Partner Site Rhine-Main, Frankfurt, Germany; 17 Department of Cardiology, Angiology and Intensive Care Medicine, Deutsches Herzzentrum der Charité, Campus Virchow Klinikum, Berlin, Germany; 18 Berlin Institute of Health Center for Regenerative Therapies (BCRT), Berlin, Germany

**Keywords:** COVID-19, Rivaroxaban, Anticoagulation, Thromboprophylaxis

## Abstract

**Background:**

COVID-19 is associated with a prothrombotic state. Current guidelines recommend prophylactic anticoagulation upon hospitalization.

**Methods:**

COVID-PREVENT, an open-label, multicenter, randomized, clinical trial enrolled patients (≥ 18 years) with moderate to severe COVID-19 and age-adjusted d-dimers > 1.5 upper limit of normal (ULN). The participants were randomly assigned (1:1) to receive either therapeutic anticoagulation with rivaroxaban 20 mg once daily or thromboprophylaxis with a heparin (SOC) for at least 7 days followed by prophylactic anticoagulation with rivaroxaban 10 mg once daily for 28 days or no thromboprophylaxis. The primary efficacy outcome was the d-dimer level and the co-primary efficacy outcome the 7-category ordinal COVID-19 scale by WHO at 7 days post randomization. The secondary outcome was time to the composite event of either venous or arterial thromboembolism, new myocardial infarction, non-hemorrhagic stroke, all-cause death or progression to intubation and invasive ventilation up to 35 days post randomization.

**Results:**

The primary efficacy outcome d-dimer at 7 days was not different between patients assigned to therapeutic (n = 55) or prophylactic anticoagulation (n = 56) (1.21 mg/L [0.79, 1.86] vs 1.27 mg/L [0.79, 2.04], p = 0.78). In the whole study population d-dimer was significantly lower at 7 days compared to baseline (1.05 mg/L [0.75, 1.48] vs 1.57 mg/L [1.13, 2.19], p < 0.0001). Therapy with rivaroxaban compared to SOC was not associated an improvement on the WHO 7-category ordinal scale at 7 days (p = 0.085). Rivaroxaban improved the clinical outcome measured by the score in patients with a higher baseline d-dimer  > 2.0 ULN (exploratory analysis; 0.632 [0.516, 0.748], p = 0.026). The secondary endpoint occurred in 6 patients (10.9%) in the rivaroxaban group and in 12 (21.4%) in the SOC group (time-to-first occurrence of the components of the secondary outcome: HR 0.5; 95% CI 0.15–1.67; p = 0.264). There was no difference in fatal or non-fatal major or clinically relevant non-major bleeding between the groups.

**Conclusions:**

Therapeutic anticoagulation with rivaroxaban compared to prophylactic anticoagulation with a heparin did not improve surrogates of clinical outcome in patients with moderate to severe COVID-19. Whether initial rivaroxaban at therapeutic doses might be superior to thromboprophylaxis in patients with COVID-19 and a high risk as defined by d-dimer  > 2 ULN needs confirmation in further studies.

**Graphical abstract:**

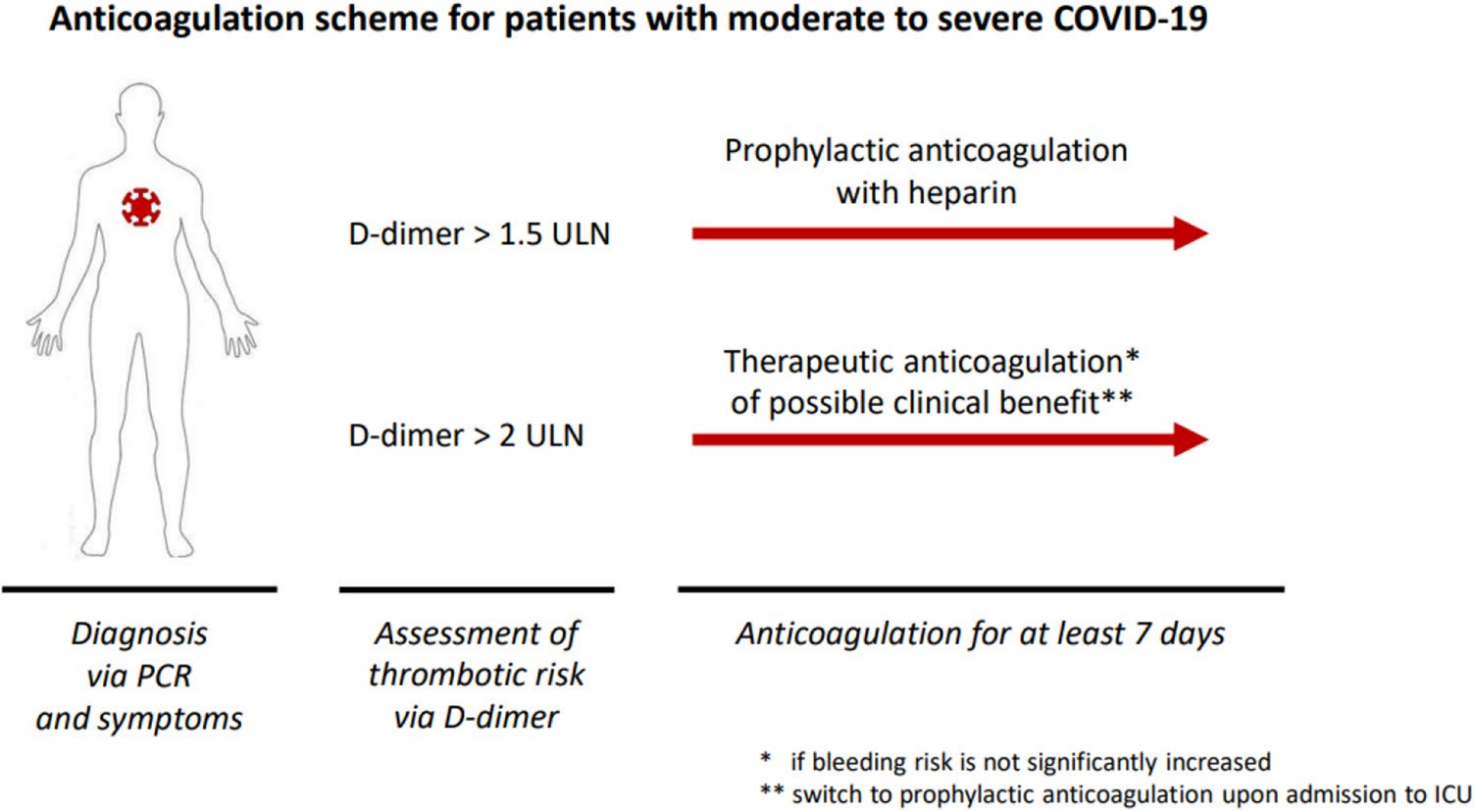

**Supplementary Information:**

The online version contains supplementary material available at 10.1007/s00392-023-02240-1.

## Introduction

Patients with COVID-19 exhibit a higher risk of thrombotic events and a greater magnitude of disease severity than those with respiratory infections of other causes [1, 2]. A heightened inflammatory reaction with increased cytokine and chemokine secretion has been associated with adverse clinical outcomes in moderate to severe COVID-19 [3]. Immunothrombosis characterized by not only severe inflammation but also endothelial dysfunction, platelet hyperreactivity and coagulation activation, increases the risk of thrombosis in the micro- and macrovascular bed [4, 5]. These thrombotic events affect the micro- and marcrovasculature of the lung leading to respiratory symptoms up to severe acute respiratory distress syndrome [6]. Venous and arterial thromboembolic events such as deep venous thrombosis and pulmonary embolism, stroke and myocardial infarction occur more frequently in patients with COVID-19 [7].d-Dimer is a clinical marker used to identify patients with a high risk for thrombotic complications [8, 9]. Several studies have investigated whether COVID-19 patients with increased d-dimer need more intense antithrombotic therapy for the prevention of thromboembolic events [10]. The first studies that compared therapeutic and prophylactic anticoagulation with no anticoagulation showed a reduction in thrombotic events in the anticoagulation group, contributing to reduced mortality and intubation rates [11, 12]. ESC recommendations for antithrombotic therapy in patients hospitalized with moderate to severe COVID-19 suggested prophylactic anticoagulation with heparins only at the time of initiation [13, 14]. At that time COVID-PREVENT several studies had examined the effect of therapeutic anticoagulation with heparins on the clinical outcome of patients with moderate to severe COVID-19, the latter treated on the intensive care unit [15, 16]. Therapeutic anticoagulation was associated with increased major bleeding events of those patients on ICU. Regarding the higher incidence of bleeding in patients with COVID-19 and critical illness it was not recommended to apply therapeutic anticoagulation in this patient population at the time of the start of COVID-PREVENT [16].

Non-vitamin K dependent oral inhibitors of factor Xa (NOAC) such as rivaroxaban are widely administered for thromboprophylaxis due to their effectivity to prevent thrombotic events and safety profile [10, 17]. At the time of COVID-PREVENT no clinical trials that utilized NOAC for anticoagulation in patients with COVID-19 had been conducted. Therefore, COVID-PREVENT was designed not only to test the effect of therapeutic versus prophylactic anticoagulation but also the applicability of a NOAC as an anticoagulation in these patients. Moreover, alterations in d-dimer levels might indicate in a more sensitive way the effect of anticoagulation on the coagulation system than monitoring the occurrence of thrombotic events only. The 7-category ordinal scale by the WHO to assess the clinical outcome of patients with pulmonary diseases is a sensitive measure for evaluating treatment effects in these patients. Therefore, we conducted a randomized controlled trial in patients with moderate to severe COVID-19 and increased d-dimer that compared the effect of therapeutic anticoagulation with rivaroxaban to prophylactic anticoagulation with heparins on d-dimer and a 7-category ordinal scale by WHO as measures for the clinical outcome.

## Methods

### Study design

The COVID-PREVENT trial was an open-label, multicenter, randomized, controlled clinical trial in patients with moderate to severe COVID-19 disease and a high prothrombotic risk defined by an elevated d-dimer concentration. The study included patients at 14 trial sites in Germany. The main objective of the trial was to assess the antithrombotic effectiveness after 7 days of therapeutic anticoagulation with rivaroxaban in comparison with prophylactic anticoagulation with a heparin (unfractionated heparin or low molecular weight heparin) at prophylactic doses as standard of care (SOC). The decreasing incidence of COVID-19 in Germany in March–April 2021 unfortunately made it impossible to recruit the initially planned number of more than 400 patients (Supplementary Appendix 1) in a timely manner. The statistical power to test our primary hypotheses was not reached due to the number of patients enrolled into the study that was smaller than initially planned and expected. The study protocol including the efficacy outcomes therefore had to be adapted. The amendment with those adaptations including primary and secondary efficacy outcomes (Supplementary Appendix 1) was approved by the regulatory authorities. The primary efficacy endpoint was now the d-dimer concentration as a clinical prognosis marker at day 7 post randomization and the co-primary efficacy endpoint the clinical status measured by seven-category ordinal scale by WHO R&D Blueprint expert group used for respiratory infections [18, 19] at day 7 post randomization. The secondary efficacy outcome was a composite of venous thromboembolism, arterial thromboembolism, new myocardial infarction, non-hemorrhagic stroke, all-cause death or progression to intubation and invasive ventilation until day 35 post randomization. The primary safety outcome was fatal or non-fatal major bleeding—defined according to the International Society on Thrombosis and haemostasis (ISTH) criteria—until day 35 post randomization. The secondary safety outcome was non-major clinical relevant bleeding according to the ISTH criteria [20]. A blinded, independent clinical event committee adjudicated the secondary efficacy outcomes and the safety outcomes.

The study protocol was approved according to local regulatory requirements by the ethics committee and the Federal Institute for Drugs and Medical Devices. The study protocol inclusive its amendments and adaptions were registered at clinicaltrials.gov (NCT044160048). An independent data safety monitoring board was assigned to monitor the trial for efficacy and safety. Due to the decreasing incidence of COVID-19 in Germany during the third wave in spring 2021 and the likelihood not to be able to recruit the initially planned number of patients in a timely manner, the steering committee decided to stop the recruitment in June 2021. The approved protocol version with the summary of changes in protocol version 2.0 are provided in the Supplementary Appendix 1.

### Study population

Ambulatory or hospitalized adults (≥ 18 years old) with moderate to severe COVID-19 confirmed by a positive polymerase chain reaction (PCR) for up to 14 days prior to randomization were eligible for the study. Moderate cases were defined as patients with fever and/or respiratory symptoms and one of the following signs of pulmonary distress: respiratory rate > 22/min, oxygen saturation ≤ 95% or radiological findings of pneumonia. Severe cases were patients that met any of the following criteria: respiratory rate ≥ 30/min, oxygen saturation ≤ 93% at rest or a PaO_2_/FiO_2_ ratio ≤ 300 mgHg [21]. Additionally, the patients had an elevated d-dimer concentration (1.5 times above upper limit of normal with age adjusted cut-offs for patients over 50 years, reference range according to local laboratory) and/or a troponin T elevated 2 times the upper limit of normal plus one of the following conditions: known coronary artery disease, known diabetes mellitus or active smoking.

Patients with a high bleeding risk, platelet count < 90.000/µL, active cancer, any medical condition that required an anticoagulation, a contraindication for the use of rivaroxaban or heparin, an estimated glomerular filtration rate (eGRF) less than 30 mL/min/1.73 m^2^ or patients on dual antiplatelet therapy were excluded from the study as well as critically ill patients with immediate indication for an intensive care unit admission. Patients who had already received thromboprophylaxis as part of the COVID-19 treatment could be included in the study. Full eligibility criteria can be found on Supplementary Appendix 1.

### Randomization and study interventions

After written informed consent was obtained from each patient, patients were randomly assigned in a 1:1 ratio to receive either standard of care with unfractionated heparin or low molecular weight heparin at a prophylactic dose or therapeutic anticoagulation with rivaroxaban during hospitalization followed by a four-week period of thromboprophylaxis with rivaroxaban. The randomization was done using a central, web-based system and was stratified by: site, gender, age (< 65 versus ≥ 65 years), kidney function (eGFR ≥ 30 mL/min/1.73 m^2^ and < 50 mL/min/1.73 m^2^ versus eGFR ≥ 50 mL/min/1.73 m^2^), history of CAD or heart failure (yes/no), oxygen demand on admission to the hospital (supplementary oxygen required versus not required) and setting (outpatients versus hospitalized patients).

**Fig. 1:**
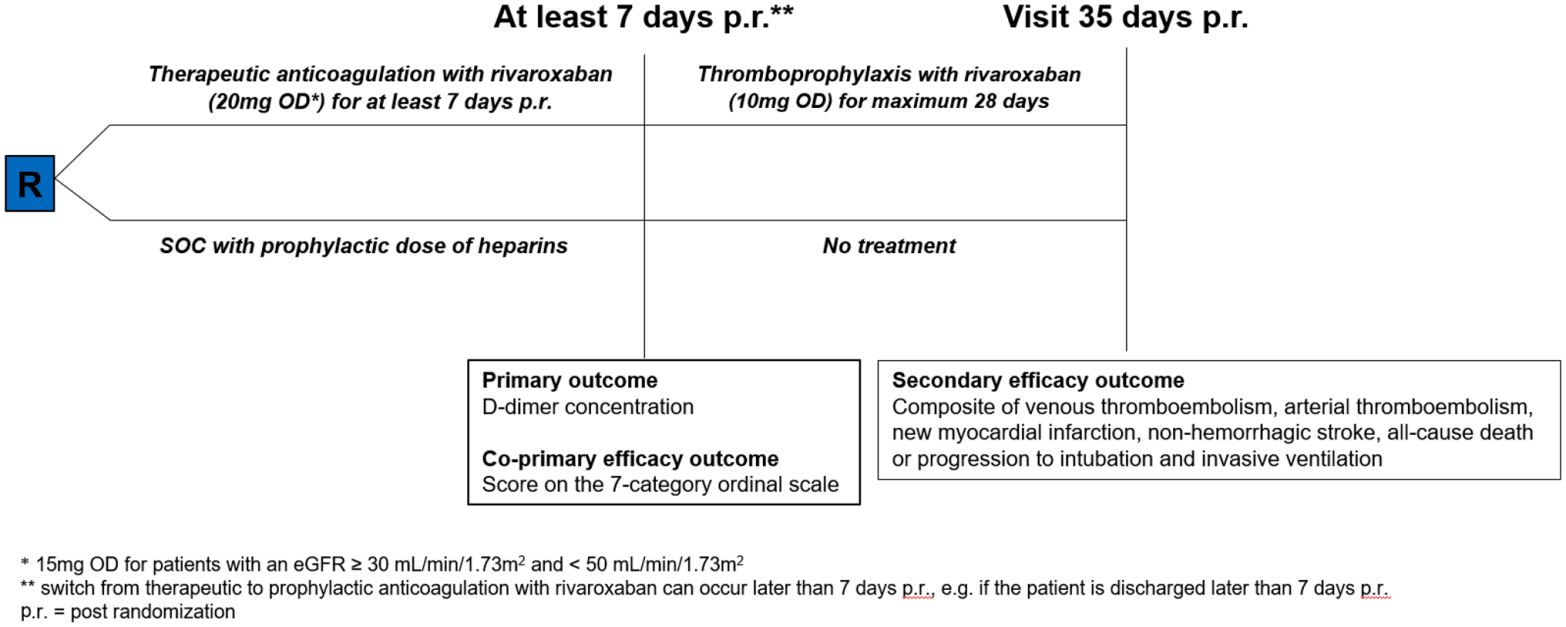
Study flow and efficacy outcomes

The patients that were assigned to the standard of care group received unfractionated heparin or a low molecular weight heparin at a prophylactic dose for at least 7 days or until discharge (if hospitalized), whichever occurred later. The patients on the intervention arm received rivaroxaban 20 mg once daily (adjusted to 15 mg once daily for patients with an eGFR < 50 mL/min/1.73 m^2^) for at least 7 days or until discharge (whichever occurred later) followed by a four-week period with a lower dose of rivaroxaban of 10 mg once daily without adjusting to kidney function. Upon admission of the patients to the ICU it was recommended to switch from therapeutic anticoagulation with rivaroxaban to prophylactic anticoagulation with a heparin (Fig. 1). This clinical care was provided at discretion by the treating physician based on the local standard of care and current guidelines.

The assessment at baseline included demographic characteristics, risk factors, medical history, and laboratory data. Follow-up was done at day 7 and 35 post randomization to assess study outcomes. Additional safety information was collected by telephone at day 60 post randomization.

### Statistical analysis

Sample size calculation was based on the primary endpoint d-dimer at day 7 corrected for baseline values. Since the distribution of d-dimer concentrations is skewed, calculations were done on the log scale. Based on a blinded review, a common standard deviation of SD = 0.8 and a correlation to baseline of r = 0.5 (both on the natural log scale) were assumed. The effect assumed translates to a reduction of 36% in d-dimer values on the original scale. Using an analysis of covariance (ANCOVA) (significance level alpha = 0.05, two-sided) with baseline values as covariate a total sample size of 80, i.e. 40 per group, gives a power of 80% to detect a mean difference of 0.44 (on the log scale) between the treatment group and the control group at day 7.

The primary efficacy endpoint, d-dimer at day 7 post randomization, adjusted for baseline measurement was analyzed transformed to a logarithmic scale by an analysis of covariance (ANCOVA). The model included treatment group and stratification variables of the randomization (including site, gender (male, female, diverse), age (< 65 versus ≥ 65 years), kidney function (eGFR ≥ 30 mL/min/1.73 m^2^ and < 50 mL/min/1.73 m^2^ versus eGFR ≥ 50 mL/min/1.73 m^2^), history of CAD or heart failure (yes/no), oxygen demand on admission to the hospital [supplementary oxygen required versus not required), setting (outpatients versus hospitalized patients)] as factors and the logarithmic baseline d-dimer measurement as covariate. Least squares means for d-dimer at day 7 in the two treatment groups were calculated with 95% confidence intervals as well as the difference between the treatment groups at day 7 with 95% confidence interval and p-value testing the null hypothesis of no treatment difference, i.e. a mean difference (on the logarithmic scale) of 0 (significance level alpha = 0.05, two-sided). For ease of interpretation the results were converted back to the original measurement scale. A longitudinal effect in d-dimer values over time was analyzed using a mixed model for repeated measures (MMRM) with baseline values and values at day 7 as longitudinal measurements (on the logarithmic scale). The model included treatment, visit, treatment-by-visit interaction and stratification variables of the randomization as factors.

The co-primary endpoint, the seven-category ordinal scale recommended by the WHO at day 7 post randomization, adjusted for baseline score was analyzed using a nonparametric Wilcoxon (Mann–Whitney) rank-sum test stratified by baseline score dichotomized as smaller or equal 3 vs. larger 3. The treatment effect was reported as relative effect, i.e. the probability that an observation in the treatment group is smaller (more favorable) than an observation in the control group, with a 95% confidence interval.

The secondary composite endpoint, time to first event of either venous thromboembolism, arterial thromboembolism, new myocardial infarction, non-hemorrhagic stroke, all-cause death or progression to intubation and invasive ventilation until day 35 post randomization, was analyzed using a Cox proportional hazards regression model with treatment group as factor. Due to the small number of events it was not adjusted for stratification factors of the randomization. Reported were a hazard ratio (HR) with 95% confidence interval and a p-value for the null hypothesis H0: HR = 1. The secondary composite endpoint was visualized as Kaplan–Meier curves.

Analyses were performed using the intention-to-treat population (IIT population). Patients that were erroneously randomized with a wrong stratum were analyzed as if they were randomized correctly, i.e. in their true stratum according to their baseline characteristics.

Additionally, as explorative analysis the primary and co-primary endpoint were analyzed in a subgroup of patients with a very high risk as defined by d-dimer  > 1 mg/L at baseline.

The statistical analysis and the graphics were generated with the software R, Version 4.1.3.

## Results

### Patient characteristics

117 patients were screened between November 30, 2020 and May 28, 2021 in 14 trial sites across Germany. 111 patients were randomized; 55 (50%) to the rivaroxaban-group and 56 (50%) to the group with standard of care (SOC) group, in which a heparin was administered at a prophylactic dose. Baseline characteristics were similar between groups (Tables 1, 2). The mean age in the total cohort was 61.2 years (SD 15.827). 61.3% of the total population were male and the mean BMI was 29.03 kg/m^2^. Only one patient in the SOC group was not hospitalized at the time of inclusion, the rest of the subjects were admitted to the hospital. 21.6% of the overall population needed oxygen supply at the time of hospital admission. 42 patients (37.8%) of the overall population were taking aspirin at the time of randomization and the mean d-dimer levels in the overall population was 2.287 mg/L (SD 3.898; 2.119 in the rivaroxaban group vs 2.451 ng/mL in the SOC group) (Table 2).

**Table 1:** Baseline characteristics

	Rivaroxaban (N = 55)	SOC (N = 56)	Total (N = 111)
Age at inclusion (years)			
Mean (SD)	61.055 (14.298)	61.518 (17.325)	61.288 (15.827)
Sex			
Female	21 (38.2%)	22 (39.3%)	43 (38.7%)
Male	34 (61.8%)	34 (60.7%)	68 (61.3%)
Weight (kg)			
Mean (SD)	86.455 (18.187)	86.855 (17.616)	86.651 (17.826)
BMI (kg/m^2^)			
Mean (SD)	29.039 (5.332)	29.027 (5.264)	29.033 (5.274)
Hospitalization on inclusion			
No	0 (0.0%)	1 (1.8%)	1 (0.9%)
Yes	55 (100.0%)	55 (98.2%)	110 (99.1%)
Asthma			
Yes	3 (5.5%)	2 (3.6%)	5 (4.5%)
COPD			
Yes	2 (3.6%)	5 (8.9%)	7 (6.3%)
Diabetes mellitus			
Yes	12 (21.8%)	8 (14.3%)	20 (18.0%)
Chronic heart failure			
Yes	3 (5.5%)	0 (0.0%)	3 (2.7%)
Coronary artery disease			
Yes	5 (9.1%)	3 (5.4%)	8 (7.2%)
Hypertension			
Yes	24 (43.6%)	30 (53.6%)	54 (48.6%)
Ischemic stroke			
Yes	0 (0.0%)	2 (3.6%)	2 (1.8%)
Current smoker			
No	47 (85.5%)	51 (91.1%)	98 (88.3%)
Unknown	4 (7.3%)	3 (5.4%)	7 (6.3%)
Yes	4 (7.3%)	2 (3.6%)	6 (5.4%)
Ever smoked?			
No	26 (47.3%)	27 (48.2%)	53 (47.7%)
Unknown	4 (7.3%)	4 (7.1%)	8 (7.2%)
Yes	21 (38.2%)	23 (41.1%)	44 (39.6%)
Time from hosp to rand (days)			
Mean (SD)	3.382 (3.818)	4.855 (5.289)	4.118 (4.651)
Oxygen demand on admission to the hospital?			
No	11 (20.0%)	13 (23.2%)	24 (21.6%)
Yes	44 (80.0%)	43 (76.8%)	87 (78.4%)
Ventilation			
No	55 (100.0%)	53 (94.6%)	108 (97.3%)
Yes	0 (0.0%)	3 (5.4%)	3 (2.7%)
Oxygen supply?			
No	7 (12.7%)	9 (16.1%)	16 (14.4%)
Yes	48 (87.3%)	44 (78.6%)	92 (82.9%)
Oxygen application			
Mask with reservoir	0 (0.0%)	2 (3.6%)	2 (1.8%)
Mask without reservoir	3 (5.5%)	2 (3.6%)	5 (4.5%)
Nasal cannula	45 (81.8%)	40 (71.4%)	85 (76.6%)
Creatinine (mg/dL)			
Mean (SD)	0.924 (0.291)	0.866 (0.231)	0.895 (0.263)

**Table 2:** Baseline medication and laboratory parameters

Anticoagulation before randomization			
FALSE	23 (41.8%)	22 (39.3%)	45 (40.5%)
TRUE	32 (58.2%)	34 (60.7%)	66 (59.5%)
ASS			
Yes	18 (32.7%)	24 (42.9%)	42 (37.8%)
ACE inhibitors			
Yes	15 (27.3%)	15 (26.8%)	30 (27.0%)
Aldosterone antagonists			
Yes	3 (5.5%)	0 (0.0%)	3 (2.7%)
Angiotensin II receptor blockers (ARBs)			
Yes	10 (18.2%)	13 (23.2%)	23 (20.7%)
Antibiotics			
Yes	23 (41.8%)	23 (41.1%)	46 (41.4%)
Antibodies/Immunomodulators			
Yes	2 (3.6%)	2 (3.6%)	4 (3.6%)
Antivirals			
Yes	4 (7.3%)	6 (10.7%)	10 (9.0%)
Beta blocking agents			
Yes	14 (25.5%)	17 (30.4%)	31 (27.9%)
Clopidogrel			
Yes	1 (1.8%)	1 (1.8%)	2 (1.8%)
Diuretics			
Yes	15 (27.3%)	13 (23.2%)	28 (25.2%)
Glucocorticoids			
Yes	37 (67.3%)	36 (64.3%)	73 (65.8%)
HMG CoA reductase inhibitors			
Yes	7 (12.7%)	8 (14.3%)	15 (13.5%)
NSAIDs			
Yes	4 (7.3%)	3 (5.4%)	7 (6.3%)
Proton pump inhibitors			
Yes	28 (50.9%)	29 (51.8%)	57 (51.4%)
d-Dimer (mg/L)			
N-Miss	1	1	2
Mean (SD)	2.119 (3.024)	2.451 (4.622)	2.287 (3.898)
Range	0.800–21.792	0.845–35.020	0.800–35.020
d-Dimer > 2 ULN			
No	11 (20.4%)	10 (18.2%)	21 (19.3%)
Yes	43 (79.6%)	45 (81.8%)	88 (80.7%)
Creatinine clearance (mL/min)			
Mean (SD)	106.561 (42.550)	110.597 (51.584)	108.541 (47.021)
> 0.5 ULN (+ age dep. adapt)			
N-Miss	1	1	2
FALSE	0 (0.0%)	0 (0.0%)	0 (0.0%)
TRUE	54 (100.0%)	55 (100.0%)	109 (100.0%)

The baseline characteristics in the subgroup of patients with a d-dimer  > 1 mg/L are show in Tables 3 and 4. The baseline characteristics were similar to those in the overall cohort. The mean d-dimer in this subgroup was 2.620 mg/L (SD 4.275).

**Table 3:** Baseline characteristics (d-dimer  > 2ULN)

	Rivaroxaban (N = 43)	SOC (N = 45)	Total (N = 88)
Age at inclusion (years)			
Mean (SD)	63.000 (14.248)	63.911 (16.662)	63.466 (15.447)
Sex			
Female	18 (41.9%)	16 (35.6%)	34 (38.6%)
Male	25 (58.1%)	29 (64.4%)	54 (61.4%)
Weight (kg)			
Mean (SD)	85.233 (17.514)	85.342 (16.831)	85.287 (17.075)
BMI (kg/m^2^)			
Mean (SD)	28.763 (4.913)	27.972 (4.120)	28.367 (4.524)
Hospitalization on inclusion			
No	0 (0.0%)	1 (2.2%)	1 (1.1%)
Yes	43 (100.0%)	44 (97.8%)	87 (98.9%)
Asthma			
Yes	3 (7.0%)	1 (2.2%)	4 (4.5%)
COPD			
Yes	2 (4.7%)	3 (6.7%)	5 (5.7%)
Diabetes mellitus			
Yes	9 (20.9%)	6 (13.3%)	15 (17.0%)
Chronic heart failure			
Yes	3 (7.0%)	0 (0.0%)	3 (3.4%)
Coronary artery disease			
Yes	5 (11.6%)	2 (4.4%)	7 (8.0%)
Hypertension			
Yes	21 (48.8%)	25 (55.6%)	46 (52.3%)
Ischemic stroke			
Yes	0 (0.0%)	2 (4.4%)	2 (2.3%)
Current smoker			
No	38 (88.4%)	40 (88.9%)	78 (88.6%)
Unknown	3 (7.0%)	3 (6.7%)	6 (6.8%)
Yes	2 (4.7%)	2 (4.4%)	4 (4.5%)
Ever smoked?			
No	20 (46.5%)	21 (46.7%)	41 (46.6%)
Unknown	3 (7.0%)	4 (8.9%)	7 (8.0%)
Yes	18 (41.9%)	18 (40.0%)	36 (40.9%)
Time from hospitalization to randomization (days)			
Mean (SD)	3.814 (4.176)	5.114 (5.772)	4.471 (5.060)
Oxygen demand on admission to the hospital?			
No	9 (20.9%)	9 (20.0%)	18 (20.5%)
Yes	34 (79.1%)	36 (80.0%)	70 (79.5%)
Ventilation			
No	43 (100.0%)	42 (93.3%)	85 (96.6%)
Yes	0 (0.0%)	3 (6.7%)	3 (3.4%)
Oxygen supply?			
No	5 (11.6%)	6 (13.3%)	11 (12.5%)
Yes	38 (88.4%)	36 (80.0%)	74 (84.1%)
Oxygen application			
Mask with reservoir	0 (0.0%)	2 (4.4%)	2 (2.3%)
Mask without reservoir	2 (4.7%)	2 (4.4%)	4 (4.5%)
Nasal cannula	36 (83.7%)	32 (71.1%)	68 (77.3%)
Creatinine (mg/dL)			
Mean (SD)	0.914 (0.290)	0.889 (0.237)	0.901 (0.263)

**Table 4:** Baseline medication and laboratory parameters (d-dimer  > 2ULN)

Anticoagulation before randomization			
FALSE	17 (39.5%)	17 (37.8%)	34 (38.6%)
TRUE	26 (60.5%)	28 (62.2%)	54 (61.4%)
ASS			
Yes	17 (39.5%)	21 (46.7%)	38 (43.2%)
ACE inhibitors			
Yes	13 (30.2%)	10 (22.2%)	23 (26.1%)
Aldosterone antagonists			
Yes	3 (7.0%)	0 (0.0%)	3 (3.4%)
Angiotensin II receptor blockers (ARBs)			
Yes	8 (18.6%)	12 (26.7%)	20 (22.7%)
Antibiotics			
Yes	21 (48.8%)	19 (42.2%)	40 (45.5%)
Antibodies/immunomodulators			
Yes	2 (4.7%)	2 (4.4%)	4 (4.5%)
Antivirals			
Yes	4 (9.3%)	5 (11.1%)	9 (10.2%)
Beta blocking agents			
Yes	11 (25.6%)	14 (31.1%)	25 (28.4%)
Clopidogrel			
Yes	1 (2.3%)	0 (0.0%)	1 (1.1%)
Diuretics			
Yes	13 (30.2%)	12 (26.7%)	25 (28.4%)
Glucocorticoids			
Yes	28 (65.1%)	27 (60.0%)	55 (62.5%)
HMG CoA reductase inhibitors			
Yes	5 (11.6%)	8 (17.8%)	13 (14.8%)
NSAIDs			
Yes	3 (7.0%)	2 (4.4%)	5 (5.7%)
Progestogens			
Proton pump inhibitors			
Yes	21 (48.8%)	23 (51.1%)	44 (50.0%)
Tamoxifen			
No	43 (100.0%)	45 (100.0%)	88 (100.0%)
Testosterone			
No	43 (100.0%)	45 (100.0%)	88 (100.0%)
d-dimer (mg/L)			
Mean (SD)	2.439 (3.320)	2.792 (5.055)	2.620 (4.275)
Range	1.001–21.792	1.010–35.020	1.001–35.020
Creatinine clearance (mL/min)			
Mean (SD)	103.736 (43.458)	104.941 (49.716)	104.339 (46.420)

### Efficacy outcomes

The primary efficacy outcome d-dimer at day 7 was not different between the groups (1.21 mg/L [0.79, 1.86] in the rivaroxaban-group vs 1.27 mg/L [0.79, 2.04] in the SOC-group, p = 0.78). In the whole study population d-dimer was significantly reduced at 7d compared to baseline (1.05 mg/L [0.75, 1.48] vs 1.57 mg/L [1.13, 2.19], p < 0.0001).

The subgroup of patients with d-dimer  > 1 mg/L at admission was separately considered in an explorative analysis. No significant difference in d-dimer at day 7 was also observed between the two treatment groups (1.12 mg/L [0.68, 1.82] in the rivaroxaban-group vs. 1.16 mg/L [0.68, 1.99] in the SOC-group, p = 0.855) (Table 5).

**Table 5:** Efficacy outcomes

Primary efficacy outcome				
d-Dimer (mg/L)	Baseline		Day 7 p.r.	
	N	mean [95% CI]	N	marg. mean [95% CI]
Rivaroxaban	54	1.59 [1.35,1.86]	41	1.21 [0.79,1.86]
SOC	55	1.69 [1.42,2.01]	41	1.27 [0.79,2.04]
Treatment effect (log-scale) [log(mg/L)]				− 0.05 [− 0.38,0.29]
p-Value (ANCOVA for day 7)				0.78
Subgroup (d-dimer > 1 mg/L)				
Rivaroxaban	43	1.85 [1.55,2.21]	33	1.12 [0.68,1.82]
SOC	45	1.94 [1.6,2.34]	33	1.16 [0.68,1.99]
Treatment effect (log-scale) [log(mg/L)]				− 0.04 [− 0.44, 0.36]
p-Value (ANCOVA for day 7)				0.855

Co-primary efficacy outcome														
Seven-category ordinal scale by WHO	N	Score at baseline						N	Score at day 7 p.r					
		1	2	3	4	5	6		1	2	3	4	5	6
Rivaroxaban	55	–	–	5	50	–	–	54	7	19	8	16	3	1
SOC	56	–	1	8	47	–	–	54	6	12	13	14	8	1
Relative effect [95% CI]									0.59 [0.48, 0.70]					
p-Value (Wilcoxon rank-sum test)									0.085					
Subgroup (d-dimer > 1 mg/L)														
Rivaroxaban	43	–	–	2	41	–	–	42	5	16	4	14	3	–
SOC	45	–	1	6	38	–	–	43	3	9	12	12	6	1
Relative effect [95% CI]									0.63 [0.51, 0.75]					
p-Value (Wilcoxon rank-sum test)									0.026					

Regarding the co-primary efficacy outcome, a 7-day course of rivaroxaban at a therapeutic dose of 20 mg daily compared to heparin at a prophylactic dose did not improve the 7-category ordinal scale as a measure for the clinical outcome (relative effect 0.5922 [0.4873, 0.6971], Wilcoxon rank-sum test p = 0.085).

**Fig. 2:**
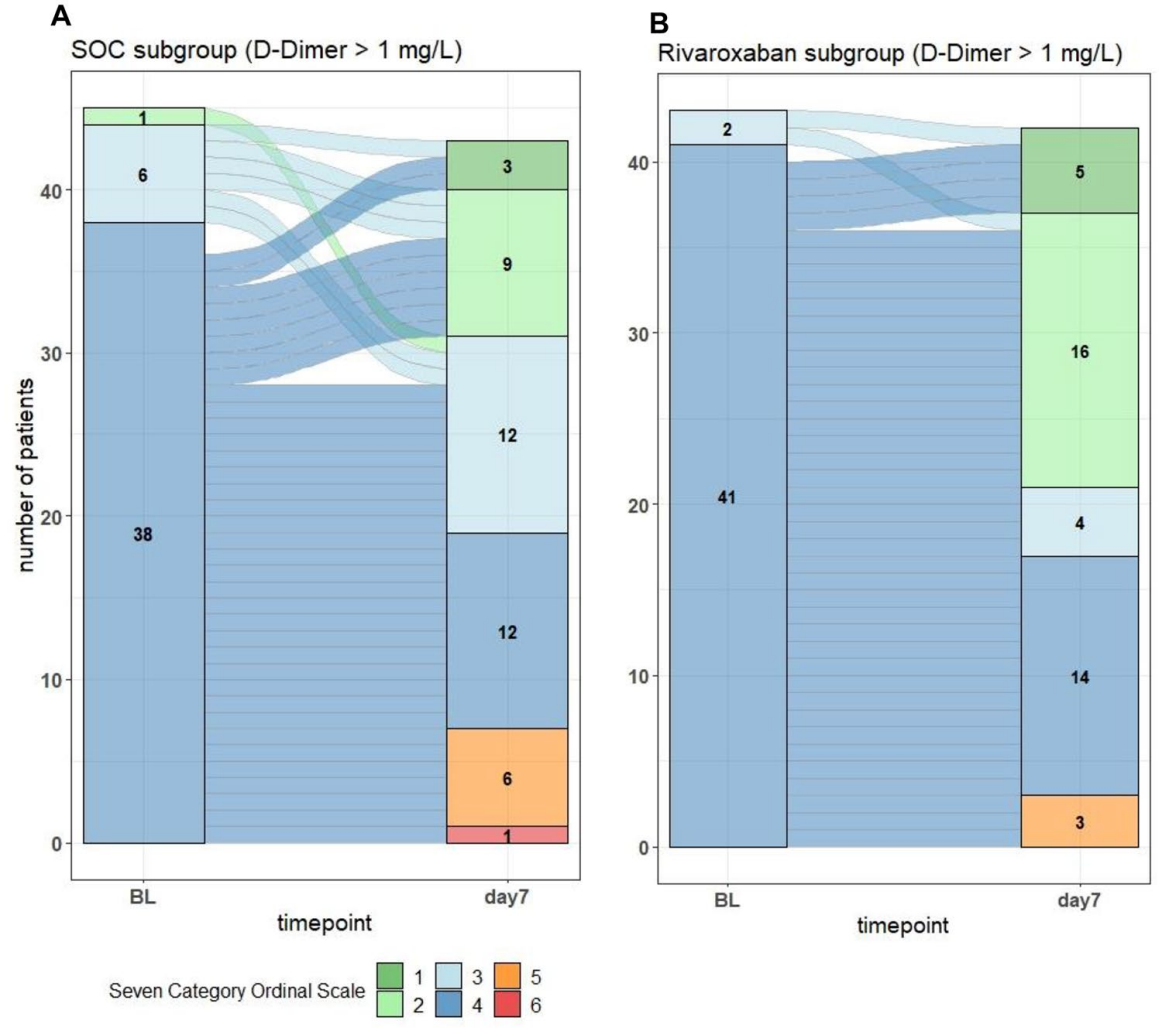
Co-primary efficacy outcome. Seven-category ordinal scale by WHO at baseline (BL) and 7 days post randomization in the subgroup of COVID-19 patients with a d-dimer  > 1 mg/L in the SOC group (**a**) and in the rivaroxaban group (**b**). Wilcoxon rank-sum test, relative effect [95% CI] 0.63 [0.51, 0.75], p = 0.026

In an explorative analysis, therapeutic anticoagulation with rivaroxaban, but not thromboprophylaxis with a heparin led to an improvement on the 7-category ordinal scale by WHO in patients with a very high risk as defined by d-dimer  > 1 mg/L at baseline (relative effect 0.632 [0.516, 0.748], Wilcoxon rank-sum test p = 0.026) (Fig. 2).

**Fig. 3:**
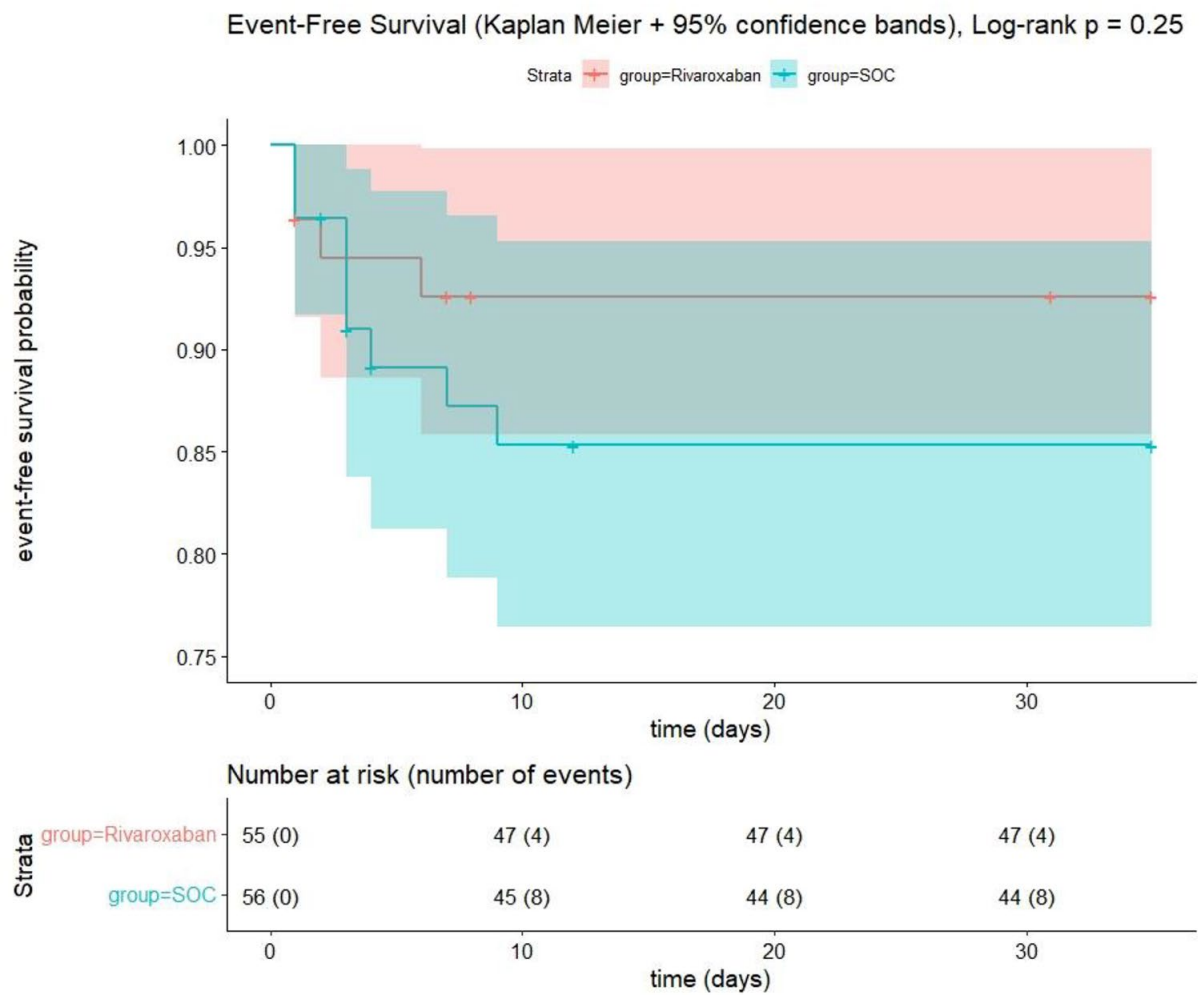
Time to first occurrence of one of the components of the secondary efficacy outcome

The secondary efficacy composite endpoint occurred in 6 patients (10.9%) in the rivaroxaban-group and in 12 patients (21.4%) in the SOC group (time-to-first occurrence of the components of the secondary outcome: HR 0.5; 95% CI 0.15–1.67; p = 0.264) (Fig. 3).

**Fig. 4:**
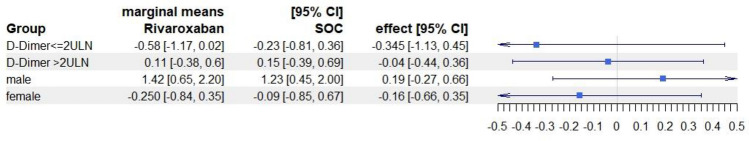
Forest plot of the results for the primary endpoint d-dimer at day 7 (on the log-scale). Shown are the subgroups of high risk patients (d-dimer  > 2ULN), low risk patients, male and female patients

**Fig. 5:**
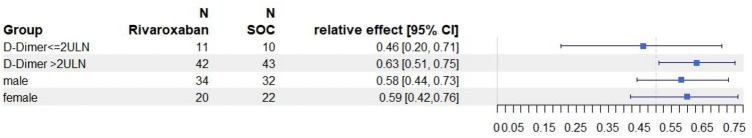
Forest plot of the results for the co-primary endpoint, the seven-category ordinal scale by WHO, at day 7. Shown are the subgroups of high risk patients (d-dimer  > 2ULN), low risk patients, male and female patients

Results for subgroup analyses concerning the primary and co-primary endpoint are shown in Figs. 4 and 5 as forest plots. Subgroups included are the high and low risk groups as defined by d-dimer (> 2 ULN vs. ≤ 2 ULN) as well as the gender subgroups (male vs. female).

### Safety outcomes

The bleeding rate in COVID-PREVENT was overall low. The primary safety outcome occurred in only 1 of 111 patients (0.9%) of the whole study population. The patients randomized to the rivaroxaban group had a non-fatal major bleeding according to the ISTH criteria. The secondary efficacy outcome defined as non-major clinically relevant bleeding occurred in two patients in the rivaroxaban-group and in three patients in the SOC-group (Table 6).

**Table 6:** Secondary outcomes

Secondary efficacy outcome: time to first occurrence of either (composite endpoint)			
	Rivaroxaban	SOC	Total
Venous thromboembolism	1	4	5
Arterial thromboembolism	0	1	1
New myocardial infarction	0	2	2
Non-hemorrhagic stroke	0	2	2
All-cause death	2	1	3
Progression to intubation and invasive ventilation	3	2	5
Total	6	12	18

Safety endpoints			
	Rivaroxaban	SOC	Total
Fatal bleeding	0	0	0
Non-fatal major bleeding according to ISTH Criteria	1	0	1
Clinically relevant non-major bleeding	2	3	5
Non-major bleeding with study-drug interruption for > 7 days	1	1	2

## Discussion

This open-label, multicenter, randomized, controlled clinical trial in patients with moderate to severe COVID-19 and a high prothrombotic risk defined by an elevated d-dimer at baseline demonstrated that d-dimer concentrations were not different between patients assigned to a seven days course of therapeutic anticoagulation with rivaroxaban or prophylactic anticoagulation with a heparin. The clinical outcome—as assessed by the seven-category ordinal scale by WHO—was not improved after therapeutic anticoagulation with rivaroxaban compared to prophylactic anticoagulation with a heparin in the overall study population. In an exploratory analysis of the patient subgroup with a very high risk as defined by d-dimer  > 1 mg/L at baseline, therapeutic anticoagulation with rivaroxaban, but not thromboprophylaxis with a heparin, was associated with an improvement on the 7-category ordinal scale by WHO (Table 7).d-dimer is a helpful marker in the management of anticoagulation, including evaluating the anticoagulation quality, predicting clinical outcomes, and determining the optimal duration and intensity of anticoagulation [22]. An elevated d-dimer was used as an eligibility criterion in the large MARINER trial [10] that evaluated the use of in-hospital and post-discharge VTE prophylaxis with rivaroxaban 10 mg in medically ill patients. This large phase III trial demonstrated the value of d-dimer as a risk stratification tool. In the present study, anticoagulation with rivaroxaban at a therapeutic dose of 20 mg daily for seven days reduced the d-dimer concentration, as did thromboprophylaxis with a heparin. There were no significant differences between the two treatment groups, pointing to the antithrombotic effectiveness of either treatment strategy for patients with moderate to severe COVID-19. In line, current ESC recommendations for antithrombotic therapy in patients hospitalized with moderate to severe COVID-19 suggest prophylactic anticoagulation as SOC [13, 14].

**Table 7:** Seven-category ordinal scale recommended by the WHO

7: Death
6: ICU, requiring invasive mechanical ventilation
5: ICU, not requiring invasive mechanical ventilation
4: Non-ICU, requiring oxygen
3: Non-ICU, not requiring oxygen
2: Discharged without resumption of normal activities
1: Discharged with resumption of normal activities

COVID-19 can be associated with mild, moderate and severe respiratory symptoms up to acute respiratory distress syndrome [23]. From the beginning of the pandemic, a severity classification based on the respiratory status of patients was proposed by the WHO [18, 19]. This score facilitated not only the prediction of clinical worsening during admission of patients with COVID-19, but also served as a guide for clinical trials that examined the effectiveness of different therapeutic strategies [24]. Nevertheless, those patients in COVID-PREVENT who received rivaroxaban at a therapeutic dose showed no significant alterations in the ordinal scale score at day 7 as compared to those who received thromboprophylaxis with a heparin (Table 5). Our data are line with the results of several other recent trials examining the strategies of anticoagulation in patients with COVID-19 [25] (Table 8). In ACTION, in-hospital therapeutic anticoagulation with rivaroxaban or enoxaparin followed by rivaroxaban to day 30 did also not improve clinical outcomes in patients with COVID-19 and elevated d-dimer [26]. In contrast to COVID-PREVENT, the d-dimer concentrations required for inclusion into ACTION had to be above the ULN reference only. The RAPID trial compared therapeutic vs prophylactic anticoagulation with heparin and focused on moderately ill patients with COVID-19 that had also an elevated d-dimer above the ULN reference only. In RAPID, there was again no significant difference in the 28-day composite of death, invasive mechanical ventilation, noninvasive mechanical ventilation, or ICU admission between the two treatment groups [27]. It is of note that the patients included into COVID-PREVENT exhibited a d-dimer of at least 1.5 ULN. An explorative analysis of COVID-PREVENT focusing on a subgroup of patients with even higher baseline d-dimer  > 2 ULN (or > 1 mg/L) revealed that rivaroxaban at a dose of 20 mg daily but not heparin at a prophylactic dose, was associated with improved clinical outcome as measured by the score (Table 5, Fig. 2). This points to the notion that initial rivaroxaban at therapeutic doses might be superior to thromboprophylaxis in patients with COVID-19 who exhibit a very high thrombotic risk as defined by d-dimer  > 2 ULN. This hypothesis is supported by data from the HEP-COVID trial, in which even higher elevated d-dimer level > 4 ULN or a sepsis-induced coagulopathy score of 4 or more were used as inclusion criteria [28]. Therapeutic anticoagulation with enoxaparin was superior to standard prophylactic or intermediate-dose anticoagulation with a heparin and led to a 32% reduction in the primary outcome of venous or arterial thromboembolism or death from any cause [28]. In addition, full-dose prophylactic anticoagulation held substantial benefit for moderately ill COVID-19 patients in the ACTIV-4a, ATTACC, and REMAP-CAP platform trials as well [15, 16].

**Table 8:** Results of main clinical trials evaluating anticoagulation strategies in COVID-19

Trial	n	Population	Intervention	Control	Primary outcomes	Safety outcomes	Main results
ACTION [26]	615	Hospitalized patients with COVID-19 with elevated d-dimer	Therapeutic anticoagulation for 30 days with rivaroxaban for stable patients or enoxaparin or UFH	Prophy-lactic anticoagu-lation with enoxaparin or UFH	Composite of time to death, duration of hospitalization, or duration of supplemental oxygen to day 30	Major or clinically relevant non-major bleeding through 30 days	No difference between patient-groups (win ratio 0.86 [95% CI 0.59–1.22], p = 0.40) Safety outcome in 26 patients (8%) of the intervention group vs. 7 patients (2%) of the control group (RR 3.64 [95% CI 1.61–8.27], p = 0.0010)
ACTIV-4B [30]	657	Symptomatic but clinically stable outpatients with COVID-19	Aspirin 81 mg orally, prophylactic-dose apixaban (2.5 mg orally twice daily), therapeutic-dose apixaban (5 mg orally twice daily)	Placebo	Composite of all-cause mortality, symptomatic venous or arterial thromboembo-lism, myocardial infarction, stroke, or hospitalization for cardiovascular or pulmonary cause to day 45	Major bleeding and clinically relevant non-major bleeding	Early termination due to lower than anticipated event rates Primary outcome in 1 patient (0.7%) in the aspirin group vs. 1 patient (0.7%) in the 2.5 mg apixaban group vs. 2 patients (1.4%) in the 5 mg apixaban group vs. 1 patient (0.7%) in the placebo group Risk difference compared with placebo 0.0% aspirin group, 0.7% (95% CI − 2.1 to 4.1%) in the 2.5 mg apixaban group, and 1.4% (95% CI − 1.5 to 5.0%) in the 5 mg apixaban group Risk differences compared with placebo for bleeding events were 2.0% in the aspirin group (95% CI -2.7% to 6.8%), 4.5% in the prophylactic apixaban-group (95% CI − 0.7 to 10.2%), and 6.9% in the therapeutic apixaban group (95% CI 1.4–12.9%)
COVID-Prevent	111	Patients with moderate to severe COVID-19 and d-dimer s > 1.5 ULN	Therapeutic anticoagulation with rivaroxaban 20 mg OD for 7 days followed by prophylactic dose of rivaroxaban 10 mg OD for 28 days	Prophy-lactic anticoagu-lation with LMWH or UFH until day 7 post randomiza-tion or discharge	Primary out-come d-dimer level at 7 days post randomization Co-primary efficacy outcome 7-category ordinal COVID-19 scale by WHO at 7 days post randomi-zation	Fatal or non-fatal major bleeding	d-dimer at 7 days was not different between groups (1.21 mg/L [0.79, 1.86] vs 1.27 mg/L [0.79, 2.04], p = 0.78) The 7-category ordinal scale was not different between the groups (relative effect 0.5922 [0.4873, 0.6971], Wilcoxon rank-sum test p = 0.085)
FREEDOM [25]	3398	Patients hospitalized with COVID-19 not requiring intensive care unit treatment	Therapeutic-dose enoxaparin, or therapeutic-dose apixaban	Prophy-lactic dose enoxaparin	Composite of all-cause mortality, requirement for intensive care unit–level of care, systemic thromboembo-lism, or ischemic stroke at 30 days	In- hospital rate of major bleeding	Primary outcome in 13,2% patients in the prophylactic-dose group vs. 11,3% in the combined therapeutic-dose groups (HR 0.85; 95% CI 0.69–1.04; p = 0.11) Major bleeding in all 3 groups was infrequent occurring in 0.1% and 0.4% of patients in the prophylactic-dose and therapeutic-dose anticoagulation groups, respectively
HEP-COVID [28]	253	Hospitalized adult patients with COVID-19 with d-dimer levels > 4 times ULN or sepsis-induced coagulopathy score of 4 or greater	Therapeutic—dose enoxaparin	Institutional standard prophy-lactic or interme-diate-dose LMWH or UFH	Composite of venous thromboembo-lism, arterial thromboembo-lism, or death from any cause at 30 days	Major bleeding at 30 ± 2 days	Primary outcome met in 41,9% of patients in the standard-dose vs. 28,7% of patients in the therapeutic-dose (RR 0.68; 95% CI 0.49–0.96; p = 0.03) Major bleeding: 1.6% with standard-dose vs 4.7% with therapeutic-dose heparins (RR 2.88; 95% CI 0.59–14.02; p = 0.17)
INSPIRATION [31]	562	Adult patients admitted to the ICU with COVID-19	Intermediate-dose prophylactic anticoagulation	Standard-dose prophy-lactic anticoagu-lation	Composite of venous or arterial thrombosis, treatment with extracorporeal membrane oxygenation, or mortality within 30 days	Major bleeding and severe thrombocyte-penia	No difference between the groups regarding primary outcome (odds ratio, 1.06 [95% CI 0.76–1.48]; p = 0.70) Major bleeding: 7 patients (2,5%) in the intermediate-dose group vs. 4 patients (1,4%) in the standard-dose group (p for non-inferiority > 0.99)
MI-CHELE [29]	320	Hospitalized patients with COVID-19 at increased risk for venous thrombo-embolism	Rivaroxaban 10 mg/day at hospital discharge	No anticoagu-lation at hospital discharge	Composite of symptomatic or fatal venous thromboembo-lism, asymptomatic venous thromboembo-lism on bilateral lower-limb venous ultrasound and CT pulmonary angiogram, symptomatic arterial thromboembo-lism, and cardiovascular death at day 35	Major bleeding	Primary efficacy outcome occurred in 5 (3%) of 159 patients assigned to rivaroxaban and 15 (9%) of 159 patients assigned to no anticoagulation (RR 0.33, 95% CI 0.12–0.90; p = 0.0293) No major bleeding occurred in either study group
Multi-platform (REMA-CAP, ACTIV-4a, ATTACC) in non-critically ill [15]	2219	Hospitalized with Covid-19 with absence of critical care–level organ support at enrollment	Therapeutic anticoagulation with heparin	Pharmaco-logic thrombo-prophylaxis	Organ support–free days up to day 21 post randomization	Major bleeding and laboratory-confirmed HIT	Probability that therapeutic-dose anticoagulation increased organ support–free days as compared with usual-care thromboprophylaxis was 98.6% (adjusted odds ratio, 1.27; 95% credible interval, 1.03–1.58) Major bleeding: 1.9% of the patients in the therapeutic-dose anticoagulation vs. in 0.9% of those receiving thromboprophylaxis
Multi-platform (REMA-CAP, ACTIV-4a, ATTACC) in critically ill [16]	1098	Critically ill patients with severe Covid-19	Therapeutic anticoagulation with heparin	Pharmaco-logic thrombo-prophylaxis	Organ support–free days up to day 21 post randomization	Major bleeding and laboratory-confirmed HIT	Probability of futility [defined as an odds ratio < 1.2], 99.9% (adjusted proportional odds ratio, 0.83; 95% credible interval, 0.67 to 1.03) Major bleeding: 3.8% of the patients in the therapeutic-dose anticoagulation vs. in 2.3% of those receiving thromboprophylaxis
RAPID [27]	465	Hospitalized patients with covid-19 and with increased d-dimer levels	Therapeutic dose heparin (low molecular weight or unfractionated heparin)	Prophy-lactic dose heparin (low molecular weight or unfractio-nated heparin)	Composite of death, invasive mechanical ventilation, non-invasive mechanical ventilation, or admission to an intensive care unit, assessed up to 28 days post randomization	Major bleeding	Primary composite outcome occurred in 16.2% patients assigned to therapeutic heparin vs. 21.9% assigned to prophylactic heparin (OR 0.69, 95% confidence interval 0.43–1.10; p = 0.12) Major bleeding occurred in 0.9% of patients assigned to therapeutic heparin and 1.7% assigned to prophylactic heparin (OR 0.52, 0.09–2.85; p = 0.69)

LMWH = low molecular weight heparin

UFH = unfractioned heparin

OD = once daily

ULN = upper limit of normal

The MICHELLE study provided also evidence for the value of d-dimer measurements to assess the thrombotic risk in patients with COVID-19 [24]. Patients hospitalized with COVID-19 at increased risk for venous thromboembolism, defined as an elevated modified International Medical Prevention Registry on Venous Thromboembolism (IMPROVE) venous thromboembolism (VTE) score of 4 or of 2–3 and a d-dimer  > 0.5 mg/L received, at hospital discharge, rivaroxaban 10 mg/day or no anticoagulation for 35 days [29]. The authors described that, in patients at high risk discharged after hospitalization due to COVID-19, thromboprophylaxis with rivaroxaban 10 mg/day for 35 days improved clinical outcomes compared with no extended thromboprophylaxis [29]. Before that study, the National Institutes of Health–sponsored ACTIV-4B Outpatient Thrombosis Prevention Trial, testing apixaban in mildly symptomatic COVID-19 outpatients, was halted prematurely after enrollment of 9% of the planned total number of participants due to the low incidence of thrombotic events in this population [30].

The recently presented FREEDOM trial reported that therapeutic anticoagulation with enoxaparin or apixaban was associated with a numerically lower composite 30-day risk of all-cause mortality, progression to ICU care, systemic thromboembolism, or ischemic stroke by a relative 15% compared with prophylactic dosing of enoxaparin, which was, however, not statistically significant [25]. Strikingly, therapeutic anticoagulation compared to prophylactic anticoagulation significantly reduced all-cause mortality by a relative 30% and intubation by a relative 25%, favoring full-dose anticoagulation for patients hospitalized with COVID-19 [25].

In COVID-PREVENT, the secondary endpoint—defined as either venous or arterial thromboembolism, new myocardial infarction, non-hemorrhagic stroke, all-cause death or progression to intubation and invasive ventilation up to 35 days post randomization—occurred in 6 patients (10.9%) in the rivaroxaban group and in 12 (21.4%) in the SOC group (Fig. 2). This numerical difference was not significant due to the small number of only 111 patients included into COVID-PREVENT. The absence of any significant change in incidence of the clinical efficacy endpoint in COVID-PREVENT is in line with the results of the recent ACTION and RAPID trial as discussed above. [26]. In addition, therapeutic anticoagulation in ACTION led to a higher incidence of ISTH-defined major or clinically relevant non-major bleeding than did prophylactic anticoagulation [26]. The NIH ACTIV trial and the INSPIRATION trial showed that critically ill patients admitted to ICU did not benefit from therapeutic compared to prophylactic anticoagulation due to increased bleeding rates [16, 31]. Clinically unstable patients were also enrolled into the ACTION trial. Therefore, the findings of ACTION are consistent with the findings of previous studies in this population. In COVID-PREVENT the decision to stop therapeutic anticoagulation with rivaroxaban upon ICU admission and to switch to a heparin at prophylactic doses was made by the treating physician based on local hospital standard of care and current guidelines. Only one patient who had a non-fatal major bleeding reached the primary safety outcome in our study. We assume that the awareness that the bleeding risk is heightened, and that careful handling of anticoagulation is necessary in critically ill patients with COVID-19, has increased. This might explain the low rate of the primary safety outcome in COVID-PREVENT. In line, the very recently published FREEDOM trial showed also low Bleeding Academic Research Consortium (BARC) types 2, 3, and 5 bleeding rates, reaching 0.1–0.5% across the different anticoagulation groups [25].

The COVID-PREVENT trial has a major limitation regarding the limited number of patients included into the study. The decreasing incidence of COVID-19 in Germany in March–April 2021 made it impossible to recruit the initially planned number of patients in a timely manner. Therefore, future trials with larger study populations are needed to confirm that high risk patients with an immunothrombotic disease such as COVID-19 benefit from therapeutic anticoagulation.

In conclusion, in patients with moderate to severe COVID-19, initial therapeutic anticoagulation was not different from thromboprophylaxis in affecting d-dimer. An exploratory analysis suggested that therapeutic anticoagulation with rivaroxaban compared to prophylactic anticoagulation with a heparin was associated with improved surrogates for clinical outcome in a COVID-19 subgroup with high thrombotic risk as defined by d-dimer  > 2 ULN.

### Supplementary Information

Below is the link to the electronic supplementary material.

Supplementary file1 (PDF 981 KB)
