# Supplementary material for: Initial therapeutic anticoagulation with rivaroxaban compared to prophylactic therapy with heparins in moderate to severe COVID-19: results of the COVID-PREVENT randomized controlled trial
Source: Clin Res Cardiol. 2023 Jul 5;112(11):1620–38. doi: 10.1007/s00392-023-02240-1 (PMC10584737; doi:10.1007/s00392-023-02240-1)
Supplement: Supplementary file 1 — Supplementary file1 (PDF 981 KB) [file 392_2023_2240_MOESM1_ESM.pdf]

Appendix 1

This supplement contains:

- Clinical Protocol, Version 2.0 p. 2

    - Summary of Changes p. 57

Clinical Protocol

Effect of anticoagulation therapy on clinical outcomes in COVID-19
(**COVID-PREVENT)**

EudraCT-Nb: 2020-002282-33

Sponsor: Charité – Universitätsmedizin Berlin

Representative of the sponsor and chief investigator: [redacted]

Version: **2.0**

This protocol version is an integration of the following documents / sections:

- Original protocol, Version 1.4, dated 30 OCT 2020
- Amendment 01 (described in Section 18.1)
  forming integrated protocol Version 2.0, dated 20th AUG 2021

Date: 20th August 2021

Prepared by: [redacted]

The following person accepts the content of this protocol and confirms to conduct this study in compliance with Good Clinical Practice and applicable regulatory requirements.

[redacted]

[redacted]

The following person – responsible for planning the statistical analysis in this protocol – accepts the content of this protocol and confirms to conduct this study on compliance with Good Clinical Practice and applicable regulatory requirements.

[redacted]

GCP Compliance:
This study will be conducted in compliance with Good Clinical Practice, and applicable regulatory requirements.

Confidentiality Statement:
The information in this document contains trade secrets and commercial information that are privileged or confidential and may not be disclosed unless such disclosure is required by applicable law or regulations. In any event, persons to whom the information is disclosed must be informed that the information is privileged or confidential and may not be further disclosed by them. These restrictions on disclosure will apply equally to all future information supplied to you that is indicated as privileged or confidential.

TABLE OF CONTENTS

TABLE OF CONTENTS **4**

LIST OF ATTACHMENTS **6**

LIST OF IN-TEXT TABLES AND FIGURES **6**

SYNOPSIS **7**

TIME AND EVENTS SCHEDULE **15**

ABBREVIATIONS **17**

1. INTRODUCTION **19**
1.1. Background **19**
1.1.1. Compound Profile 20
1.2. Overall Rationale for the Study **20**

2. OBJECTIVES AND HYPOTHESIS **21**

3. STUDY DESIGN AND RATIONALE **23**
3.1 Overview Study Design **23**
3.2. Study Design Rationale **25**
3.3. Committees **26**
3.3.1. Executive Committee (EC) 26
3.3.2. Independent Data Safety Monitoring Board (DSMB) 26
3.3.3. Clinical Events Committee (CEC) 28

4. SUBJECT POPULATION **28**
4.1. Inclusion Criteria **28**
4.2. Exclusion Criteria **28**
4.3. Prohibitions and Restrictions **30**

5. TREATMENT ALLOCATION AND BLINDING **30**

6. DOSAGE AND ADMINISTRATION **31**

7. TREATMENT COMPLIANCE **31**

8. PRESTUDY AND CONCOMITANT THERAPY **32**

9. STUDY EVALUATIONS **32**
9.1. Study Procedures **32**
9.1.1. Overview 32
9.1.2. Screening Phase 32
9.1.3. Treatment Phase 33
9.2. Efficacy Evaluations and Outcomes **36**
9.2.1. Efficacy Evaluations, Outcomes and Adjudication 36
9.2.2. Approach to the Subject with a secondary Efficacy Endpoint Event 36
9.3. Safety Evaluations and Endpoints **37**
9.3.1. Bleeding Events 37
9.3.2. Approach to the Subject with a Bleeding Event 37
9.3.3. Other Safety Assessments 38
9.4. Benefit-Risk Assessment **38**

10. SUBJECT COMPLETION / WITHDRAWAL **38**
10.1. Completion **38**

10.2. Discontinuation of Study Treatment **39**
10.2.1. Temporary Interruption of Study Treatment 39
10.2.2. Approach to Subjects with Temporary Interruption of Study Treatment 39
10.2.3. Permanent Discontinuation of Study Treatment 40
10.3. Withdrawal from the Study **41**

11. STATISTICAL METHODS **41**
11.1 Definition of populations included in the analyses **41**
11.2 Primary and Co-primary Efficacy Endpoints **41**
11.3 Secondary Efficacy Endpoint **42**
11.7 Sample size determination **43**

12. ADVERSE EVENT REPORTING **44**
12.1 Adverse Events (AEs) **44**
12.1.1. Definitions 44
12.1.2 Classification for Adverse Events (AE) assessment 45
12.1.3. Assessments and documentation of AEs 46
12.2. Reporting of SAEs/AEs **46**
12.3. Procedures **47**
12.3.1 Reporting Timelines and Processes 47
12.3.2. SAEs and AESI reporting 47
12.3.3. SUSAR reporting 48
12.3.4. Data Safety Monitoring Board (DSMB) / Clinical Events Committee (CEC) 48
12.3.5. Pregnancy 48
12.4. Contacting Sponsor Regarding Safety **48**

13. PRODUCT QUALITY COMPLAINT HANDLING **49**
13.1. Procedures **49**
13.2. Contacting Sponsor Regarding Product Quality **49**

14. STUDY DRUG INFORMATION **49**
14.1. Physical Description of Study Drug(s) **49**
14.2. Preparation Handling and Storage **49**
14.3. Drug Accountability **49**

15. ETHICAL ASPECTS **50**
15.1. Study-Specific Design Considerations **50**
15.2. Regulatory Ethics Compliance **50**
15.2.1. Investigator Responsibilities 50
15.2.2. Independent Ethics Committee or Institutional Review Board 50
15.2.3. Informed Consent 51
15.2.4. Privacy of Personal Data 52

16. ADMINISTRATIVE REQUIREMENTS **52**
16.1. Protocol Amendments **52**
16.2. Regulatory Documentation **53**
16.2.1. Regulatory Approval/Notification 53
16.2. Subject Identification Enrollment and Screening Logs **53**
16.3. Source Documentation **53**
16.4. Case Report Form Completion **53**
16.5. Data Quality Assurance/Quality Control **53**
16.6. Record Retention **54**
16.7. Monitoring **54**
16.8. Study Completion/Termination **54**
16.8.1. Study Completion 54
16.8.2. Study Termination 55
16.9. On-Site Audits **55**
16.10. Use of Information and Publication **55**

17. SUBSTUDIES **55**
17.1. Bio-banking (optional) **55**

18. PROTOCOL AMENDMENTS 56
18.1. Amendment 1 56
18.1.1 Overview of changes 56

19. REFERENCES 63

LIST OF ATTACHMENTS

Attachment 1: Lower-dose prophylactic anticoagulation 65
Attachment 2: Seven category ordinal scale recommended by the WHO (23-25) 66
Attachment 3: CKD-EPI Formula 67
Attachment 4: Secondary Efficacy endpoints 68
Attachment 5: Clinical Classification of Severity of COVID-19 70

LIST OF IN-TEXT TABLES AND FIGURES

Table 1: Preoperative Interruption of Rivaroxaban: A Suggested Management Approach (adapted from Spyropoulos and Douketis 2012) 4040
Table 2: Postoperative Resumption of Rivaroxaban: A Suggested Management Approach 40

FIGURE

Figure 1: Schematic Overview of the Study (COVID-PREVENT) 25

SYNOPSIS

Effect of anticoagulation therapy on clinical outcomes in moderate to severe coronavirus disease 2019 (COVID-19) (**COVID-PREVENT)**

DESCRIPTION OF THE COMPOUND

Rivaroxaban is an oral, direct acting, Factor Xa (FXa) inhibitor anticoagulant that has been under development for the treatment of several thrombosis-mediated conditions. Rivaroxaban is marketed under the trade name XARELTO® and has been approved for multiple indications in more than 130 countries. The clinical development program for rivaroxaban is extensive. Subjects have been exposed to rivaroxaban in completed and ongoing interventional clinical trials and non-interventional studies, with the total daily doses of rivaroxaban ranging between 5 mg and 60 mg.

OBJECTIVE AND HYPOTHESIS

Primary Objective

The primary objective is to assess the effect of rivaroxaban compared with standard of care (SOC) with the use of prophylactic low molecular weight heparin (LMWH) or unfractionated heparin (UFH) (see Attachment 1) on D-dimer as a clinical marker for the clinical outcome at day 7 post randomization adjusted for baseline measurement in patients with moderate to severe COVID-19.

The co-primary objective is to evaluate the impact of rivaroxaban compared with standard of care (SOC) with the use of prophylactic low molecular weight heparin (LMWH) or unfractionated heparin (UFH) on a seven-category (Attachment 2) ordinal scale recommended by the WHO (23-25) as a measure of clinical benefit at day 7 post randomization adjusted for baseline score in patients with moderate to severe COVID-19.

Primary Endpoint

- D-dimer at day 7 post randomization

Co-primary Endpoint

- Seven-category ordinal scale recommended by the WHO at day 7 post randomization

Secondary Objectives

The secondary objective is to assess the efficacy and safety of rivaroxaban compared with standard of care (SOC) (if appropriate including the use of prophylactic low molecular weight heparin (LMWH) or unfractionated heparin (UFH) (see Attachment 1)) in the prevention of the composite endpoint described below up to day 35 in patients with moderate to severe COVID-19:

- Venous thromboembolism (VTE) (deep venous thrombosis (DVT) and/or fatal or non-fatal
- pulmonary embolism (PE))
- Arterial thromboembolism
- New myocardial infarction (MI)
- Non-hemorrhagic stroke
- All-cause mortality (ACM)
- Progression to intubation and invasive ventilation

Secondary combined Endpoint

Time to first event of either of

- Venous thromboembolism (VTE) (deep venous thrombosis (DVT) and/or fatal or non-fatal pulmonary embolism (PE))
- Arterial thromboembolism
- New myocardial infarction (MI)

- Non-hemorrhagic stroke
- All-cause death
- Progression to intubation and invasive ventilation

In addition, the components VTE, arterial thromboembolism, MI, non-hemorrhagic stroke and all-cause-death of the secondary endpoint will be additionally evaluated, if sufficient numbers of events are observed.

Safety Objectives

The safety objectives are to compare rivaroxaban with SOC with prophylactic LMWH or UFH in bleeding outcomes up to day 35 in patients with moderate to severe COVID-19.

Primary Safety Endpoints:

- Fatal and non-fatal major bleeding using the International Society on Thrombosis and Haemostasis (ISTH) bleeding criteria

Secondary Safety Endpoints:

- Clinically relevant non-major bleeding
- Non-major bleeding that lead to study-drug interruption for more than 7 days

Other endpoints of interest:

- Length of hospital stay
- Time to intubation
- Re-hospitalization due to heart failure
- Re-hospitalization due to any other reason
- Effects on coagulation parameters for thrombosis (TAT, PAI-1, PF-4, TF, TF-activity)
- Effects on inflammatory and fibrotic parameters (interleukines, interferons, growth factors)
- Change of N-terminal prohormone brain natriuretic peptide (NT-pro-BNP)
- Unscheduled outpatient visits

Hypotheses

The primary hypothesis is that rivaroxaban is **more effective than SOC with prophylactic LMWH or UFH** in reducing D-dimer levels at day 7 post randomization compared to baseline in patients with moderate to severe COVID-19. The co-primary hypothesis is that rivaroxaban compared to SOC with prophylactic LMWH or UFH is superior to improve the patients' categories on a seven-category ordinal scale (Attachment 2) for patients with respiratory infections at day 7 post randomization.

The secondary hypotheses are that therapeutic anticoagulation with rivaroxaban for at least 7 days, followed by prophylactic anticoagulation with rivaroxaban for 28 days is superior to SOC with prophylactic LMWH or UFH in the prevention of the composite of VTE (DVT and/or fatal or non-fatal PE), arterial thromboembolism, new MI, non-hemorrhagic stroke, all-cause death and progression to intubation and invasive ventilation up to day 35 post randomization in patients with moderate to severe COVID-19.

OVERVIEW OF STUDY DESIGN

This is a multicenter, prospective, randomized, event-driven phase II study designed to assess the effect of therapeutic anticoagulation with high dose rivaroxaban intended for at least 7 days compared with SOC with prophylactic LMWH or UFH on the clinical prognosis marker D-dimer at day 7 post randomization adjusted for baseline measurement in patients with moderate to severe COVID-19. Moreover, this study investigates the effect of therapeutic anticoagulation with rivaroxaban compared

with SOC with prophylactic LMWH or UFH on a seven-category ordinal scale recommended by the WHO for patients with respiratory infections at day 7 post randomization adjusted for baseline score.

Furthermore, this trial is designed to evaluate therapeutic anticoagulation with high dose rivaroxaban for at least 7 days followed by thromboprophylaxis with medium dose rivaroxaban for 28 days, as compared with SOC with prophylactic LMWH or UFH in the prevention of the composite of VTE (DVT and/or fatal or non-fatal PE), arterial thromboembolism, new MI, non-hemorrhagic stroke, all-cause death and progression to intubation and invasive ventilation up to day 35 post randomization in patients with moderate to severe COVID-19. The study consists of a screening phase, a therapeutic treatment phase of at least 7 days and a thromboprophylaxis phase of 28 days up to day 35 post randomization. The physicians of the respective trial site decide which heparin, of the heparins approved in Germany, the patients assigned to treatment group "SOC with prophylactic UFH or LMWH" will receive.

The subject population comprises men and women aged 18 years and older who have been tested positive for severe acute respiratory syndrome coronavirus 2 (SARS-CoV-2) and who have moderate to severe COVID-19.

In order to be eligible, all patients must exhibit at least one of the following conditions:

- D-dimer elevation > 1.5 upper limit of normal (ULN, using age-adjusted cut-offs) and/or
- Cardiac injury reflected by an elevation in hs-cTnT (high sensitive cardiac troponin T) > 2.0 ULN and one of the following conditions
    - Known coronary artery disease (CAD)
    - Known diabetes mellitus
    - Active smoking

Any patient with a medical condition that requires use of any therapeutic parenteral or therapeutic oral anticoagulation (e.g. atrial fibrillation) is not eligible for participation. Moreover, patients at particularly increased risk of bleeding and those using medications that might interact with the study drug will be excluded.

Subject with moderate to severe respiratory symptoms, tested positive for SARS-CoV-2, can be enrolled into the study, if the subjects meet all of the inclusion and none of the exclusion criteria. The subjects will be randomly assigned to receive rivaroxaban or SOC with prophylactic LMWH or UFH.

Randomization can occur during the hospital stay or out-of-hospital, if admission to the hospital is not necessary, whatever the treating physician judges.

After randomization, subjects will receive treatment with rivaroxaban 20 mg (15 mg for subjects with an estimated glomerular filtration rate (eGFR) ≥30 mL/min/1.73m^2 and <50 mL/min/1.73m^2 (calculated with the Chronic Kidney Disease Epidemiology Collaboration (CKD-EPI) formula, see Attachment 3)) once daily (OD) for at least 7 days or SOC with prophylactic LMWH or UFH. In case of hospitalization for more than 7 days, the therapeutic treatment with rivaroxaban will be continued for the duration of the hospital stay until discharge. After at least 7 days of therapeutic treatment with rivaroxaban or after hospital discharge, whatever is later, the dose of rivaroxaban will be reduced to 10 mg OD and, then, thromboprophylaxis will be given for a duration of 28 days. Thereafter, the patients will continue medical therapy according to local practice. A complete end of study (EOS) visit will be performed after 60 days post randomization.

If the clinical condition of a hospitalized patient deteriorates and admission to intensive care unit (ICU) with possible invasive ventilation will become necessary, treatment with rivaroxaban should be discontinued for the ICU period and the patient switched to parental or subcutaneous anticoagulation following best local practice. After clinical improvement and discontinuation of invasive ventilation, the anticoagulation mentioned above can be replaced by study drug rivaroxaban 20 mg OD (15 mg for subjects with an eGFR ≥30 mL/min/1.73m^2 and <50 mL/min/1.73m^2) at the discretion of the clinically responsible physician.

The dosage of the study medication will be adjusted upon hospital discharge of the patients as follows. Patients randomized to rivaroxaban 20 mg OD will reduce daily dosage to 10 mg OD, provided that they were not diagnosed with a condition requiring continued therapeutic anticoagulation.

Patients randomized to the SOC arm will receive SOC with prophylactic LMWH or UFH according to local practice, provided that they were not diagnosed with a condition requiring continued therapeutic anticoagulation. Study visits are terminated 7, 35, and 60 days post randomization. The 60 day EOS visit will be done as a telephone visit.

If a subject has a suspected efficacy or bleeding outcome event during the study, the treating physician should clinically judge and follow established guidelines to apply best medical care.

The primary endpoint is to assess the effect of rivaroxaban compared with standard of care (SOC) with the use of prophylactic low molecular weight heparin (LMWH) or unfractionated heparin (UFH) on D-dimer as a clinical marker for a worse clinical outcome at day 7 post randomization adjusted for baseline measurement in patients with moderate to severe COVID-19.

The co-primary endpoint is to evaluate the impact of rivaroxaban compared with standard of care (SOC) with the use of prophylactic low molecular weight heparin (LMWH) or unfractionated heparin (UFH) on a seven-category ordinal scale recommended by the WHO as a measure of clinical benefit at day 7 post randomization in patients with moderate to severe COVID-19.

Sample size calculation is based on the primary endpoint D-dimer at day 7 corrected for baseline values. Since the distribution of D-dimer concentrations is skewed, calculations are done on the log scale. Using an analysis of covariance (ANCOVA) (significance level alpha=0.05 two-sided) with baseline values as covariate a total sample size of 80, i.e. 40 per group, gives a power of 80% to detect a mean difference of 0.44 (on the log scale) between the treatment group and the control group at day 7. Based on a blinded review, a common standard deviation of SD=0.8 and a correlation to baseline of r=0.5 (both on the natural log scale) were assumed. The effect assumed translates to a reduction of 36% in D-Dimer values on the original scale. The co-primary endpoint is a seven-category ordinal scale previously recommended by the WHO (Attachment 2) as clinical improvement scale for patients with respiratory infections. Here 40 patients per group will give a power of 80% to detect a relative treatment effect p1=0.68 between treatment and control group at day 7 using a Wilcoxon (Mann-Whitney) rank-sum test (two sided, alpha=0.05). The probabilistic index or relative effect p1 denotes the probability that an observation in the treatment group will be less than an observation in the control group. Targeting a total of at least 80 evaluable patients, we aim to recruit about 100 patients.

DOSAGE AND ADMINISTRATION

Treatment groups in this study are rivaroxaban and SOC with prophylactic LMWH or UFH. Subjects will be randomly assigned in a 1:1 ratio to receive rivaroxaban 20 mg OD (or 15mg OD in subjects with an eGFR ≥30 mL/min/1.73m^2 and <50 mL/min/1.73m^2) or SOC. The date and time of the first dose of study drug and the last dose of LMWH or UFH should be recorded as accurately as possible. The physicians of the respective trial site decide which heparin, of the heparins approved in Germany, the patients assigned to treatment group "SOC with prophylactic UFH or LMWH" will receive. Dosage specifications are listed in Attachment 1.

All subjects randomized to receive rivaroxaban should take rivaroxaban at approximately the same time each day. A missed dose should be taken as soon as possible (up to 8 hours prior to the next scheduled dose), and the next scheduled dose should be taken at the regular time. Study drugs may be interrupted temporarily as necessary for invasive procedures or as medically needed (such as in the setting of a bleeding event or a required prohibited therapy).

In that case, study drug needs to be temporarily interrupted and can be restarted at the discretion of the investigator. These interruptions will be recorded in the electronic case report form (eCRF). During the study, should the subject develop any condition, which in the investigator's judgment requires long-term full anticoagulation, or even fibrinolysis, the subject will have study treatment either temporarily interrupted or permanently discontinued and will be managed as deemed appropriate by the treating physician. The subject will be asked to continue in the study to be followed for efficacy and safety outcomes.

EFFICACY EVALUATIONS/ENDPOINTS

The primary efficacy endpoint is defined as D-dimer at day 7 post randomization adjusted for baseline.

Co-primary endpoint is the seven-category ordinal scale recommended by the WHO at day 7 post randomization adjusted for baseline score.

The secondary efficacy endpoint is defined as time to first event of either of the following components:

- VTE (DVT and/or fatal or non-fatal PE)
- Arterial thromboembolism
- New MI
- Non-hemorrhagic stroke
- All-cause death
- Progression to intubation and invasive ventilation

In addition, the components VTE, arterial thromboembolism, MI, non-hemorrhagic stroke and all-cause-death of the secondary endpoint will be additionally evaluated, if the numbers of events are sufficiently large.

Any clinical event that suggests the possibility that a secondary efficacy outcome event has occurred should be indicated on the Clinical Status page of the eCRF and will be sent for adjudication.

Other endpoints of interest:

- Length of hospital stay
- Time to intubation
- Re-hospitalization due to heart failure
- Re-hospitalization due to any other reason
- Effects on coagulation parameters for thrombosis (TAT, PAI-1, PF-4, TF, TF-activity)
- Effects on inflammatory and fibrotic parameters (interleukines, interferons, growth factors)
- Change of N-terminal prohormone of brain natriuretic peptide (NT-pro-BNP)
- Unscheduled out-patient visits

Secondary efficacy and other endpoints of interest are defined in Attachment 4.

SAFETY EVALUATIONS/ ENDPOINTS

- The principal safety endpoints are fatal or non-fatal major bleeding as defined according to the ISTH classification.

The secondary safety endpoints are the following:

- Clinically relevant non-major bleeding
- Non-major bleeding that lead to study drug interruption for more than 7 days.

An ISTH major bleeding event is defined as overt bleeding associated with: a fall in hemoglobin of 2 g/dL or more, or leading to a transfusion of 2 or more units of packed red blood cells or whole blood, or bleeding that occurs in a critical site: e.g., intracranial, intraspinal, intraocular, pericardial, intra-articular, intramuscular with compartment syndrome, retroperitoneal, or a fatal outcome (for details on definition see Section 9.3.1.

Non-major clinically relevant bleeding is defined as overt bleeding not meeting the criteria for major bleeding, but associated with medical intervention, unscheduled contact (visit or telephone call) with a physician, (temporary) cessation of study treatment, or associated with discomfort for the subject such as pain or impairment of activities of daily life.

Other bleeding is defined as any other overt bleeding that does not meet the ISTH criteria for major or non-major clinically relevant bleeding.

Any clinical event that suggests the possibility that a bleeding outcome event has occurred will be sent for adjudication.

Stopping rules / discontinuation

The patient reassessment for clinical events objective is to assess the incidence of clinically relevant events (including secondary efficacy endpoints and all safety endpoints). The study will be discontinued if the Sponsor or its designee judges it necessary for medical, safety, regulatory, or other reasons consistent with applicable laws, regulations, and GCP guidelines.

The following events, if applicable, may cause premature termination of the clinical study:

- Early evidence of overt inferiority of the treatment according to the recommendation of the Data Safety Monitoring Board (DSMB) (decision taken by the Sponsor);
- Unjustifiable risk and/or toxicity in risk-benefit analysis (decision taken by the Sponsor), e.g. when adverse events occur, unknown to date with respect to their nature, severity, duration or frequency in relation to the currently established safety profile (substantial changes to the risk-benefit ratio), and therefore medical and/or ethical reasons affect the continuation of the study;
- New scientific evidence provided during the study that could affect the patient's safety (benefit-risk analysis no longer positive);
- Request of the Sponsor or regulatory agency.

STATISTICAL METHODS

This is an explorative study and hence analyses have an explorative nature.

Definition of populations included in the analyses

The primary efficacy analysis as well as all secondary efficacy analyses will be based on the full analysis set (FAS) following the intention-to-treat principle and will include all randomized patients. Safety analyses will be based on the safety set (SAF) including all randomized patients with at least one dose of study medication or at least one dose of SOC (UFH or LMWH).

For time-to-event endpoints, study discontinuations will be dealt with as independent right censoring. Patients discontinuing study drug will be followed up for endpoints. Otherwise missing values will be dealt with using multiple imputation methods.

Sensitivity analyses will be performed in all patients (a) completing at least 7 days of treatment and (b) completing the study adherent to the study protocol. These will be referred to as the (a) while on treatment population and (b) per-protocol (PP) population.

Primary and Co-primary Efficacy Endpoints

The primary efficacy endpoint is D-dimer at day 7 post randomization will adjusted for baseline measurement. The logarithmic D-dimer measurements (using the natural logarithm) will be analyzed by an analysis of covariance (ANCOVA). The model will include treatment group and stratification variables of the randomization as factors and the logarithmic baseline D-dimer measurement as covariate. Least squares means for D-dimer at day 7 in the two treatment groups will be presented with 95% confidence intervals as well as the difference between the treatment groups at day 7 with 95% confidence interval and p-value testing the null hypothesis of no treatment difference, i.e. a mean difference (on the logarithmic scale) of 0. For ease of interpretation the results will also be converted to the original measurement scale.

The co-primary endpoint is the seven-category ordinal scale recommended by the WHO (Attachment 2) at day 7 post randomization adjusted for baseline score. Frequencies and percentages of the scores at day 7 will be provided stratified by treatment group. The distributions of the scores will be compared between the two treatment groups using a nonparametric Wilcoxon (Mann-Whitney) rank-sum test stratified by baseline score dichotomized as smaller or equal 3 vs. larger 3. The treatment effect will be reported as probabilistic index or relative effect, i.e. the probability that an observation in the treatment group will be smaller (more favourable) than an observation in the control group, with 95% confidence interval.

Secondary Efficacy Endpoints

Secondary composite endpoint includes

Time to first event of either of

- Venous thromboembolism (VTE) (deep venous thrombosis (DVT) and/or fatal or non-fatal pulmonary embolism (PE))
- Arterial thromboembolism
- New myocardial infarction (MI)
- Non-hemorrhagic stroke
- All-cause death
- Progression to intubation and invasive ventilation

as well as each component of the secondary composite endpoint as described above, if sufficient numbers of events will be observed.

Time to first event of either of the components of the secondary composite endpoint will be analyzed using a Cox proportional hazards regression model with treatment and the stratification variables of the randomization (site, gender (male, female, diverse), age (< 65 versus ≥ 65 years), kidney function (eGFR ≥ 30 mL/min/1.73m^2 and < 50 mL/min/1.73m^2 versus eGFR ≥ 50 mL/min/1.73m^2), history of CAD or heart failure (yes/no), oxygen demand on admission to the hospital (supplementary oxygen required versus not required), setting (outpatients versus hospitalized patients)) as factors. The treatment effect will be reported as hazard ratio (HR) with 95% confidence interval and p-value for the null hypothesis H0: HR=1. The primary endpoint will be visualized as Kaplan-Meier curves. Sensitivity analyses will explore the robustness of these analyses.

The analyses of the time-to-event outcomes among the secondary endpoints (such as time to VTE, time to arterial thromboembolism, time to new MI, time to non-hemorrhagic stroke, time to all-cause death, time to progression to intubation and invasive ventilation will follow the same lines as the analysis of the composite endpoint, i.e. survival analyses comprising Cox proportional hazard regression models and Kaplan-Meier curves. Death will be treated as a competing event except when analyzing all-cause death, naturally.

Longitudinal D-dimer measurements assessed at day 7, 35 and 60 will be analyzed on the logarithmic scale using a mixed model for repeated measures (MMRM) approach. The model will include treatment, visit, treatment-by-visit interaction and stratification variables of the randomization as factors as well as logarithmic D-dimer baseline value as covariate. The residuals will be assumed to follow a multivariate normal distribution with unstructured covariance matrix. LS means of the group means and their differences will computed for the various timepoints and reported with 95% confidence intervals.

Longitudinal ordinal data (seven-category ordinal scale) assessed at day 7, 35 and 60 will be analyzed using a proportional odds model with treatment, visit, treatment-by-visit interaction, baseline score and stratification variables of the randomization as factors. The correlation in longitudinal assessments within the same patient will be accounted for by random patient effects. Binary data (e.g. development of DIC) will use similar generalized linear mixed effect models but with logit link function.

All stratification variables of the randomization (site, gender (male, female, diverse), age (< 65 versus ≥ 65 years), kidney function (eGFR ≥ 30 mL/min/1.73m^2 and < 50 mL/min/1.73m^2 versus eGFR ≥ 50 mL/min/1.73m^2), history of CAD or heart failure (yes/no), oxygen demand on admission to the hospital (supplementary oxygen required versus not required), setting (outpatients versus hospitalized patients)) will be included as factors in the analyses of the secondary endpoints.

Subgroup analyses

Subgroup analyses are planned to explore a possible heterogeneity of the treatment effect concerning the following baseline characteristics: setting (outpatients versus hospitalized patients), gender (male, female, diverse), age (< 65 versus ≥ 65 years), kidney function (eGFR ≥ 30 mL/min/1.73m^2 and < 50 mL/min/1.73m^2 versus eGFR ≥ 50 mL/min/1.73m^2), smoking status, diabetes, presence of coronary heart disease or heart failure (yes/no), need for oxygen treatment at baseline (supplementary oxygen required versus not required), inclusion based on D-dimer, hs-cTnT or both. For subgroup analyses the respective baseline characteristic and its interaction with treatment are included in the regression

models described above for the primary and secondary endpoints. Treatment effect heterogeneity across subgroups will also be visually inspected in forest plots.

Safety Endpoints

Safety analyses will follow standard procedures for the reporting of adverse events; these will be reported as frequencies (percentages) by treatment group. All safety analyses will be based on the safety set. One safety endpoint of particular interest are bleeding events (see Section 9.3.1). The time to bleeding events will be analyzed in a Cox proportional hazards model, if a sufficient number of events will be observed. Treatment group differences will be reported as hazard ratios with 95% confidence intervals and p-values testing the null hypothesis of no group difference (i.e. HR=1). The estimation of the probability for bleeding events will account for competing events such as death and premature study discontinuation using the Aalen-Johansen estimator, the so-called cumulative incidence function.

Differences between treatment groups in continuous laboratory parameters and vital signs over follow-up time will be explored in longitudinal box plots.

TIME AND EVENTS SCHEDULE

Period	Screening[a]	Randomization	Therapeutic anticoagulation		Prophylactic anticoagulation	Follow-up
Visit	Screen	Rand	Day 7	Discharge[b]	Endpoint visit	EOS visit[c]
Days from randomization		0	7	Discharge day (variable)	35 days post Rand	60 days post Rand
Day window	-3/ 0	0	-2/ +1	-2/ 0	-0/+5	-3/+4
Informed consent	X					
Inclusion/Exclusion[d]	X	X				
SARS-CoV-2 test +[e]	X					
Physical Exam and Vitals[f]	X		X	X	X	(X)
D-dimer[g,h]	X		X	X	X	(X)
hs-cTnT[g,h]	X					
Demographics	X					
Medical History	X					
Hemoglobin/Platelet count[g]	X		X	X	X	
Serum creatinine[g]	X		X	X	X	
Serum/urine Pregnancy[i]	X					
Further safety blood assessment[j]	X		X		X	
ECG	X		X	X	(X)	
Registration		X				
Dispense Study Drug		X	X	X		
Study drug accountability			X	X	X	
Adverse Events		X	X	X	X	X
CTPA[k]			(X)	(X)	(X)	
Seven-category ordinal scale			X	X	X	X
Endpoint assessment			X	X	X	X
Concomitant Medications	X	X	X	X	X	X
Blood sampling[l]		(X)	(X)	(X)	(X)	(X)

EOT= end of treatment; EOS = end of study; hs-cTnT= high sensitive cardiac troponin T; ECG= electrocardiogram; CTPA= Computed tomography pulmonary angiogram

a. The time between the confirmation of a positive SARS-CoV-2 test and screening should not exceed 14 days.
b. If the patient is discharged later than day 10 post randomization a discharge visit becomes mandatory.
c. This visit may be performed as telephone visit.
d. Screening inclusion / exclusion criteria should be assessed and verified at randomization.
e. The subject might be eligible for the study, if the SARS-CoV-2 polymerase chain reaction (PCR) test had been positive within 14 days prior to randomization. There is no need to repeat the SARS-CoV-2 PCR test in the screening period.

f. Vital signs include pulse, blood pressure, temperature, respiratory rate and oxygen saturation.
g. Hemoglobin, platelet count, serum creatinine, D-dimer, hs-cTnT and white blood cell differential will be obtained by the local laboratory.
h. D-dimer and hs-cTnT values from up to 3 days prior to screening can be used.
i. Pregnancy test must be performed in women of child-bearing potential.
j. Further safety blood assay comprises aspartate aminotransferase (AST), alanine aminotransferase (ALT), international normalized ratio (INR), activated partial thromboplastin time (aPTT), high sensitive C reactive protein (hs-CRP), NT-pro-BNP and should be done by local laboratory.
k. A CTPA is highly recommended if the patient has severe symptoms or deteriorates.
l. Possible blood sampling for bio-banking: Serum, heparin, citrate and Ethylendiamintetraessigsäure (EDTA) blood for coagulation analysis and molecular analysis (not mandatory). Samples should be sent to Charité Campus Benjamin Franklin.

ABBREVIATIONS

aPTT	activated Partial Thromboplastin Time
ALT	alanine aminotransferase
AST	aspartate aminotransferase
AT	arterial thromboembolism
CAD	coronary artery disease
CKD-EPI	Chronic Kidney Disease Epidemiology Collaboration
CEC	Clinical Event Committee
CI	Cardiac index
CI	confidence interval
COVID-19	coronavirus disease 2019
CT	computed tomography
CTPA	computed tomography pulmonary angiogram
CV	cardiovascular
CYP3A4	Cytochrome P450 3A4
DIC	disseminated intravasal coagulation
DSMB	data safety monitoring board
DVT	deep vein thrombosis
eGFR	estimated glomerular filtration rate
eCRF	electronic case report form
eDC	electronic data capture
EDTA	Ethylendiamintetraessigsäure
EC	Executive Committee
ECG	electrocardiogramm
EOS	end of study
EOT	end of treatment
ESC	European Society of Cardiology
EU	European Union
FXa	factor Xa
FXIa	factor XIa
GCP	Good Clinical Practice
HR	hazard ratio
hs-TnT	high sensitive cardiac troponin T
hsCRP	high sensitive C reactive protein
ICF	informed consent form
ICH	International Conference on Harmonisation
ICU	intensive care unit
IEC	Independent Ethics Committee
INR	international normalized ratio
IRB	Institutional Review Board
ISTH	International Society on Thrombosis and Haemostasis
ITT	intention-to-treat
i.v.	intravenous
LMWH	low molecular weight heparin
MI	myocardial infarction
NOAC	non vitamin K antagonist oral anticoagulants
NT-pro-BNP	N-terminal prohormone of brain natriuretic peptide
OD	once daily
PAD	peripheral artery disease
PCR	polymerase chain reaction
PE	pulmonary embolism
P-gp	combined P-glycoprotein
PQC	product quality complaint
SARS-CoV-2	severe acute respiratory syndrome coronavirus 2
s.c.	subcutaneous
SmPC	summary of product characteristics
SOC	standard of care
SUSAR	suspected unexpected serious adverse reaction
TSAT	transferrin saturation
UFH	unfractionated heparin
ULN	upper limit of normal

VTE	venous thromboembolism
V-Q	ventilation-perfusion
WHO	world health organization

1. INTRODUCTION

Rivaroxaban is an oral, direct acting, FXa inhibitor anticoagulant that has been under development for the treatment of several thrombosis-mediated conditions. Rivaroxaban is marketed under the trade name XARELTO® and has been approved for multiple indications in more than 130 countries. The clinical development program for rivaroxaban is extensive. Over 100,000 subjects have been studied from Phase 1 through multiple large Phase 4 studies, covering several indications and potential indications in the overall clinical development program. Subjects have been exposed to rivaroxaban in interventional clinical trials and non-interventional studies, with the total daily doses of rivaroxaban ranging between 5 mg and 60 mg.

Rivaroxaban has been approved for the reduction in the risk of stroke and systemic embolism in patients with non-valvular atrial fibrillation, for the treatment of DVT and PE, for the reduction in the risk of recurrence of DVT and/or PE in patients at continued risk for recurrent DVT and/or PE after completion of initial treatment lasting at least 6 months, and for the prophylaxis of DVT, which may lead to PE in patients undergoing knee or hip replacement surgery. Moreover, it has been used in combination with aspirin, to reduce the risk of major cardiovascular events (cardiovascular (CV) death, MI and stroke) in patients with chronic CAD or peripheral artery disease (PAD)(1-3).

1.1. Background

The ongoing pandemic of COVID-19, caused by SARS-CoV-2 is associated with significant cardiovascular morbidity and mortality as the number of infected cases is increasing world-wide. The majority of moderate to severely ill patients initially present with respiratory failure, and may progress to a systemic disease associated with markedly increased risk of thromboembolism, DIC and CV events, as observed already in China, Italy and also Germany (4) (5, 6).

A significant clinical feature of poor prognosis in these patients is the development of a prothrombotic state. It has previously been observed that in patients who develop sepsis from various infectious agents, occurrence of a prothrombotic state is a major predictor of a poor clinical outcome (7). This premise has been confirmed in different studies in patients with COVID-19 infection, in whom a coagulation dysfunction is present (6) (8, 9) (10) and is associated with a poor prognosis (9). In a multicenter retrospective cohort study, an elevated D-dimer (> 1000µg/L) was associated with in-hospital death (OR 18.42, 95% CI 2.64-128.55, p=0.0033) (11).

The rate of thromboembolic events in patients with severe COVID-19 infection has been observed to be substantially increased. A study conducted in two hospitals in the Netherlands found a cumulative incidence of thromboembolic events of 31% (95% CI 20-41%) in patients treated on the ICU (12). The incidence of peripheral thromboembolism in patients with severe COVID-19 has been observed to vary between varies from 25 to 69% (13). In a retrospective study in patients with severe COVID-19, a cumulative incidence of peripheral VTE of 69% was found, being 100% in patients with prophylactic anticoagulation in comparison to 56% in patients with therapeutic anticoagulation (14). Regarding DIC, it has been observed that it occurred in 71% of patients who did not survive the infection compared to DIC occurring in <1% of patients who survived the infection (9).

In line with these observations, autopsies of patients with severe hypoxemia confirmed multiple primary pulmonary thrombi and emboli (15). That has also been observed in autopsies at the Charité University Hospital and the University Hospital of Hamburg (approximately 30% pulmonary emboli/thrombosis in COVID-19, unpublished observations). Therefore, in some center's empirical application of systemic anticoagulation as aiming to protect these patients against thromboembolism. Chinese colleagues from the Tongji hospital have retrospectively compared the 28-day mortality between heparin users and non-users in patients with severe COVID-19. Anticoagulant heparin therapy was associated with a better prognosis in patients with severe COVID-19 (16). The 28-day mortality was lower in heparin users compared to non-users in the subgroup of patients with a sepsis-induced coagulopathy score ≥4 (40.0% vs 64.2%, P=0.029), and in the subgroup of patients with a D-dimer >6-fold ULN (32.8% vs 52.4%, P=0.017) (16). These findings point to the importance of a preventive anticoagulation therapy for improving clinical outcomes in specific high-risk subgroups of patients with COVID-19.

Additionally, several studies suggest an association between cardiovascular disease (CVD) and COVID-19, being the presence of these previous conditions also associated with severe disease and mortality (17) (18). A significant proportion of patients with COVID-19 develop acute cardiac injury during the course of the disease. Acute cardiac injury, defined as significant elevation of cardiac troponins, is the most commonly reported cardiac abnormality in COVID-19. It occurs in >10 % of all hospitalized patients. In a meta-analysis of 4 studies with 341 patients, a significant difference of the troponin serum

levels was seen between patients with severe disease in comparison to those with milder disease (delta of 25.64, 95% CI 6.76-44.53, p=0.001) (19).

Although little is known about cardiovascular manifestations in COVID-19, it has become overt that the development of acute cardiac injury significantly worsens the clinical outcome in these patients (20), including the development of thromboembolic events and myocardial infarction.

Taking all these data into account, together with COVID-19-associated systemic inflammation, coagulation activation, hypoxemia and immobilization of the patients upon hospital admission, the actual ESC guidance for the diagnosis and management of cardiovascular disease during the COVID-19 pandemic recommends anticoagulation at standard prophylactic doses for all patients admitted to the hospital with COVID-19 infection (26). Due to the lack of data, it is currently not known whether anticoagulation at therapeutic doses is superior to prophylactic anticoagulation in patients with moderate to severe COVID-19.

Of note, some of the investigational drugs for COVID-19 may have relevant drug-drug interactions with NOACs. In particular, this may be the case for lopinavir/ritonavir due to Cytochrome P450 3A4 (CYP3A4) and/or P-glycoprotein (P-gp) inhibition. In such cases, the bleeding risk may be elevated and NOACs should be avoided. The ESC guidance position points out that severely ill COVID-19 patients should be switched to parenteral anticoagulation, which has no clinically relevant drug-drug interactions with specific COVID-19 therapies. In contrary, no interactions have been described so far for remdesivir and NOACs. Remdesivir has recently received an authorization from the European Commission as first COVID-treatment due to the urgency of the COVID-19 pandemic.

Data from several large prospective randomized clinical trials showed that oral anticoagulation with the direct factor FXa inhibitor, rivaroxaban, prevents not only thromboembolic events in patients with previous VTE, but also reduces major adverse cardiovascular event rates in patients with coronary artery disease. Moreover, factor XIa (FXIa) has become a target for new anticoagulants that appears to be safer than those available now. Phase 2 studies that assess the effect of FXIa inhibitors on clinical outcomes are currently ongoing.

Regarding the lack of data considering anticoagulant therapy in patients with COVID-19, it is crucial to perform prospective randomized trials to substantiate the above-mentioned potential major benefits derived from anticoagulation in these patients. This study is one of the first that compares therapeutic with prophylactic anticoagulation and addresses the role of a NOAC as a potential anticoagulant for COVID-19.

1.1.1. Compound Profile

As part of the prothrombinase complex, FXa directly converts prothrombin to thrombin. Thrombin converts fibrinogen to fibrin and activates platelets leading to clot formation. FXa occupies a critical place in the coagulation cascade since it is at the confluence of both the intrinsic and extrinsic clotting pathways and is the key amplification point for the generation of thrombin. One molecule of FXa is able to generate more than 1,000 molecules of thrombin due to the amplification nature of the coagulation cascade. Selective inhibitors of FXa can terminate the amplified burst of thrombin generation and prevent clot formation.

Rivaroxaban is an oral, direct, FXa inhibitor anticoagulant. Rivaroxaban is rapidly absorbed after oral administration, with peak plasma concentrations occurring approximately 2 to 4 hours post dose. The elimination pathways of rivaroxaban include both hepatic and renal routes. The terminal elimination half-life of rivaroxaban is 5 to 9 hours in healthy young subjects and from 11 to 13 hours in healthy elderly subjects (aged 65 to 83 years). Due to the multiple elimination pathways of rivaroxaban, there are few clinically relevant drug-drug interactions.

1.2. Overall Rationale for the Study

The primary objective is to assess the effect of rivaroxaban compared with standard of care (SOC) with the use of prophylactic low molecular weight heparin (LMWH) or unfractionated heparin (UFH) (see Attachment 1) on D-dimer as a clinical marker for the clinical outcome at day 7 post randomization adjusted for baseline measurement in patients with moderate to severe COVID-19.

The secondary objectives are to assess the efficacy and safety of rivaroxaban compared with standard of care (SOC) with the use of prophylactic low molecular weight heparin (LMWH) or unfractionated

heparin (UFH) in the prevention of thromboembolic events, cardiovascular events, all-cause death or progression to intubation and invasive ventilation up to day 35 in patients with moderate to severe COVID-19.

Previous studies assessed the efficacy and safety of rivaroxaban, compared with SOC in the prevention of symptomatic VTE (lower extremity DVT and non-fatal PE) and VTE-related death (death due to PE or death in which PE cannot be ruled out as the cause) during hospital stay and post-hospital discharge in high-risk medically ill subjects.

The MAGELLAN study failed to demonstrate a positive benefit/risk profile for extended duration thromboprophylaxis in patients hospitalized for acute medical illness, primarily due to excessive bleeding with the anticoagulants (21). However, a subgroup analyses presented as a Forrest plot in the appendix indicated that rivaroxaban 10 mg OD was superior over LMWH/placebo in elderly patients, those suffering from respiratory insufficiency and those affected by infectious or inflammatory diseases, highlighting the patient profile seen in COVID-19. Furthermore, a post-hoc analysis identified elevated D-dimers on admission as a biomarker that indicates patients who would benefit from extended rivaroxaban prophylaxis.

The MARINER study was then designed to improve the overall safety of medically ill patients with rivaroxaban treatment for 45 days post hospital discharge by addressing the bleeding risk observed in the MAGELLAN study. The MARINER patients exhibited a higher VTE-risk than those in MAGELLAN to enhance the overall expected benefit/risk assessment (22). The VTE-risk in the patients from the MARINER Study was more than 2,9% annually according to the IMPROVE Score. The study did not show a benefit in reducing the risk of the composite endpoint (VTE and VTE related death) in acute medically ill patients following hospital discharge. However, when examining VTE only, fewer events were observed in the group treated with rivaroxaban. The incidence of major bleeding in both study arms was low.

According to the actual literature, patients with COVID-19 present a much higher incidence of thrombotic complications than those included in the MARINER study, even with prophylactic anticoagulation (12, 14). Therefore, COVID-PREVENT was designed to determine whether the use of rivaroxaban at a dose of 20mg OD combined with a thromboprophylaxis dose of 10mg OD post discharge reduces fatal or nonfatal thromboembolic and cardiovascular events in comparison to SOC with prophylactic LMWH or UFH in patients with moderate to severe COVID-19.

2. OBJECTIVES AND HYPOTHESIS

2.1 Objectives

Primary Objective

The primary objective is to assess the effect of rivaroxaban compared with standard of care (SOC) with the use of prophylactic low molecular weight heparin (LMWH) or unfractionated heparin (UFH) (see Attachment 1) on D-dimer as a clinical marker for the clinical outcome at day 7 post randomization adjusted for baseline measurement in patients with moderate to severe COVID-19.

The co-primary objective is to evaluate the impact of rivaroxaban compared with standard of care (SOC) with the use of prophylactic low molecular weight heparin (LMWH) or unfractionated heparin (UFH) on a seven-category ordinal scale recommended by the WHO as a measure of clinical benefit at day 7 post randomization adjusted for baseline score in patients with moderate to severe COVID-19.

Primary Endpoint

- D-dimer at day 7 post randomization

Co-primary Endpoint

- Seven-category ordinal scale recommended by the WHO at day 7 post randomization (Attachment 2)

Secondary Objectives

The secondary objectives are to assess the efficacy and safety of rivaroxaban compared with standard of care (SOC) with the use of prophylactic low molecular weight heparin (LMWH) or unfractionated heparin (UFH) in the prevention of thromboembolic events, cardiovascular events, all-cause death or progression to intubation and invasive ventilation up to day 35 in patients with moderate to severe COVID-19.

Secondary combined Endpoint Time to first event of either of

- Venous thromboembolism (VTE) (deep venous thrombosis (DVT) and/or fatal or non-fatal pulmonary embolism (PE))
- Arterial thromboembolism
- New myocardial infarction (MI)
- Non-hemorrhagic stroke
- All-cause death
- Progression to intubation and invasive ventilation

In addition, the components VTE, arterial thromboembolism, MI, non-hemorrhagic stroke and all-cause-death of the secondary endpoint will be additionally evaluated, if sufficient numbers of events are observed.

Furthermore, all single components of the secondary composite endpoint s will be additionally evaluated separately, if sufficient numbers of events are observed.

Safety Objectives

The safety objectives are to compare rivaroxaban with SOC with prophylactic LMWH or UFH in bleeding outcomes up to day 35 in patients with moderate to severe COVID-19.

Primary Safety Endpoints:

- Fatal and non-fatal major bleeding using the International Society on Thrombosis and Haemostasis (ISTH) bleeding criteria

Secondary Safety Endpoints:

- Clinically relevant non-major bleeding
- Non-major bleeding that lead to study-drug interruption for more than 7 days

Other endpoints of interest:

- Length of hospital stay
- Time to intubation
- Re-hospitalization due to heart failure
- Re-hospitalization due to any other reason
- Effects on coagulation parameters for thrombosis (TAT, PAI-1, PF-4, TF, TF-activity)
- Effects on inflammatory and fibrotic parameters (interleukines, interferons, growth factors)
- Change of N-terminal prohormone brain natriuretic peptide (NT-pro-BNP)
- Unscheduled outpatient visits

2.2 Hypotheses

The primary hypothesis is that rivaroxaban but not SOC with prophylactic LMWH or UFH is effective in the reducing D-dimer at day 7 post randomization compared to baseline in patients with moderate to severe COVID-19. The co-primary hypothesis is that rivaroxaban compared to SOC with prophylactic

LMWH or UFH is superior to improve the patients' categories on a seven-category ordinal scale for patients with respiratory infections at day 7 post randomization.

The secondary hypothesis is that therapeutic anticoagulation with rivaroxaban for at least 7 days, followed by prophylactic anticoagulation with rivaroxaban for 28 days is superior to SOC with prophylactic LMWH or UFH in the prevention of the composite of VTE (DVT and/or fatal or non-fatal PE), arterial thromboembolism, new MI, non-hemorrhagic stroke, all-cause death and progression to intubation and invasive ventilation up to day 35 post randomization in patients with moderate to severe COVID-19.

3. STUDY DESIGN AND RATIONALE

3.1 Overview Study Design

This is a multicenter, prospective, randomized, event-driven phase II study designed to assess the effect of therapeutic anticoagulation with high dose rivaroxaban intended for at least 7 days compared with SOC with prophylactic LMWH or UFH on the clinical prognosis marker D-dimer at day 7 post randomization adjusted for baseline measurement in patients with moderate to severe COVID-19. Moreover, this study investigates the effect of therapeutic anticoagulation with rivaroxaban compared with SOC with prophylactic LMWH or UFH on a seven-category ordinal scale for patients with respiratory infections at day 7 post randomization adjusted for baseline score.

Secondarily, this trial is designed to evaluate therapeutic anticoagulation with rivaroxaban for at least 7 days followed by thromboprophylaxis with rivaroxaban for 28 days, as compared with SOC with prophylactic LMWH or UFH in the prevention of the composite of VTE (DVT and/or fatal or non-fatal PE), arterial thromboembolism, new MI, non-hemorrhagic stroke, all-cause death and progression to intubation and invasive ventilation up to day 35 post randomization in patients with moderate to severe COVID-19. The study consists of a screening phase, a therapeutic treatment phase of at least 7 days and a thromboprophylaxis phase up to day 35 post randomization. The physicians of the respective trial site decide which heparin, of the heparins approved in Germany, the patients assigned to treatment group "SOC with prophylactic UFH or LMWH" will receive.

The subject population comprises men and women aged 18 years and older who have been tested positive for SARS-CoV-2 and who have moderate to severe COVID-19.

In order to be eligible, all patients must exhibit at least one of the following conditions:

- D-dimer elevation > 1.5 ULN (using age-adjusted cut-offs) and/or
- Cardiac injury reflected by an elevation in hs-cTnT > 2.0 ULN and one of the following conditions:
    - Known CAD
    - Known diabetes mellitus
    - Active smoking

The inclusion of D-dimer negative patients with cardiac injury reflected by an elevation in hs-cTnT > 2.0 ULN and one of the following conditions, known CAD or known diabetes mellitus or active smoking, will be limited to 15 % of the patients included in the study.

85% or more of the patients enrolled should be enrolled due to increased D-dimer defined as:

- D-Dimer elevation above 750 µg/L in patients 50 years of age or younger.
- In patients older than 50 years an age-adjusted cut-off will be calculated using the following formula: 15 x Age µg/L.

Any patient with a medical condition that requires use of any therapeutic parenteral or therapeutic oral dose anticoagulation (e.g. atrial fibrillation) is not eligible for participation. Moreover, patients at particularly increased risk of bleeding and those using medications that might interact with the study drug will be excluded.

Subjects with moderate to severe respiratory symptoms, tested positive for SARS-CoV-2, can be enrolled into the study, if the subjects meet all of the inclusion and none of the exclusion criteria. The

subjects will be randomly assigned to receive treatment with rivaroxaban or SOC with prophylactic LMWH or UFH.

Randomization can occur during the hospital stay or out-of-hospital, if admission to the hospital is not necessary, whatever the treating physician judges. Randomization should not occur later than 3 days after screening. Subjects will be randomized by strata including site, gender (male, female, diverse), age (< 65 versus ≥ 65 years), kidney function (eGFR ≥ 30 mL/min/1.73m^2 and < 50 mL/min/1.73m^2 versus eGFR ≥ 50 mL/min/1.73m^2), history of CAD or heart failure (yes/no), oxygen demand on admission to the hospital (supplementary oxygen required versus not required) and setting (outpatients versus hospitalized patients).

After randomization, subjects will receive treatment with rivaroxaban 20 mg (15 mg for subjects with an eGFR ≥30 mL/min/1.73m^2 and <50 mL/min/1.73m^2) once daily (OD) for at least 7 days or SOC with prophylactic LMWH or UFH according to the local standards and choice of the treating physician.

In case of hospitalization for more than 7 days, the therapeutic treatment with rivaroxaban will be continued for the duration of the hospital stay until discharge. After at least 7 days of therapeutic treatment with rivaroxaban or after hospital discharge, whatever is the event that occurs later, the study dose of rivaroxaban will be adjusted as follows: Patients randomized to rivaroxaban 20mg OD will reduce daily dosage to 10 mg OD, provided that they were not diagnosed with a condition requiring continued therapeutic anticoagulation. Thromboprophylaxis therapy will be given for 28 days up to day 35 post randomization or even longer. If the patient cannot be discharged from the hospital prior to day 35 post randomization, the thromboprophylaxis phase will also start upon hospital discharge, but is then shorter than 28 days, because the study ends latest at day 60 post randomization. The complete end of study (EOS) visit will be done via phone call 60 days post randomization. No study medication will be given thereafter.

After the study, the patients continue to receive medical therapy according to local practice.

If the clinical condition of a hospitalized patient deteriorates and admission to ICU with possible invasive ventilation will become necessary, treatment with rivaroxaban should be discontinued for the ICU period and the patient switched to parental or subcutaneous anticoagulation following best local practice. After clinical improvement and discontinuation of invasive ventilation, the anticoagulation mentioned above can be replaced by study drug rivaroxaban 20 mg OD (15 mg for subjects with an eGFR ≥30 mL/min/1.73m^2 and <50 mL/min/1.73m^2) at the discretion of the clinically responsible physician.

Patients randomized to the SOC treatment arm will receive SOC during the time of hospitalization or for at least 7 days, whatever is later. SOC constitutes of prophylactic doses of LMWH or UFH, according to the local practice and decision of the physicians of the respective trial site. Study visits are terminated 7, 35, and 60 days post randomization in all patients. The latter will be done via phone call if applicable. For the hospitalized patients who are discharged later than 10 days post randomization, an additional discharge visit will be done at the day of discharge (-2 days).

If the treating physician switches a patient in the SOC treatment group from prophylactic to therapeutic anticoagulation, a medically evidence-based reason must be given. All changes of anticoagulation must be properly documented in the eCRF.

The first dose of the study drug should be administered as soon as possible and no later than the day after the subject is randomized. Subjects and medical personal in charge of the administration of the drugs will be instructed how to administer the drug.

If a subject has a suspected efficacy or bleeding outcome event during the study, the treating physician should exercise clinical judgment and follow established guidelines.

The primary objective is to assess the effect of rivaroxaban compared with standard of care (SOC) with the use of prophylactic low molecular weight heparin (LMWH) or unfractionated heparin (UFH) on D-dimer as a clinical marker for the clinical outcome at day 7 post randomization adjusted for baseline measurement in patients with moderate to severe COVID-19.

The co-primary objective is to evaluate the impact of rivaroxaban compared with standard of care (SOC) with the use of prophylactic low molecular weight heparin (LMWH) or unfractionated heparin (UFH) on a seven-category ordinal scale recommended by the WHO (Attachment 2) as a measure of clinical benefit at day 7 post randomization adjusted for baseline score in patients with moderate to severe COVID-19 (23-25).

The secondary objectives are to assess the efficacy and safety of rivaroxaban compared with standard of care (SOC) with the use of prophylactic low molecular weight heparin (LMWH) or unfractionated heparin (UFH) in the prevention of thromboembolic events, cardiovascular events, all-cause death or progression to intubation and invasive ventilation up to day 35 in patients with moderate to severe COVID-19.

The principal safety endpoint is fatal or non-fatal major bleeding as defined according to the ISTH classification.

Independent of the antithrombotic therapy, it should be stressed that all study patients will receive SOC with regard to COVID-19 specific therapies, such as anti-viral drugs, e.g. remdesivir or others, if clinically necessary.

An Executive Committee (EC), a Data Safety Monitoring Board (DSMB) and a Clinical Event Committee (CEC) will be commissioned for this study.

Figure 1 outlines the study design.

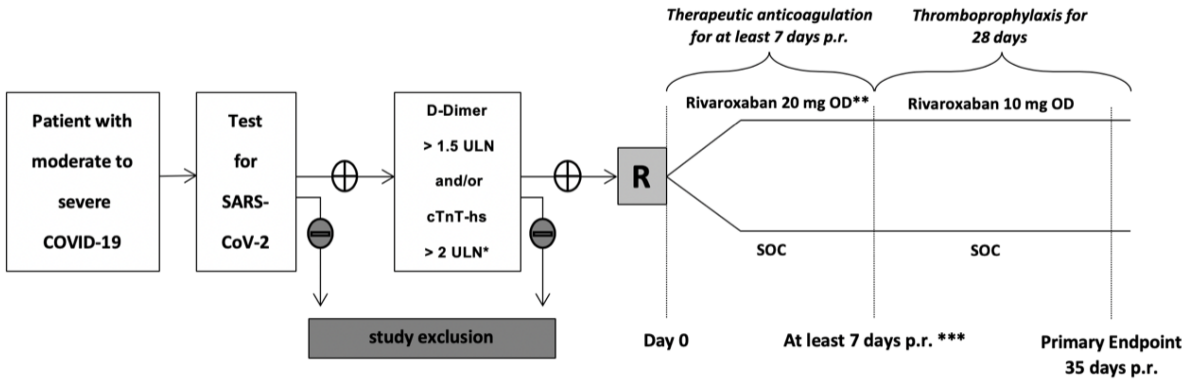

Figure 1: Study design

SOC = standard of care with prophylactic LMWH or UFH during hospitalization or for at least 7 days, whatever is later

p.r. = post randomization

*= and at least one of the following: known CAD, diabetes mellitus or active smoking

**= if hospitalized for more than 7 days extension of therapeutic anticoagulation is allowed up to hospital discharge; in critical cases in need of intubation and invasive ventilation requiring ICU stay, rivaroxaban treatment will be switched to therapeutic anticoagulation with LMWH or UFH (if treating physicians consider the bleeding risk not as very high in patients with critical COVID-19), in case the bleeding risk is considered higher the patients would be switched to prophylactic anticoagulation

***= switch from therapeutic to prophylactic anticoagulation can occur later than 7 days p.r., e.g. if the patient is discharged later than 7 days p.r. and, therefore, receives therapeutic anticoagulation until hospital discharge

3.2. Study Design Rationale

Study Population

Patients infected with SARS-CoV-2 that suffer from moderate to severe COVID-19 (Attachment 5). It has been shown that these patients have a much higher risk of development of thromboembolic events (12) .However, until now no randomized clinical trial has been done to evaluate the efficacy and safety of therapeutic anticoagulation followed by thromboprophylaxis in comparison to SOC with prophylactic anticoagulation (LMWH or UFH) in moderate to severe COVID-19.

Randomization

Randomization and stratification will be used to minimize bias in the assignment of subjects to treatment groups, to increase the likelihood that known and unknown subject attributes (e.g., demographic and baseline characteristics) are evenly balanced across treatment groups, and to enhance the validity of

statistical comparisons across treatment groups. Subjects will be randomized by strata including site, gender (male, female, diverse), age (< 65 versus ≥ 65 years), kidney function (eGFR ≥ 30 mL/min/1.73m^2 and < 50 mL/min/1.73m^2 versus eGFR ≥ 50 mL/min/1.73m^2), history of CAD or heart failure (yes/no), oxygen demand on admission to the hospital (supplementary oxygen required versus not required) and setting (outpatients versus hospitalized patients).

Comparator Selection

The control group in the COVID-PREVENT study will receive SOC with prophylactic LMWH or UFH. The decision was made based on consideration of the literature and especially the current ESC guidance for the diagnosis and management of cardiovascular disease during the COVID-19 pandemic. (26) The physicians of the respective trial site decide which heparin, of the heparins approved in Germany, the patients assigned to treatment group "SOC with prophylactic UFH or LMWH" will receive.

The optimal dosing of anticoagulation in patients with moderate to severe COVID-19 is still unknown as is also the use of thromboprophylaxis after discharge (26). Based on this, SOC with prophylactic LMWH or UFH has been selected as the comparator to allow for the evaluation of benefit/risk of rivaroxaban as initial therapeutic anticoagulant and as thromboprophylaxis agent in moderate to severe COVID-19 cases.

Should the subject develop during the study any condition, which in the investigator’s judgment requires long-term therapeutic anticoagulation, or fibrinolysis, the subject will have study treatment either temporarily interrupted or permanently discontinued and will be managed as deemed appropriate by the treating physician. The subject will be asked to continue in the study to be followed for efficacy and safety outcomes.

Dose Selection in the treatment arm receiving rivaroxaban

The selection of a dose of 20 mg daily of rivaroxaban is based primarily on the observational studies that show that in patients with moderate to severe COVID-19, despite prophylactic anticoagulation the incidence of VTE remains high (12).

The decision to give a dose of 10 mg for 28 days after discharge is based on studies that showed that the rate of VTE events was lower in patients treated with rivaroxaban in comparison to placebo in acutely medically ill patients after being discharged while the major bleeding incidence remained low (22).

Subjects with eGFR ≥50 mL/min/1.73m^2 will be randomly assigned to receive rivaroxaban 20 mg OD or SOC. A dose adjustment of rivaroxaban to 15 mg OD will be implemented for subjects with an eGFR ≥30 mL/min/1.73m^2 and <50 mL/min/1.73m^2 according to the SmPC. For the out of hospital treatment phase a daily dose of rivaroxaban of 10 mg will be used for all patients discharged with an eGFR > 30 ml/min.

3.3. Committees

3.3.1. Executive Committee (EC)

The EC consists of members of the academic leadership of the study and can give suggestions to the sponsor. Ad hoc members may be appointed as necessary. The EC has overall responsibility for the design of the study. The EC will monitor overall safety during the study and will receive any recommendations from the DSMB regarding possible additional analyses or modifications to the study and decide whether to accept them. The EC will oversee the implementation of any modifications to the study and publication of the results.

3.3.2. Independent Data Safety Monitoring Board (DSMB)

The DSMB will be independent of the sponsor, coordinating or principal Investigator(s), site investigators, Executive Committee or Clinical Events Committee (CEC) membership, or any other capacity related to study operations.

An independent DSMB will be established to monitor the progress of the study and to ensure that the safety of subjects enrolled in the study is not compromised. The sponsor will update the DSMB on all reported SAEs, outcome events, and safety information on a near real time basis. The DSMB will consider all information regarding benefits and risks and will judge the regular information on efficacy and safety data in the context of newly emerging evidence on optimal management of patients with

COVID-19. The DSMB will include, but is not limited to, a clinical chairman, physician(s) experienced in clinical trials, but not participating in this study. Details of the composition, roles, responsibilities, and processes of the DSMB will be documented in its charter. The DSMB will review results of the planned interim analysis and make a recommendation whether the study should be continued as planned, modified, or terminated prematurely due to futility or safety. Any recommendations from the DSMB will be made available to the EC.

If the DSMB makes recommendations on the conduct of the trial that are considered to have significant bearing on the benefit-risk of the trial, the sponsor will take appropriate measures and communicate them to the competent authorities, ethics committees and investigators as applicable. The decision for appropriate measures (e.g. define an amendment; early termination) will be made in consultation with the sponsor. The final decision will be made by the sponsor.

Composition of the DSMB:

The number of DSMB members consists of –at least - three members including:

• 1 expert in the clinical aspects of the disease/patient population being studied, i.e. clinical pharmacologist;

• 1 biostatistician;

• 1 investigator with expertise in current clinical trials conduct and methodology (an expert in anti-infectives, pulmonology, and/or cardiology).

Ad-hoc specialists may be invited to participate as non-voting members at any time if additional expertise is desired.

Operation of the DSMB:

The frequency of DSMB meetings depends on several factors including the rate of enrolment, safety issues or unanticipated adverse events and availability of data. The Chair of the DSMB is responsible for defining meeting schedules, the sponsor of the trial is responsible for convening meetings, selecting a venue when the meeting is not convened by teleconference or web meeting, and coordinating the distribution of meeting materials to DSMB members and other meeting participants. The agenda for each meeting is generally developed jointly by the sponsor of the trial and the DSMB Chair.

The first DSMB meeting should be before the first visit of the first patient. Another DSMB meeting should be held early before the enrollment of 50 patients. At the first meeting, the DSMB should discuss and decide on the DSMB charter which includes triggers set for data review or analyses, definition of a quorum, nomination of the DSMB Chair, and guidelines for monitoring the study. The DSMB members should have the opportunity to review the study protocol before they decide to become a member.

Once the study is implemented, the DSMB should convene as often as necessary, but at least once every 2 months, to examine the data, review study progress, and discuss other factors (internal or external to the study) that might impact continuation of the study as designed. The DSMB will alter the frequency of this review process, if necessary. On the decision of DSMB members the meetings can be convened by teleconference or web meeting.

A DSMB meeting may be requested by DSMB members, the sponsor, or Institutional Review Board (IRB) at any time to discuss safety concerns. Decisions to hold ad-hoc meetings will be made by the DSMB Chair.

In the event a DSMB member cannot attend a meeting, he/she may receive a copy of the closed session DSMB report and either participate by conference call or web meeting or provide written comments to the DSMB Chair for consideration at the meeting. Replacement of a DSMB member by another person is impossible.

The recommended meeting format consists of Open Sessions[1] and Closed Sessions[2]. Further details regarding the DSMB can be found in the DSMB charter.

[1] Open Sessions: DSMB members and external experts

[2] Closed Sessions: Only DSMB members

3.3.3. Clinical Events Committee (CEC)

The CEC is comprised of independent, board-eligible or board-certified specialist physicians as appropriate and necessary. Committee members do not directly enroll subjects in the study, and do not have direct operational responsibilities for the conduct of the study. Members will review all suspected outcome events as described below that occurred post-randomization as they become available and classify in a consistent and unbiased manner according to definitions in the CEC charter (see Attachment 4) and make recommendations to the sponsor.

4. SUBJECT POPULATION

Screening may begin once the informed consent is obtained. If a subject's status changes (including laboratory results or receipt of additional medical records) after screening but before randomization such that he or she no longer meets all eligibility criteria, then the subject should not be randomized.

The inclusion and exclusion criteria for enrolling subjects in this study are described in the following 2 subsections. If there is a question about the inclusion or exclusion criteria below, the investigator should consult with the appropriate sponsor representative before enrolling a subject in the study.

4.1. Inclusion Criteria

Each potential subject must satisfy all of the following criteria to be enrolled in the study.

1. Subject must be willing, understanding and able to provide written informed consent
2. Subject must be a man or a woman with age $\geq$ 18 years at screening
3. Subject must have an active moderate to severe COVID-19 confirmed by
    a. A positive SARS-CoV-2 PCR test in the last 14 days
4. At least one of the following features should be present
    a. D-Dimer elevation > 1.5 ULN (age adjusted cut-offs) AND/OR
    b. Cardiac injury reflected by an elevation in hs-cTnT > 2.0 ULN AND at least one of the following conditions:
        i. Known CAD
        ii. Known diabetes mellitus
        iii. Active smoking
5. A woman of childbearing potential must have a negative serum or urine pregnancy test before randomization occurs. Before randomization, a woman must be either:
    a. Postmenopausal, defined as >45 years of age with amenorrhea for at least 18 months,
    b. If menstruating:
        i. If heterosexually active, practicing a highly effective method of birth control with a failure rate less than 1% per year, including hormonal prescription oral contraceptives, contraceptive injections, contraceptive patch or intrauterine device, or male partner sterilization, consistent with local regulations regarding use of birth control methods for subjects participating in clinical studies, for the duration of their participation in the study, or
        ii. Surgically sterile (have had a hysterectomy or bilateral oophorectomy, tubal ligation, or otherwise be incapable of pregnancy), or
        iii. Not heterosexually active

4.2. Exclusion Criteria

Any potential subject who meets any of the following criteria will be excluded from participating in the study.

1. Subject has a very high bleeding risk: Any condition that, in the opinion of the investigator, contraindicates anticoagulant therapy or would have an unacceptable risk of bleeding, such as, but not limited to, the following:

    a. Any bleeding (defined as bleeding requiring hospitalization, transfusion, surgical intervention, invasive procedures, occurring in a critical anatomical site, or causing disability) within 1 months prior to randomization or occurring during index hospitalization.

   b. Major surgery, biopsy of a parenchymal organ, ophthalmic surgery (excluding cataract surgery), or serious trauma (including head trauma) within 4 weeks before randomization.
   c. A history of hemorrhagic stroke or any intracranial bleeding at any time in the past, evidence of primary intracranial hemorrhage on CT or magnetic resonance imaging scan of the brain, or clinical presentation consistent with intracranial hemorrhage. This applies as well to subjects hospitalized for ischemic stroke upon randomization.
   d. Subject has a history of or current intracranial neoplasm (benign or malignant), cerebral metastases, arteriovenous (AV) malformation, or aneurysm.
   e. Active gastroduodenal ulcer, defined as diagnosed within 1 months or currently symptomatic or known AV malformations of the gastrointestinal tract.
   f. Platelet count <90,000/µl at screening.
   g. Patients with the diagnosis of bronchiectasis, that due to the investigator judgement are at an increased bleeding risk.
2. Subject has any of the following diseases in the medical history:
   a. Active cancer (excluding non-melanoma skin cancer) defined as cancer not in remission or requiring active chemotherapy or adjunctive therapies such as immunotherapy or radiotherapy. Chronic hormonal therapy (e.g. tamoxifen, anastrozole, leuprolide acetate) for cancer in remission is allowed.
   b. Any medical condition (e.g. atrial fibrillation) that requires use of any therapeutic parenteral or oral anticoagulant(s) (e.g. warfarin sodium or other vitamin K antagonists, Factor IIa or FXa inhibitors, fibrinolytics) concomitantly with study medication. In case of “off label” use of a NOAC, the patient can be included in the study if the NOAC was stopped for 24 hours or more.
   c. Subject has known allergies, hypersensitivity, or intolerance to rivaroxaban or any of its excipients.
   d. Baseline eGFR <30 mL/min/1.73m^2 calculated using CKD-EPI formula provided in Attachment 3.
   e. Known significant liver disease (e.g. acute hepatitis, chronic active hepatitis, cirrhosis) which is associated with coagulopathy or moderate or severe hepatic impairment.
   f. Known HIV infection.
3. Subject has undergone any of the following procedures or received any of the following drugs:
   a. Received fibrinolysis during index hospitalization.
   b. Use of antiplatelet therapy with prasugrel or ticagrelor up to 7 days prior to randomization. Other P2Y12 antagonists can be given. However, the use of concomitant antiplatelet therapy should be carefully considered. ASS > 100 mg/d and continuous NSAIDs should be avoided.
   c. Use of dual antiplatelet therapy, such as aspirin plus clopidogrel during the study.
4. Subjects with acute critical illness that leads to an altered mental status affecting the ability to consent (i.e. hemodynamically unstable, patients with immediate indication for ICU admission)
5. Subject is a woman who is pregnant or breast-feeding.
6. Known intolerance or history of hypersensitivity to the active substance or to any of the excipients of the Investigational Medicinal Product (IMP)
7. Other Contraindications for the use of the IMPs according to the SmPCs
8. Subjects who are legally detained in an official institution.
9. Subjects who may be dependent on the sponsor, the investigator or the trial sites, are not eligible to enter the trial.

10. Subjects participating in another clinical trial of an investigational medicinal product or device.

NOTE: Investigators should ensure that all study enrollment criteria have been met at screening. If a subject's status changes (including laboratory results or receipt of additional medical records) after screening but before randomization such that he or she no longer meets all eligibility criteria, the subject should not be randomized. But if the subject has been randomized, he/she should not be excluded from participation in the study and must be followed until the EOS visit.

4.3. Prohibitions and Restrictions

Potential subjects must be willing and able to adhere to the following prohibitions and restrictions to be eligible for participation. If subjects require or take prohibited medications during the study as outlined below, they must either temporarily interrupt or permanently discontinue the study drug, as appropriate for the duration of the therapy with the prohibited medication:

- Combined P-glycoprotein 1 (P-gp) and strong cytochrome P540 3A4 (CYP3A4) inhibitors (such as ketoconazole, telithromycin or certain protease inhibitors such as but not limited to ritonavir) use within 4 days before randomization.
- Combined P-gp and strong CYP3A4 inducers (such as rifampin/rifampicin, rifabutin, rifapentine, phenytoin, phenobarbital, carbamazepine, or St. John's Wort) use within 4 days before randomization.
- If the patient needs treatment with a protease inhibitor such as ritonavir during the in-hospital treatment phase, he/she needs to stop the therapy with rivaroxaban. The subject will then receive therapeutic anticoagulation with LMWH or UFH instead of rivaroxaban. 4 days after the therapy with ritonavir has been discontinued, treatment with rivaroxaban can be restarted. Thus, the therapeutic treatment with LMWH or UFH will be switched to rivaroxaban again.
- Study drug has also to be temporarily interrupted in critical patients who need mechanical ventilation or develop shock or organ failure requiring ICU care. Then, the rivaroxaban treatment will be interrupted and therapeutic anticoagulation with LMWH or UFH will be started if treating physicians consider the bleeding risk not as very high. In case the bleeding risk is considered higher the patients would be switched to prophylactic anticoagulation.
- Anticoagulation with warfarin sodium or other vitamin K antagonists is prohibited during the study. Factor IIa inhibitors or other FXa inhibitors than rivaroxaban must not be used as concomitant therapy during the study. If a patient doesn't have an indication for anticoagulation but received a NOAC with an "off label" use, the NOAC has to be stopped 24 hours prior to randomization.

5. TREATMENT ALLOCATION AND BLINDING

Treatment Allocation

Procedures for Randomization and Stratification

This trial is an open label study. Central randomization will be implemented in this study. Subjects will be randomly assigned to 1 of 2 treatment groups (rivaroxaban or SOC with prophylactic UFH or LMWH) in a 1:1 fashion based on a computer-generated randomization schedule prepared before the study starts under the supervision of the sponsor. Randomization will follow a dynamic randomization algorithm with residual randomness designed to minimize imbalance in terms of variance between treatments taking the following stratification factors into account: site, gender (male, female, diverse), age (< 65 versus ≥ 65 years), kidney function (eGFR ≥ 30 mL/min/1.73m^2 and < 50 mL/min/1.73m^2 versus eGFR ≥ 50 mL/min/1.73m^2), history of CAD or heart failure (yes/no), oxygen demand on admission to the hospital (supplementary oxygen required versus not required) and setting (outpatients versus hospitalized patients). The computer system will dictate the treatment assignment for the subject. The requestor must use his or her own user identification and personal identification number when contacting the system and will then give the relevant subject details to uniquely identify the subject.

6. DOSAGE AND ADMINISTRATION

Treatment groups in this study are rivaroxaban and SOC with prophylactic LMWH or UFH. This study is open label; thus physicians and patients are aware of the anticoagulants applied. Subjects will be randomly assigned in a 1:1 ratio to receive rivaroxaban 20 mg OD (15 mg OD in subjects eGFR≥30 mL/min/1.73m^2 and <50 mL/min/1.73m^2) or SOC with prophylactic LMWH or UFH. The physicians of the respective trial site decide which heparin, of the heparins approved in Germany, the patients assigned to treatment group "SOC with prophylactic UFH or LMWH" will receive. Dosage specifications are listed in Attachment 1.

Randomization will be stratified according to site, gender (male, female, diverse), age (< 65 versus ≥ 65 years), eGFR (subjects with eGFR ≥ 30 mL/min/1.73m^2 and < 50 mL/min/1.73m^2 versus subjects with eGFR ≥ 50 mL/min/1.73m^2), history of CAD or heart failure (yes/no), oxygen demand on admission to the hospital (supplementary oxygen required versus not required) and setting (outpatients versus hospitalized patients).

The dose of study drug will be adjusted in the event of change in eGFR during the course of the study. The first dose of study drug should be administered as soon as possible after randomization.

The date and time of the first dose of study drug and the last dose of prophylactic LMWH or UFH should be recorded as accurately as possible. The first dose of study drug should not be delayed allowing administration under supervision. All subjects should take study drug (rivaroxaban) daily with food at approximately the same time each day. A missed dose should be taken as soon as possible (up to 8 hours prior to the next scheduled dose), and the next scheduled dose should be taken at the regular time.

Throughout the study, study drug will be dispensed at appropriate intervals (see the TIME AND EVENTS SCHEDULE) to ensure that subjects have adequate quantities of study drug between study visits. After at least 7 days of therapeutic treatment with rivaroxaban or after hospital discharge, whatever is the event that occurs later, the study dose of rivaroxaban will be adjusted as follows: Patients randomized to rivaroxaban 20 mg OD (15 mg OD in case of renal impairment) will reduce daily dosage to 10 mg OD, provided that they were not diagnosed with a condition requiring continued therapeutic anticoagulation. Thromboprophylaxis in the rivaroxaban arm will be given for the duration of 28 days, but not longer than 60 days post randomization.

If a patient that at the time of randomization into the rivaroxaban arm has been an out-patient is hospitalized a therapeutic anticoagulation with rivaroxaban 20 mg OD (15 mg OD in subjects eGFR≥30 mL/min/1.73m^2 and <50 mL/min/1.73m^2) should be performed during hospital stay.

If the subject is hospitalized for more than 60 days post randomization, anticoagulation will be given according to the local standards. However, chronic therapy with the study drug is not being tested. For these reasons, rivaroxaban will not be provided to the subject after the subject completes the EOT visit 60 post randomization unless required by local regulations. It will only be after data from the entire study is analyzed that it will be determined if the population at risk will benefit from treatment.

Interruption of Study Drug

Study drug may be interrupted temporarily as necessary for invasive procedures or as medically needed (e.g., in the setting of a bleeding event or a required prohibited therapy). If a subject is hospitalized for any reason other than a VTE-related event or bleeding, study drug should be continued during hospitalization unless the treating physician feels that anticoagulation, temporary interruption or permanent discontinuation of study drug is clinically warranted. Subjects may be placed on appropriate anticoagulation at the discretion of the treating physician. In that case, study drug may be restarted upon discharge at the discretion of the investigator. These interruptions will be recorded on the electronic case report form (eCRF). Intentional and unintentional stopping of study drug by the subject will be documented. Any study drug interruption will be recorded in the eCRF.

7. TREATMENT COMPLIANCE

Subjects will return empty study drug containers and unused study drug when a new supply of study drug is to be received. The last dose of study drug should not be taken later than 60 days post randomization. Subjects will also return their study drug containers and unused study drug at the EOT visit. Study drug accountability will be performed as indicated in the TIME AND EVENTS SCHEDULE.

Subjects should report any intentional or unintentional interruption or missed doses to the study site personnel at each visit.

8. PRESTUDY AND CONCOMITANT THERAPY

For each subject, the drug identity and dose of the following relevant medications taken during the study will be recorded on the appropriate page of the eCRF: antiplatelet (including non-steroidal anti-inflammatory drugs) and anticoagulant medications, statins, and medications relevant to adverse events. Only the drug identity should be recorded for proton pump inhibitors, glucocorticoids, antiviral drugs and hormonal therapies. Prophylactic treatment with LMWH or UFH during the screening phase and complete index hospitalization must be reported as accurately as possible.

Allowed Therapy

All decisions regarding concomitant medications are left to the treating physician unless otherwise required by the protocol.

Prohibited Therapy

The following concomitant medications are not allowed while enrolled in the study. Please also refer to the SmPC of the respective study drug.

- prasugrel
- ticagrelor
- phenprocoumon, warfarin sodium or other vitamin K antagonists
- apixaban within 24 hours prior to randomization
- dabigatranetexilat within 24 hours prior to randomization
- edoxaban within 24 hours prior to randomization
- P-glycoprotein 1 (P-gp)/ cytochrome P540 3A4 (CYP3A4) inhibitors, such as ketoconazol, telithromycin, used within 4 days before randomization and during the study
- P-gp/CYP3A4 inducer, such as rifampin/rifampicin, rifabutin, rifapentine, phenytoin, phenobarbital, carbamazepine or St. John’s Wort, use within 4 days before randomization or during the study
- protease inhibitors, such as ritonavir, use within 4 days before randomization or during the study

9. STUDY EVALUATIONS

9.1. Study Procedures

9.1.1. Overview

The TIME AND EVENTS SCHEDULE in the synopsis summarizes the frequency and timing of procedures applicable to this study.

9.1.2. Screening Phase

Screening may begin any time after diagnosis of moderate to severe COVID-19, and once informed consent is obtained. The subject might be eligible for the study, if the SARS-CoV-2 PCR test had been positive within 14 days prior to randomization. There is no need to repeat the SARS-CoV-2 PCR test in the screening period if so. Subjects with SARS-CoV-2 infection requiring a D-dimer elevation > 1.5 ULN (age-adjusted cut-off) and/or cardiac injury reflected by an elevation of hs-cTnT > 2.0 ULN (and one of the following criteria: known CAD, diabetes mellitus or active smoking) before randomization are eligible for the study. For those subjects, if the D-dimer or hs-cTnT was not ordered as part of the standard care by the treating physician during the index event, a screening sample may be obtained by site staff after obtaining informed consent. No other screening procedures must be performed until the informed consent is obtained.

In a structured interview the patient’s medical history, demographics, concomitant medication and information about inclusion and exclusion criteria will be enquired. A physical examination will be

performed and the patient's vitals including pulse, blood pressure and oxygen saturation will be documented.

Eligibility based on laboratory results will be determined using local laboratory results for the inclusion and exclusion clinical laboratory parameters, and the latest one will also serve as baseline if there are multiple laboratory values. Laboratory screening assessments that are part of the standard care performed by the investigator do not need to be repeated if performed within the time frame required by the protocol.

A local D-dimer and hs-cTnT value should be obtained on all subjects; values taken up to 3 days prior to screening day can be used. If more than one D-dimer value is available, the value obtained closest to the screening day should be used. Moreover, blood values for safety considerations will be obtained (hemoglobin/platelet count, creatinine, AST, ALT, INR, aPTT, hs-CRP, NT-pro-BNP).

The eGFR will be calculated according to the CKD-EPI formula (provided in Attachment 3).

An ECG will be performed on the screening visit and blood for further safety assessment will be drawn and sent to the local laboratory.

In women with child-bearing potential a urine or blood pregnancy test will be performed before.

9.1.3. Treatment Phase

Randomization/Day 0

If the subject meets all of the inclusion and none of the exclusion criteria, he or she is eligible to be randomly assigned to receive rivaroxaban or SOC with prophylactic LMWH or UFH at the Day 0 visit.

Randomization should occur no more than 3 days after the screening.

Also, before randomization occurs, site staff should review eligibility criteria, including concomitant medications administered during the index event. Attention should be paid to ensure that that prohibited medications were not administered. The patient will be initially assessed according to the seven-category ordinal scale, as recommended by the WHO (Attachment 2) in order to be able to evaluate the clinical improvement during the course of the disease (23-25). The treating physician will be informed about the patient participation in the study.

After randomization, the first dose of study drug should be started on the next day or on the same day. The time of the first dose should be recorded as accurately as possible. The first dose does not need to be delayed if the last administration of prophylactic LMWH or UFH was longer than 6 hours ago. The administration date and time of the first dose of study drug will be recorded in the source document and eCRF.

During the hospital stay the subject and the hospital personal in charge of administration of drugs should be instructed on how to take the drug. They will be instructed to take the drug (rivaroxaban 20 mg OD, 15 mg OD if eGFR ≥30 mL/min/1.73m^2 and <50 mL/min/1.73m^2 if randomized to the rivaroxaban arm) through discharge or until attending physician considers an adjustment in the anticoagulation needs to be made.

Patients in the out of hospital setting will receive the drug (rivaroxaban 20 mg OD, 15 mg OD if eGFR ≥30 mL/min/1.73m^2 and <50 mL/min/1.73m^2 if randomized to the rivaroxaban arm) for 7 days post randomization.

Also, at the randomization visit, the importance of reporting signs and symptoms associated with bleeding, DVT and PE will be stressed.

Subjects will be counseled on the signs and symptoms associated with DVT, PE and bleeding.

For bleeding events, subjects and family members as appropriate, will be instructed:

- To seek medical attention if they develop bleeding
- To contact the investigative site staff or study investigator before the next dose of study medication is due

Subjects and family members, as appropriate will also be instructed:

- About the subject's risk of DVT and PE
- About the signs and symptoms of DVT and PE
- About the signs and symptoms of arterial thromboembolism

- About the signs and symptoms of myocardial infarction
- About the signs and symptoms of stroke
- To contact the investigative site staff or study investigator as soon as symptoms develop and before the next dose of study medication is due

During the randomization visit, if patient signed the ICF for bio-banking serum, heparin, citrate and EDTA blood will be drawn and sent to the Charité, Campus Benjamin Franklin, Berlin, for coagulation analysis and bio-banking.

If the patient is randomized to the rivaroxaban arm study drug will be dispensed to the patient or treating physician.

Treatment phase

During the therapeutic anticoagulatory treatment phase, visits will be conducted bedside by the site qualified personnel (training and education) as delegated by the principal investigator and as documented in the delegation log.

<u>Day 7 post randomization</u>

A visit will be conducted in all patients on the day 7 post randomization. This visit can be done out of hospital or at the clinic.

Physical exam and clinical status review for suspected outcome events will be completed. Adverse events will be collected as specified in Section 12, an outcome assessment will also be performed, and concomitant medication will be collected as specified in Section 8. Subject counseling provided at the randomization visit will be repeated in detail.

A CTPA is highly recommended if the patient has severe symptom or deteriorates.

EDTA and heparin blood will be drawn to determine hemoglobin/platelets counts, D-dimer, creatinine, AST, ALT, INR, aPTT, hs-CRP, NT-pro-BNP and will be sent to local laboratory if not already taken according to the routine medical care. An ECG can optionally be performed. The drug accountability should also be done during this visit.

New study drug will be dispensed to the patient, if applicable. The clinical condition of the patient will be documented using the seven-category ordinal scale recommended by the WHO for patients with respiratory infections (23-25).

<u>Facultative discharge visit</u>

The facultative discharge visit will only be conducted if the hospital stay is longer than 10 days post randomization.

Physical exam and clinical status review for suspected outcome events will be completed. Adverse events will be collected as specified in Section 12, outcome assessment will also be performed, and concomitant medication will be collected as specified in Section 8. Subject counseling provided at the randomization visit will be repeated in detail.

Heparin and EDTA blood will be drawn to determine serum creatinine, hemoglobin and platelets will be sent to local laboratory, if not already taken according to the routine medical care. An ECG will be performed.

A CTPA is highly recommended if the patient has severe symptom or deteriorates.

New study drug will be dispensed to the patient or medical personnel in charge of the regular medication administration. Study personnel will confirm enough drug supply until discharge. The drug accountability should also be done during these visits.

The clinical condition of the patient will be documented using the seven-category ordinal scale recommended by the WHO for patients with respiratory infections (23-25).

If patient signed the ICF for optional bio-banking, serum, heparin, citrate and EDTA blood will be drawn and sent to the Charité, Campus Benjamin Franklin, Berlin, for coagulation analysis and bio-banking.

35 days post randomization visit

A visit will be conducted in all patients at day 35 (-0 / +5 days) post randomization.

Physical exam and clinical status review for suspected outcome events will be completed. Adverse events will be collected as specified in Section 12, outcome assessment will also be performed, and concomitant medication will be collected as specified in Section 8. Subject counseling provided at the randomization visit will be repeated in detail including also:

- The need to seek for medical attention if any of the symptoms of above-mentioned diseases are developed
- The need to inform the treating health care providers about study participation
- The need to inform the investigator as soon as symptoms develop

EDTA and heparin blood will be drawn to determine hemoglobin/platelets counts, D-dimer, creatinine, AST, ALT, INR, aPTT, hs-CRP, NT-pro-BNP and will be sent to local laboratory, if not already taken according to the routine medical care.

If lung imaging is part of the local routine, it is encouraged to be done as a CTPA to assess the presence of PE. An ECG will optionally be performed.

The clinical condition of the patient will be documented using the seven-category ordinal scale recommended by the WHO for patients with respiratory infections (23-25).

The drug accountability should also be done during this visit.

If patient signed the ICF for optional bio-banking serum, heparin, citrate and EDTA blood will be drawn and sent to the Charité, Campus Benjamin Franklin, Berlin, for coagulation analysis and bio-banking.

Follow-up

EOS Visit (60 days post randomization)

EOS visit will be conducted as a telephone visit, but – if the patient is still hospitalized - can also be done at the clinic. Adverse events will be collected as specified in Section 12, outcome assessment will also be performed, and information about concomitant medication will be collected as specified in Section 8. Subject counseling provided at the randomization, and in hospital visits will be again repeated in detail:

- The need to seek for medical attention if any of the symptoms of above-mentioned diseases are developed
- The need to inform the treating health care providers about study participation
- The need to inform the investigator as soon as symptoms develop

Unscheduled Visit

Subjects may be seen by the investigator between scheduled visits for any reason such as:

- Suspected efficacy or bleeding outcome event
- Early permanent study drug discontinuation
- Adverse event based upon the severity and clinical judgment of the investigator
- Prolonged hospitalization
- Lost medication requiring replacement

Early Permanent Study Drug Discontinuation/Early Withdrawal from Study

If the subject permanently discontinues study drug before day 35 post randomization, he/she should be instructed to complete the scheduled visits.

Because the primary efficacy analysis of the study is based upon the ITT principle, the investigator should inform the subject of the importance of returning for all study visits. It is imperative for the integrity of the trial and results to have vital status and outcomes ascertainment. If the subject is unwilling or unable to return for any visits, the site should collect as much follow-up information as possible, including contacting the subject or legally acceptable representative by telephone or by mail to determine vital status and if an outcome has occurred, as agreed to by the subject during the initial informed consent process. If applicable, vital status and other outcomes should be obtained by reviewing the subject's

medical or public records unless this contact is not allowed by local regulations. If the subject withdraws consent from the study, this must be documented in the source document and the subject will be asked to supplement the withdrawal of consent with a signed written statement documenting refusal for all subsequent contact.

9.2. Efficacy Evaluations and Outcomes

9.2.1. Efficacy Evaluations, Outcomes and Adjudication

The primary endpoint is the following:

- D-dimer at day 7 post randomization

The co-primary endpoint is the following:

- Seven-category ordinal scale recommended by the WHO at day 7 post randomization (Attachment 2)

The secondary combined efficacy endpoint is the following:

- Venous thromboembolism (VTE) (deep venous thrombosis (DVT) and/or fatal or non-fatal pulmonary embolism (PE))
- Arterial thromboembolism
- New myocardial infarction (MI)
- Non-hemorrhagic stroke
- All-cause death
- Progression to intubation and invasive ventilation

Other endpoints of interest:

- Length of hospital stay
- Time to intubation
- Re-hospitalization for heart failure
- Re-hospitalization due to any other reason
- Effects on coagulation parameters for thrombosis (TAT, PAI-1, PF-4, TF, TF-activity)
- Effects on inflammatory and fibrotic parameters (interleukines, interferons, growth factors)
- Change of N-terminal prohormone brain natriuretic peptide (NT-pro-BNP)
- Unscheduled out-patient visits

Any clinical event that suggests the possibility that a secondary efficacy outcome event has occurred should be indicated on the Clinical Status page of the eCRF and will be sent for adjudication. All clinical data must be sent to CEC for adjudication. The CEC may request additional information to ensure appropriate adjudication. The CEC will apply the definitions (see Attachment 4) contained in the CEC charter to adjudicate and classify the events while blinded to treatment assignment. Adjudicated results will be used for final analyses.

9.2.2. Approach to the Subject with a secondary Efficacy Endpoint Event

If a subject has a suspected efficacy endpoint event during the study, the treating physician should exercise clinical judgment and follow established guidelines. At the treating physician's discretion, the routine measures described below may be considered.

- Temporarily interrupt or permanently discontinue study drug treatment as clinically indicated.
- Perform necessary diagnostic procedures and consider the usual treatment measures for VTE and/or cardiovascular (including stroke) events. If physical examination and diagnostic testing suggest benefit these could be obtained.

After clinical evaluation of the suspected secondary efficacy endpoint event is completed restarting study drug may be considered if none of the conditions requiring permanent discontinuation are present (Section 10.2.3, Permanent Discontinuation of Study Treatment), and after consultation with the medical monitor. In the event that a decision is made to restart study drug (Section 10.2.1, Temporary Interruption of Study Treatment), guidelines for restarting study drug (Section 10.2.2, Approach to Subjects with Temporary Interruption of Study Treatment) may be followed if applicable based on the clinical judgment of the investigator.

9.3. Safety Evaluations and Endpoints

9.3.1. Bleeding Events

The study will include the following evaluations of safety and tolerability according to the time points provided in the TIME AND EVENTS SCHEDULE: ISTH fatal or non-fatal major bleeding, non-major clinically relevant bleeding, other non-major bleeding that lead to study drug interruption for more than 7 days, adverse events, and clinical laboratory tests.

The study will use the ISTH Bleeding Event Classification Scale to assess bleeding events as major, non-major clinically relevant bleeding, or other bleeding. Similar to efficacy endpoint, the same independent CEC will adjudicate and classify bleeding events according to definitions in the CEC charter.

The principal safety endpoint for this study is fatal of non-fatal major bleeding using validated ISTH bleeding criteria (27). Other safety endpoints are non-major clinically relevant bleeding and non-major bleeding that lead to study drug interruption for more than 7 days.

An ISTH major bleeding event is defined as overt bleeding that is associated with:

- A fall in hemoglobin of 2 g/dL or more, or
- A transfusion of 2 or more units of packed red blood cells or whole blood, or
- A critical site: intracranial, intraspinal, intraocular, pericardial, intra-articular, intramuscular with compartment syndrome, retroperitoneal, or
- A fatal outcome

Non-major clinically relevant bleeding is defined as overt bleeding not meeting the criteria for major bleeding but associated with medical intervention, unscheduled contact (visit or telephone call) with a physician, (temporary) cessation of study treatment, or associated with discomfort for the subject such as pain or impairment of activities of daily life. Examples of non-major clinically relevant bleeding are:

- Epistaxis if it lasts for more than 5 minutes, if it is repetitive (i.e. 2 or more episodes of true bleeding, i.e. not spots on a handkerchief, within 24 hours), or leads to an intervention (packing, electrocautery, etc.)
- Gingival bleeding if it occurs spontaneously (i.e. unrelated to tooth brushing or eating), or if it lasts for more than 5 minutes
- Hematuria if it is macroscopic, and either spontaneous or lasts for more than 24 hours after instrumentation (e.g. catheter placement or surgery) of the urogenital tract
- Macroscopic gastrointestinal hemorrhage: at least 1 episode of melena or hematemesis, if clinically apparent
- Rectal blood loss, if more than a few spots
- Hemoptysis, if more than a few speckles in the sputum, or
- Intramuscular hematoma
- Subcutaneous hematoma if the size is larger than 25 cm^2 or larger than 100 cm^2 if provoked
- Multiple source bleeding

Other bleeding is defined as any other overt bleeding that does not meet the ISTH criteria for major or non-major clinically relevant bleeding.

9.3.2. Approach to the Subject with a Bleeding Event

If a subject has a serious bleeding event during study drug treatment, the following routine measures should be considered:

- Delay the next study drug administration or discontinue treatment if indicated. Rivaroxaban has a plasma half-life of approximately 5 to 9 hours, and in some subjects up to 13 hours. Therefore, temporary cessation of study drug may allow control of bleeding.
- Consider the usual treatment measures for bleeding events, including fluid replacement and hemodynamic support, blood transfusion, and fresh frozen plasma, if physical examination and laboratory testing suggest benefit could be obtained.
- Consider that other causes besides antithrombotic medication can be contributory to the seriousness of the bleeding event (i.e., rule out disseminated intravascular coagulation, thrombocytopenia, and other coagulopathies; kidney and liver dysfunction; concomitant medications, etc.), and treat accordingly.

If bleeding cannot be controlled by the above measures, please refer to summary of product characteristics of the respective study drug.

Any products administered to control bleeding should be entered in the eCRF.

After resolution of the bleeding event, restarting of study drug may be considered based on the clinical judgment of the investigator.

9.3.3. Other Safety Assessments

Adverse Events (AE)

AEs will be reported by the subject (or, when appropriate, by a caregiver, surrogate, or the subject's legally acceptable representative or the attending physician) for the duration of the study. AEs will be followed by the investigator as specified in Section 12, Adverse Event Reporting.

Clinical Laboratory Tests

Separate laboratory screening tests are not expected to be performed by the investigator as these will likely be part of the subjects' hospital evaluation. No pre-specified laboratory tests will be performed for the duration of the study. However, these subjects are likely to have local laboratory tests performed during their index hospitalization. Any laboratory test along with reference ranges relevant to a serious adverse event or an outcome event should be recorded on the appropriate eCRF page.

The following tests results with reference ranges will be obtained from the hospital laboratory/local laboratory at the time of the index hospitalization:

- Hematology
    - hemoglobin
    - platelet count
- Serum creatinine
- D-dimer
- Hs-cTnT
- Serum or urine pregnancy testing for women of childbearing potential only

9.4. Benefit-Risk Assessment

Benefit-risk of rivaroxaban in the setting of COVID-19 will be explored.

10. SUBJECT COMPLETION / WITHDRAWAL

10.1. Completion

The total duration of the study for a subject who completes the study after randomization is 60 days. All feasible efforts and measures will be made to collect complete vital status and other outcome data from randomization to the EOT/EOS visit for each subject randomized in this study regardless of compliance with study drug or visits. For subjects who are lost to follow-up or withdraw consent from the study, efforts will be made to obtain their vital status and other outcomes from permitted sources.

It is important to note that withdrawal of consent does not withdraw permission to collect vital status and other outcomes. Withdrawal of permission to collect vital status and other outcomes must be made separately. In cases where subjects indicate they do not want to "continue", investigators must

determine whether this refers to discontinuation of study treatment (the most common expected scenario), unwillingness to attend follow-up visits, unwillingness to have telephone contact, unwillingness to have any contact with study personnel, or unwillingness to allow contact with a third party (e.g., family member, doctor). In all cases, every effort must be made to continue to follow the subject, and vital status and other outcomes must be determined for all randomized subjects.

10.2. Discontinuation of Study Treatment

If a subject's study treatment must be discontinued before the end of the treatment or thromboprophylaxis phase, the subject must continue to be followed for efficacy and safety endpoint. During the study, should the subject develop any condition, which in the investigator's judgment requires long-term therapeutic anticoagulation, or fibrinolysis, the subject will have the study drug either temporarily interrupted or permanently discontinued and will be managed as deemed appropriate by the treating physician. The subject will be asked to continue in the study to be followed for efficacy as well as safety endpoint.

10.2.1. Temporary Interruption of Study Treatment

If the subject requires (re)-hospitalization (hospitalization is defined as a combined total of inpatient and/or emergency room stay ≥24 hours) during the study drug treatment for reasons other than the primary efficacy endpoints or bleeding, the subject should be maintained on the study drug or SOC treatment at the discretion of the investigator. If the treating physician feels that the subject requires any anticoagulation or another anticoagulation during the re-hospitalization, study drug needs to be temporarily interrupted, but can be resumed upon hospital discharge and stop of any prohibited medication.

In addition, the study drug should be temporarily interrupted if the subject:

- Develops any medical condition that may require use of anticoagulant therapy, thromboprophylaxis, fibrinolysis or poses an increased bleeding risk.
- Undergoes percutaneous coronary intervention, coronary artery bypass graft, any other interventional procedure, that may require use of anticoagulant therapy, thromboprophylaxis, or poses an increased bleeding risk.
- For temporary interruption of study drug for an elective procedure or surgery, it is suggested that investigators follow measures in Table 1 and Table 2 (Section 10.2.2 Approach to Subjects with Temporary Interruption of Study Treatment).
- Experiences a major bleeding event other than intracranial bleeding. For less severe bleeding events, investigator discretion is allowed. If possible, study drug should be resumed when the bleeding event has resolved, and the cause has been identified and corrected.
- If a subject develops a new neurologic deficit or significant alteration in mental status suggesting a cerebral vascular accident. Once a diagnosis is definitively made and appropriate treatment is provided, the study drug may be restarted at the discretion of the investigator.
- Develops a platelet count less than 50,000/µl. If a repeat platelet count is obtained, and the result indicates that the abnormal platelet count was spurious, study drug may be restarted. If the finding was not spurious, study drug may be restarted after 2 consecutive values greater than 75,000/µl apart have been obtained.
- Has a serious adverse event that is considered by the investigator to be possibly related to or exacerbated by study drug administration.
- Requires a prohibited therapy on a temporary basis (see Section 8, Prestudy and Concomitant Therapy).

10.2.2. Approach to Subjects with Temporary Interruption of Study Treatment

The clinical condition of patients with moderate to severe COVID-19 may deteriorate during the hospital stay and the subject may need supportive care on an ICU. In critical cases in need of mechanical ventilation, shock or organ failure requiring ICU care, rivaroxaban treatment will be switched to full dose anticoagulation with LMWH or UFH if treating physicians consider the bleeding risk not as very high. In case the bleeding risk is considered higher the patients would be switched to prophylactic anticoagulation.

If the patient's condition improves during the course of the disease, the treating physician should restart rivaroxaban at the earliest possible time point judged as safe. However, it is also possible to discontinue and stop the treatment with rivaroxaban, if there are safety concerns at the discretion of the treating physician. If the study drug is temporarily interrupted to allow a procedure to be performed, the routine measures described in Table 1 and Table 2 should be considered:

Table 1: Preoperative Interruption of Rivaroxaban: A Suggested Management Approach (adapted from Spyropoulos and Douketis 2012)

Patient Renal Function	Low Bleeding Risk Surgery (2-3 drug half-lives between last dose and surgery) [a]	High Bleedíng Risk Surgery (4-5 drug half-lives between last dose and surgery) [b]
Normal or mild impairment (CrCl ≥50 mL/min), or moderate (CrCl ≥30 and <50 mL/min) impairment	Last dose: 2 days before surgery (skip 1 dose)	Last dose: 3 days before surgery (skip 2 doses)

CrCl, creatinine clearance.

[a] aiming for mild-to-moderate residual anticoagulant effect at surgery (<12-25%).

[b] aiming for no or minimal residual anticoagulant effect (<3-6%) at surgery.

Table 2: Postoperative Resumption of Rivaroxaban: A Suggested Management Approach

Low Bleeding Risk Surgery	High Bleeding Risk Surgery
resume on evening of or day after surgery (within 24 hours postoperative)	resume 2 to 3 days after surgery (within 48 to 72 hours postoperative)

10.2.3. Permanent Discontinuation of Study Treatment

If a subject must be permanently discontinued from study drug before the end of the thromboprophylaxis phase, this will not result in automatic withdrawal of the subject from the study, and the subject should continue to be followed for efficacy and safety outcomes (for details see Section 3.1).

A subject should be permanently discontinued from study drug if:

- The investigator believes that for safety reasons (i.e., adverse event) it is in the best interest of the subject to stop study drug.
- The subject develops any condition which requires anticoagulation or thromboprophylaxis extending beyond the treatment phase of the study (e.g., atrial fibrillation, VTE).
- The subject becomes pregnant.
- The subject has a fall in eGFR to below 20 mL/min/1.73m^2 or 2 consecutive measurements less than 30 mL/min/1.73m^2 at least 1 week apart (as calculated by the CKD-EPI formula) during the study.
- The subject requires hemofiltration or dialysis on a permanent basis
- The subject requests to discontinue study drug permanently
- The subject has a hemorrhagic stroke, or intracranial bleeding

If the subject permanently discontinues study drug before Day 35 post randomization, he/she should be instructed to complete an unscheduled visit and the remaining scheduled visits (if not yet completed).

The eCRF is to be completed to identify the reason for permanent discontinuation of study drug. The investigator will provide a narrative to describe any adverse event that occur up to the 35-day post randomization visit. The appropriate adverse event or serious adverse event sections of the eCRF are to be completed. If study drug is terminated for a serious adverse event, expedited reporting (within 24 hours) is also required as outlined in Section 12.3.2, Serious Adverse Events.

10.3. Withdrawal from the Study

A subject will be withdrawn from the study for any of the following reasons:

- Lost to follow-up (only after all means of all subsequent contact, including locator services where permitted by law, up until the day 60 post randomization, have been exhausted, lost to follow-up will be declared).
- Withdrawal of consent (unless specifically refused by the subject, subject contact will be made to obtain vital status and other outcomes until the day 60 post randomization visit).
- If during the study the subject loses his ability to consent (eg intubation). In this case, after the subject recovers his/her ability to consent, he/she will be asked again to consent and continue participating actively in the trial.

If a subject is lost to follow-up, every reasonable effort must be made by the study site personnel to contact the subject and determine the reason for discontinuation/withdrawal. The measures taken to follow up must be documented.

Study drug assigned to the withdrawn subject may not be assigned to another subject. Subjects who withdraw will not be replaced.

If a subject withdraws consent from study or is lost to follow-up, his or her vital status and other outcomes will be collected at day 35 post randomization either by telephone or in person, or if applicable, by a review of the subject's medical or public records unless this contact is not allowed by local regulations.

11. STATISTICAL METHODS

This is an explorative study and hence analyses have an explorative nature. In the following we describe the principles for the planned analyses. Further details will be documented in a statistical analysis plan (SAP).

11.1 Definition of populations included in the analyses

The primary efficacy analysis as well as all secondary efficacy analyses will be based on the full analysis set (FAS) following the intention-to-treat principle and will include all randomized patients. Safety analyses will be based on the safety set (SAF) including all randomized patients with at least one dose of study medication or at least one dose of SOC (UFH or LMWH).

For time-to-event endpoints, study discontinuations will be dealt with as independent right censoring. Patients discontinuing study drug will be follow up for endpoints. Otherwise missing values will be dealt with using multiple imputation methods. If, against expectations, the proportion of patients with premature study discontinuations is substantial, shared random effects models simultaneously modelling time to primary outcome and time to study discontinuation will be used to explore the robustness of the treatment effect.

Sensitivity analyses will be performed in all patients (a) completing at least 7 days of treatment and (b) completing the study adherent to the study protocol. These will be referred to as the (a) while on treatment population and (b) per-protocol (PP) population.

11.2 Primary and Co-primary Efficacy Endpoints

The primary endpoint time is

- D-dimer at day 7 post randomization

The co-primary endpoint is the following:

- Seven-category ordinal scale recommended by the WHO at day 7 post randomization (Attachment 2)

The primary efficacy endpoint is D-dimer at day 7 post randomization will adjusted for baseline measurement. The logarithmic D-dimer measurements (using the natural logarithm) will be analyzed by an analysis of covariance (ANCOVA). The model will include treatment group and stratification variables of the randomization as factors and the logarithmic baseline D-dimer measurement as covariate. Least squares means for D-dimer at day 7 in the two treatment groups will be presented with 95% confidence intervals as well as the difference between the treatment groups at day 7 with 95% confidence interval and p-value testing the null hypothesis of no treatment difference, i.e. a mean difference (on the

logarithmic scale) of 0. For ease of interpretation the results will also be converted to the original measurement scale.

The co-primary endpoint is the seven-category ordinal scale recommended by the WHO (Attachment 2) at day 7 post randomization adjusted for baseline score. Frequencies and percentages of the scores at day 7 will be provided stratified by treatment group. The distributions of the scores will be compared between the two treatment groups using a nonparametric Wilcoxon (Mann-Whitney) rank-sum test stratified by baseline score dichotomized as smaller or equal 3 vs. larger 3. The treatment effect will be reported as probabilistic index or relative effect, i.e. the probability that an observation in the treatment group will be smaller (more favourable) than an observation in the control group, with 95% confidence interval.

11.3 Secondary Efficacy Endpoint

The secondary combined endpoint includes

Time to first event of either

- Venous thromboembolism (VTE) (deep venous thrombosis (DVT) and/or fatal or non-fatal pulmonary embolism (PE))
- Arterial thromboembolism
- New myocardial infarction (MI)
- Non-hemorrhagic stroke
- All-cause death
- Progression to intubation and invasive ventilation

Time to first event of either of the components of the secondary composite endpoint will be analyzed using a Cox proportional hazards regression model with treatment and the stratification variables of the randomization (site, gender (male, female, diverse), age (< 65 versus ≥ 65 years), kidney function (eGFR ≥ 30 mL/min/1.73m^2 and < 50 mL/min/1.73m^2 versus eGFR ≥ 50 mL/min/1.73m^2), history of CAD or heart failure (yes/no), oxygen demand on admission to the hospital (supplementary oxygen required versus not required), setting (outpatients versus hospitalized patients)) as factors. The treatment effect will be reported as hazard ratio (HR) with 95% confidence interval and p-value for the null hypothesis H0: HR=1. The primary endpoint will be visualized as Kaplan-Meier curves. Sensitivity analyses will explore the robustness of these analyses.

The analyses of the time-to-event outcomes among the secondary endpoints (such as time to VTE, time to arterial thromboembolism, time to new MI, time to non-hemorrhagic stroke, time to all-cause death, time to progression to intubation and invasive ventilation) will follow the same lines as the analysis of the composite outcome, i.e. survival analyses comprising Cox proportional hazard regression models and Kaplan-Meier curves. Death will be treated as a competing event except when analyzing all-cause death, naturally.

Longitudinal D-dimer measurements assessed at day 7, 35 and 60 will be analyzed on the logarithmic scale using a mixed model for repeated measures (MMRM) approach. The model will include treatment, visit, treatment-by-visit interaction and stratification variables of the randomization as factors as well as logarithmic D-dimer baseline value as covariate. The residuals will be assumed to follow a multivariate normal distribution with unstructured covariance matrix. LS means of the group means and their differences will computed for the various timepoints and reported with 95% confidence intervals.

Longitudinal ordinal data (a seven-category ordinal scale) assessed at day 7, 35 and 60 will be analyzed using a proportional odds model with treatment, visit, treatment-by-visit interaction, baseline score and stratification variables of the randomization as factors. The correlation in longitudinal assessments within the same patient will be accounted for by random patient effects. Binary data (e.g. development of DIC) will use similar generalized linear mixed effect models but with logit link function.

All stratification variables of the randomization (site, gender (male, female, diverse), age (< 65 versus ≥ 65 years), kidney function (eGFR ≥ 30 mL/min/1.73m^2 and < 50 mL/min/1.73m^2 versus eGFR ≥ 50 mL/min/1.73m^2), history of CAD or heart failure (yes/no), oxygen demand on admission to the hospital (supplementary oxygen required versus not required), setting (outpatients versus hospitalized patients)) will be included as factors in the analyses of the secondary endpoints.

11.4 Subgroup analyses

Subgroup analyses are planned to explore a possible heterogeneity of the treatment effect concerning the following baseline characteristics: setting (outpatients versus hospitalized patients), gender, age (< 65 versus ≥ 65 years), kidney function (eGFR ≥ 30 mL/min/1.73m^2 and < 50 mL/min/1.73m^2 versus eGFR ≥ 50 mL/min/1.73m^2), smoking status, diabetes, presence of coronary heart disease or heart failure (yes/no), need for oxygen treatment at baseline (supplementary oxygen required versus not required), inclusion based on D-dimer, hs-cTnT or both. For subgroup analyses the respective baseline characteristic and its interaction with treatment are included in the regression models described above for the primary and secondary endpoints. Treatment effect heterogeneity across subgroups will also be visually inspected in forest plots.

11.5 Safety Endpoints

Safety analyses will follow standard procedures for the reporting of adverse events; these will be reported as frequencies (percentages) by treatment group. All safety analyses will be based on the safety set. One safety endpoint of particular interest are bleeding events (see Section 9.3.1). The time to bleeding events will be analyzed in a Cox proportional hazards model, if a sufficient number of events will be observed. Treatment group differences will be reported as hazard ratios with 95% confidence intervals and p-values testing the null hypothesis of no group difference (i.e. HR=1). The estimation of the probability for bleeding events will account for competing events such as death and premature study discontinuation using the Aalen-Johansen estimator, the so-called cumulative incidence function.

Differences between treatment groups in continuous laboratory parameters and vital signs over follow-up time will be explored in longitudinal box plots.

11.6 Additional Endpoints

Other endpoints of interest include

- Length of hospital stay
- Time to intubation
- Re-hospitalization for heart failure
- Re-hospitalization due to any other reason
- Effects on coagulation parameters for thrombosis (TAT, PAI-1, PF-4, TF, TF-activity)
- Effects on inflammatory and fibrotic parameters (interleukines, interferons, growth factors)
- Change of N-terminal prohormone brain natriuretic peptide (NT-pro-BNP)
- Unscheduled out-patient visits

Details on the analysis of those endpoints will be described in the statistical analysis plan (SAP).

11.7 Sample size determination

Sample size calculation is based on the primary endpoint D-dimer at day 7 corrected for baseline values. Since the distribution of D-dimer concentrations is skewed, calculations are done on the log scale. Using an analysis of covariance (ANCOVA) (significance level alpha=0.05 two-sided) with baseline values as covariate a total sample size of 80, i.e. 40 per group, gives a power of 80% to detect a mean difference of 0.44 (on the log scale) between the treatment group and the control group at day 7. Based on a blinded review, a common standard deviation of SD=0.8 and a correlation to baseline of r=0.5 (both on the natural log scale) were assumed. The effect assumed translates to a reduction of 36% in D-Dimer values on the original scale. The co-primary endpoint is a seven-category ordinal scale previously recommended by the WHO (Attachment 2) as clinical improvement scale for patients with respiratory infections. Here 40 patients per group will give a power of 80% to detect a relative treatment effect p1=0.68 between treatment and control group at day 7 using a Wilcoxon (Mann-Whitney) rank-sum test (two sided, alpha=0.05). The probabilistic index or relative effect p1 denotes the probability that an observation in the treatment group will be less than an observation in the control group. Targeting a total of at least 80 evaluable patients, we aim to recruit about 100 patients.

12. ADVERSE EVENT REPORTING

Timely, accurate, and complete reporting and analysis of safety information from clinical studies are crucial for the protection of subjects, investigators, and the sponsor, and are mandated by regulatory agencies worldwide. The sponsor has established Standard Operating Procedures in conformity with regulatory requirements in Germany to ensure appropriate reporting of safety information.

Rivaroxaban has been extensively studied in Phase 1 through Phase 4 clinical studies involving more than 100.000 patients and its overall adverse event profile has been well described. The comparator treatment with UFH/ LMWH as standard of care has a well described safety profile. Appropriate information concerning adverse events were systematically collected and submitted to regulatory authorities. For the purposes of this study (and after discussion with appropriate regulatory agencies) a value-driven approach to safety data collection will be utilized.

12.1 Adverse Events (AEs)

12.1.1. Definitions

Adverse Event (AE)

An AE is any untoward medical occurrence in a clinical study subject who is receiving a medicinal (investigational or non-investigational) product. An AE does not necessarily have a causal relationship with the treatment. An AE can therefore be any unfavorable and unintended sign (including abnormal laboratory finding), symptom, or disease temporally associated with the use of a medicinal (investigational or non-investigational) product, whether or not related to that medicinal (investigational or non-investigational) product (Definition per International Conference of Harmonization (ICH)).

This includes any occurrence that is new in onset or aggravated in severity or frequency from the baseline condition, or abnormal results of diagnostic procedures, including laboratory test abnormalities.

Note: The sponsor collects adverse events starting with the signing of the ICF (refer to Section 12.3.1, All Adverse Events, for time of last adverse event recording).

Unlisted (Unexpected) Adverse Reactions/ Reference Safety Information

Adverse reactions are all untoward unintended responses to an IMP related to any dose administered. An AE is considered associated with the use of a drug if the attribution is possible, probable, very likely or not assessable by the definitions listed in Section 12.1.2.3.

An adverse reaction is considered unlisted if the nature or severity is not consistent with the applicable product reference safety information. For Rivaroxaban, the expectedness of an AE will be determined by whether it is listed in the Investigator's Brochure. For UFH/ LMWH the expectedness of an AE will be determined by whether it is listed in the corresponding SmPC (section 4.8).

Serious Adverse Event (SAE)

A SAE based on ICH and European Union (EU) Guidelines on Pharmacovigilance for Medicinal Products for Human Use is any untoward medical occurrence that at any dose:

- Results in death
- Is life-threatening (The subject was at risk of death at the time of the event. It does not refer to an event that hypothetically might have caused death if it were more severe)
- Requires in-patient hospitalization or prolongation of existing hospitalization
- Results in persistent or significant disability / incapacity (Disability means a substantial disruption of a person’s ability to conduct normal life’s functions.)
- Is a congenital anomaly / birth defect
- Is another serious or important medical event as judged by the investigator

*Medical and scientific judgment should be exercised in deciding whether expedited reporting is also appropriate in other situations, such as important medical events that may not be immediately life threatening or result in death or hospitalization but may jeopardize the subject or may require intervention to prevent one of the other outcomes listed in the definition above. These should usually be considered serious.

If an AE is classified as serious, this must be documented on a separate SAE form in addition to the standard AE documentation. The sponsor must be notified of SAEs according to Section 12.3.2.

Suspected Unexpected Serious Adverse Reactions (SUSAR)

A SUSAR is any suspected adverse reaction related to the study treatment that is both serious and unexpected. “Unexpected” means that the nature and severity of the adverse reaction are not consistent with the information about the study medication in question set out in the reference safety information.

12.1.2 Classification for Adverse Events (AE) assessment

All (Serious) AEs will be assessed and documented by the investigator according to the categories detailed below.

12.1.2.1. Seriousness

Seriousness shall be determined according to the SAE criteria given above.

12.1.2.2. Intensity

The intensity of an AE is classified according to the following categories:

- Mild: Awareness of symptoms that are easily tolerated, causing minimal discomfort and not interfering with everyday activities.
- Moderate: Sufficient discomfort is present to cause interference with normal activity.
- Severe: Extreme distress, causing significant impairment of functioning or incapacitation. Prevents normal everyday activities.

The investigator should use clinical judgment in assessing the severity of events not directly experienced by the subject (e.g. laboratory abnormalities).

12.1.2.3 Causal Relationship

The assessment of the causal relationship between an AE and the administration of treatment is a decision to be made by the investigator, based on all information available.

To assess causality between administration of the investigational product and the AE following definitions apply:

- Not Related: An adverse event that is not related to the use of the drug.
- Doubtful: An adverse event for which an alternative explanation is more likely, e.g. concomitant drug(s), concomitant disease(s), or the relationship in time suggests that a causal relationship is unlikely.
- Possible: An adverse event that might be due to the use of the drug. An alternative explanation, e.g., concomitant drug(s), concomitant disease(s), is inconclusive. The relationship in time is reasonable; therefore, the causal relationship cannot be excluded.
- Probable: An adverse event that might be due to the use of the drug. The relationship in time is suggestive (e.g., confirmed by dechallenge). An alternative explanation is less likely, e.g. concomitant drug(s), concomitant disease(s).
- Very Likely: an adverse event that is listed as a possible adverse reaction and cannot be reasonably explained by an alternative explanation, e.g. concomitant drug(s), concomitant disease(s). The relationship in time is very suggestive (e.g. it is confirmed by dechallenge and rechallenge).
- Not assessable: Cannot be judged because information is insufficient or contradictory.

12.1.2.4 Action taken with study treatment

Any action on study treatment to resolve the AE is to be documented using the categories listed below.

The study treatment action should be recorded separately for each study treatment.

- Drug withdrawn
- Drug interrupted

- Dose reduced
- Dose not changed
- Dose increased
- Not applicable
- Unknown

12.1.2.5. Other specific treatment(s) of AE

- None
- Remedial drug therapy
- Other

12.1.2.6. Outcome

The outcome of the AE is to be documented as follows:

- Recovered/resolved
- Recovering/resolving
- Recovered/resolved with sequelae
- Not recovered/not resolved
- Fatal
- Unknown

12.1.3. Assessments and documentation of AEs

The investigator has to record AEs as described under Section 12.1.1 occurring in the period between the signing of the ICF and the end of the follow-up phase. The type of information that should be assessed and recorded by the investigator for each AE is listed in Section 12.1.2.

For all SAEs the sponsor has to carry out a separate assessment for expectedness, seriousness, and causal relationship to study drug Rivaroxaban or UFH/ LMWH.

12.2. Reporting of SAEs/AEs

The following rules apply to AE and SAE handling:

- All AEs including secondary efficacy endpoints (including complications of efficacy or safety endpoints events) that fulfill the seriousness criteria have to be recorded on the AE page of the eCRF and reported within 24 hours of the investigator's awareness.
- All primary and secondary safety endpoints (i.e. bleedings) have to be recorded in the eCRF. All bleedings that fulfill the seriousness criteria, e.g. fatal, non-fatal major bleedings as well as clinically relevant bleedings which are defined as AEs of Special Interest have to be reported within 24 hours of the investigator's awareness.
- All pregnancies in female study subjects or in female partner of a male study subject as well as endpoints of the pregnancies have to be reported within 24 hours of the investigator's awareness.
- Of the AEs reported, reportable events (i.e. SUSARs) have to be reported to the competent authorities and to the Independent IECs/IRBs according to legal requirements.

The investigator must report immediately (within 24 hours of the investigator's awareness) all AEs/SAEs described above as requiring expedited reporting. The report recipients are detailed in Section 12.4. However, non-serious AEs that the investigator considers of particular concern may also be reported to bring them to the attention of the sponsor.

The investigator is responsible for continuous monitoring of all SAE reports (whether or not related to study drug) until resolution or until the event is considered chronic and/or stable by the investigator and/or other physician who has the responsibility for the subject's medical care. Follow-up SAE reports

will be reported according to the same timelines as initial reports, as soon as new significant information becomes available.

Safety events of interest on a study drug that may require expedited reporting and/or safety evaluation include, but are not limited to:

- Overdose of a study drug
- Suspected abuse/misuse of a study drug
- Inadvertent or accidental exposure to a study drug
- Any failure of expected pharmacologic action (i.e. lack of effect) of a study drug
- Medication error involving a product (with or without subject/patient exposure to the study drug, e.g. name confusion)

These special reporting situations should be recorded in the eCRF. Any special reporting situation that meets the criteria of a SAE should be recorded and reported as SAE (Section 12.3.2).

12.2.1 Adverse Events of Special Interest (AESI)

The following events are considered as AEs of Special Interest:

- Fatal and non-fatal major bleedings as well as clinically relevant bleedings

These AESIs (even if classified as non-serious) have to be recorded and reported as described in Section 12.2.

12.3. Procedures

12.3.1 Reporting Timelines and Processes

All (serious) adverse events ((S)AEs) and special reporting situations, whether serious or non-serious, will be reported from the time a signed and dated ICF is obtained until completion of the subject's last study-related procedure.

All AEs, regardless of seriousness, severity, or presumed relationship to study drug, must be recorded using medical terminology in the source document. Whenever possible, diagnoses should be given when signs, symptoms, and laboratory changes are due to a common etiology/ specific disease. The documentation needs to include the type of event, the date and time of onset, duration and resolution, interruption or withdrawal of study treatment and other measures taken.

Investigators must record their opinion concerning the relationship of the AE to study therapy. All measures required for AE management must be recorded in the source document and eCRF and reported according to sponsor instructions. All AEs should be treated appropriately. Such treatment may include changes in study drug treatment including possible interruption or discontinuation, starting or stopping concomitant treatments, changes in the frequency or nature of assessments, hospitalization or any other medically required intervention.

All (S)AEs need to be followed until they subside or stabilize and assessment should be made at each visit (or more frequently, if necessary) of any changes in severity, the suspected relationship to the study, the interventions required to treat it and the outcome.

12.3.2. SAEs and AESI reporting

All SAEs (as defined above) as well as all AESIs (as defined above) occurring during the study must be reported to the appropriate sponsor contact person by study site personnel within 24 hours of their knowledge of the event.

Information regarding SAEs will be transmitted to the sponsor using the Serious Adverse Event Form, which must be completed and signed by a physician from the study site and transmitted to the sponsor within 24 hours. If at that time point all required information is not yet available, succeeding records will be sent. In the event of death, a copy of the autopsy record should be added.

The initial and follow-up reports of a SAE should be made by fax or by e-mail (contact details see Section 12.4). Follow-up information is sent using a new SAE Form stating that this is a follow-up to the

previously reported SAE and giving the date of the original report. Each change, complication, or progression of the original event should be reported as a follow-up to that event regardless of when it occurs. The follow-up information should describe whether the event has resolved or continues, if and how it was treated and whether the patient continued or withdrew from trial participation.

The investigator's SAE report will be examined by the sponsor for completeness and formal plausibility. If required, queries are made and followed up.

All cases of suspected SAEs are assessed by the sponsor with regard to seriousness, causality, and expectedness. Possible signals could be detected by carefully reviewing all available information (reported events, clinical database) at regular intervals (e.g. before a DSMB meeting, DSUR) according to internal procedures.
SAE reports of fatal and non-fatal major bleedings as well as clinically relevant bleedings will be forwarded by the sponsor to BAYER AG in a timely manner.

12.3.3. SUSAR reporting

The sponsor assumes responsibility for appropriate reporting of adverse events to the regulatory authorities.

Suspected Unexpected Serious Adverse Reactions (SUSARs) will be recorded and reported to the competent authorities and relevant ethics committees as well as to all investigators involved in the trial in accordance with EU Guidance 2011/C 172/01 or as per national regulatory requirements in participating countries.

12.3.4. Data Safety Monitoring Board (DSMB) / Clinical Events Committee (CEC)

DSMB and CEC (refer to Section 3.3), composed of independent experts without conflict of interests, will be established to monitor continuously the safety of study participants and to ensure scientific and ethical standards. The Committees will meet regularly (according to corresponding charters) to review study data to evaluate the safety, efficacy, study progress, and conduct of the study. The Sponsor will update the Committees on all reported SAEs, outcome events, and safety information on a near real time basis. The Committees will consider all information regarding benefits and risks and will judge the regular information on efficacy and safety data in the context of newly emerging evidence on optimal management of patients with COVID-19.

The Committees will formulate recommendations to the sponsor relating to the rates of accrual, eligibility of clinical trial participants, adherence to protocol and adjudication of trial endpoints and regarding trial continuation, discontinuation or amendments.

12.3.5. Pregnancy

The reporting of pregnancies is described in Section 12.2. All initial reports of pregnancy must be reported to the sponsor by the study-site personnel within 24 hours of their knowledge of the event using the appropriate pregnancy notification form. Abnormal pregnancy outcomes (e.g. spontaneous abortion, stillbirth, and congenital anomaly) are considered SAEs and must be reported using the Serious Adverse Event Form. Any subject who becomes pregnant during the study must discontinue further study treatment.

Because the effect of the study drug on sperm is unknown, pregnancies in partners of male subjects included in the study will be reported by the study-site personnel within 24 hours of their knowledge of the event using the appropriate pregnancy notification form.

Follow-up information regarding the outcome of the pregnancy and any postnatal sequelae in the infant will be required.

12.4. Contacting Sponsor Regarding Safety

The Pharmacovigilance – CTO Charité should be contacted regarding any safety issues:

Pharmacovigilance - Clinical Trial Office (CTO) Charité

Email: pv-cto@charite.de

Fax: +49 30 / 450 7554 484

13. PRODUCT QUALITY COMPLAINT HANDLING

A product quality complaint (PQC) is defined as any suspicion of a product defect related to manufacturing, labeling, or packaging, i.e. any dissatisfaction relative to the identity, quality, durability, or reliability of a product, including its labeling or package integrity. A PQC may have an impact on the safety and efficacy of the product. Timely, accurate, and complete reporting and analysis of PQC information from studies are crucial for the protection of subjects, investigators, and the sponsor, and are mandated by regulatory agencies worldwide. The sponsor has established procedures in conformity with regulatory requirements worldwide to ensure appropriate reporting of PQC information; all studies conducted by the sponsor or its affiliates will be conducted in accordance with those procedures.

13.1. Procedures

All initial PQCs must be reported to the sponsor by the study-site personnel within 24 hours after being made aware of the event. If the defect is combined with a serious adverse event, the study-site personnel must report the PQC to the sponsor according to the serious adverse event reporting timelines (refer to Section 12.3.2, Serious Adverse Events). A sample of the suspected product should be maintained for further investigation if requested by the sponsor.

13.2. Contacting Sponsor Regarding Product Quality

The names (and corresponding telephone numbers) of the individuals who should be contacted regarding product quality issues are listed on the Contact Information page(s), which will be provided as a separate document.

14. STUDY DRUG INFORMATION

14.1. Physical Description of Study Drug(s)

The study design is based on 2 treatment arms: The study drug supplied for one treatment arm is rivaroxaban 20 mg, rivaroxaban 15 mg and rivaroxaban 10 mg (Refer to the SmPC or Investigator's Brochure). The study drug supplied to the other treatment arm is LMWH or UFH. The physicians of the respective trial site decide which heparin, of the heparins approved in Germany, the patients assigned to treatment group "SOC with prophylactic UFH or LMWH" will receive.

14.2. Preparation Handling and Storage

This medicinal product does not require any special storage conditions.

14.3. Drug Accountability

The investigator is responsible for ensuring that all study drug received at the site is inventoried and accounted for throughout the study. The dispensing of study drug to the subject, and the return of study drug from the subject must be documented on the drug accountability form. Subjects must be instructed to return all original containers, whether empty or containing study drug.

Study drug must be handled in strict accordance with the protocol and the container label and must be stored at the study site in a limited-access area or in a locked cabinet. Unused study drug must be available for verification by the sponsor's study site monitor during on-site monitoring visits. The return to the sponsor of unused study drug will be documented on the drug return form. When the study site is an authorized destruction unit and study drug supplies are destroyed on-site, this must also be documented on the drug return form.

Study drug should be dispensed under the supervision of the investigator or a qualified member of the study-site personnel. Study drug will be supplied only to subjects participating in the study. Returned study drug must not be dispensed again, even to the same subject. Study drug may not be relabeled or reassigned for use by other subjects. The investigator agrees neither to dispense the study drug from, nor store it at any site other than the study sites agreed upon with the sponsor.

15. ETHICAL ASPECTS

15.1. Study-Specific Design Considerations

Potential subjects will be fully informed of the risks and requirements of the study and, during the study, subjects will be given any new information that may affect their decision to continue participation. They will be told that their consent to participate in the study is voluntary and may be withdrawn at any time with no reason given and without penalty or loss of benefits to which they would otherwise be entitled. Only subjects who are fully able to understand the risks, benefits, and potential adverse events of the study, and provide their consent voluntarily will be enrolled.

As previously stated, patients infected with SARS-CoV-2 and suffering from a moderate to severe course have an increased risk of thromboembolic events. The bleeding risk in COVID-19 patients is expected to be comparable to the average age-adjusted population. However, pulmonary hemorrhage has been observed in some cases although pro-coagulatory effects seem to be predominating. Exclusion criteria (see Section 4.2.) were designed to protect the most vulnerable population concerning bleeding risk. The procedures for study drug discontinuation are mentioned in section 10.2.

The global COVID-19 pandemic is ongoing and scientific data still rare. Therefore, the investigators will continuously search the literature for new scientific evidence to ensure patient safety and if needed adapt the study protocol.

Should the subject develop any condition which in the investigator’s judgment requires therapeutic anticoagulation or fibrinolysis, the subject will have study treatment discontinued and will be managed as deemed appropriate by the treating physician. The subject will be asked to continue in the study to be followed for efficacy and safety endpoints.

Investigators should inform the subject of the importance to complete all study visits even if their study drug is discontinued prematurely due to an adverse event, or other reasons, to assess the vital status and determine if outcome events may have occurred. If these subjects refuse office visits, the investigator should remind the subject about the importance of allowing regular contact until study end, according to the TIME AND EVENTS SCHEDULE, either with them, or with a legally acceptable representative, a close friend or relative, or their primary care physician to determine vital status and if an efficacy or safety endpoint has occurred. Nevertheless, since participation and withdrawal is voluntary, there should be no pressure on participants to continue participation in the study.

15.2. Regulatory Ethics Compliance

15.2.1. Investigator Responsibilities

The study will be conducted in line with the current ICH guidelines on Good Clinical Practice (GCP), and applicable regulatory and country-specific requirements. Good Clinical Practice is an international ethical and scientific quality standard for designing, conducting, recording, and reporting studies that involve the participation of human subjects. Compliance with this standard provides public assurance that the rights, safety, and well-being of study subjects are protected, consistent with the principles that originated in the Declaration of Helsinki, and that the study data are credible.

15.2.2. Independent Ethics Committee or Institutional Review Board

Before the start of the study, the investigator (or sponsor where required) will provide the IEC/IRB with current and complete copies of the following documents (as required by local regulations):

- Final protocol and, if applicable, amendments
- Sponsor-approved ICF (and any other written materials to be provided to the subjects)
- Investigator's Brochure (or equivalent information) and amendments/addenda
- Sponsor-approved subject recruiting materials
- Information on compensation for study-related injuries or payment to subjects for participation in the study, if applicable
- Investigator's curriculum vitae or equivalent information (unless not required, as documented by the IEC/IRB)

- Information regarding funding, name of the sponsor, institutional affiliations, other potential conflicts of interest, and incentives for subjects
- Any other documents that the IEC/IRB requests to fulfill its obligation

This study will be undertaken only after the IEC/IRB has given full approval of the final protocol, amendments (if any, excluding the ones that are purely administrative, with no consequences for subjects, data or study conduct), the ICF, applicable recruiting materials, and subject compensation programs, and the sponsor has received a copy of this approval. This approval letter must be dated and must clearly identify the IEC/IRB and the documents being approved. During the study the investigator (or sponsor where required) will send the following documents and updates to the IEC/IRB for their review and approval, where appropriate:

- Protocol amendments (excluding the ones that are purely administrative, with no consequences for subjects, data or study conduct)
- Revision(s) to ICF and any other written materials to be provided to subjects
- If applicable, new or revised subject recruiting materials approved by the sponsor
- Revisions to compensation for study-related injuries or payment to subjects for participation in the study, if applicable
- New edition(s) of the Investigator's Brochure and amendments/addenda
- Summaries of the status of the study at intervals stipulated in guidelines of the IEC/IRB (at least annually)
- Reports of adverse events that are serious, unlisted/unexpected, and associated with the study drug
- New information that may adversely affect the safety of the subjects or the conduct of the study
- Deviations from or changes to the protocol to eliminate immediate hazards to the subjects
- Report of deaths of subjects under the investigator's care
- Notification if a new investigator is responsible for the study at the site
- Development Safety Update Report and Line Listings, where applicable
- Any other requirements of the IEC/IRB

For all protocol amendments (excluding the ones that are purely administrative, with no consequences for subjects, data or study conduct), the amendment and applicable ICF revisions must be submitted promptly to the IEC/IRB for review and approval before implementation of the change(s).

At least once a year, the IEC/IRB will be asked to review and reapprove this study. The reapproval should be documented in writing (excluding the ones that are purely administrative, with no consequences for subjects, data, or study conduct).

At the end of the study, the investigator (or sponsor where required) will notify the IEC/IRB about the study completion.

15.2.3. Informed Consent

Each subject must give written consent according to local requirements after the nature of the study has been fully explained. The ICF(s) must be signed before performance of any study related activity. The ICF(s) that is/are used must be approved by both the sponsor and by the reviewing IEC/IRB and be in a language that the subject can read and understand. The informed consent should be in accordance with principles that originated in the Declaration of Helsinki, current ICH and GCP guidelines, applicable regulatory requirements, and sponsor policy.

Before enrollment in the study, the investigator or an authorized member of the study-site personnel must explain to potential subjects the aims, methods, reasonably anticipated benefits, and potential hazards of the study, and any discomfort participation in the study may entail. Subjects will be informed that their participation is voluntary and that they may withdraw consent to participate at any time. They will be informed that if they choose not to participate that this will not affect the care the subject will receive for the treatment of his or her disease. Subjects will be told that alternative treatments are available if they refuse to take part and that such refusal will not prejudice future treatment. Finally, they will be told that the investigator will maintain a subject identification register for the purposes of long-term follow-up if needed and that their records may be accessed by health authorities and authorized sponsor personnel without violating the confidentiality of the subject, to the extent permitted by the applicable law(s) or regulations. By signing the ICF the subject is authorizing such access, including permission to obtain information about his or her survival status, and agrees to allow his or her study physician to recontact the subject for the purpose of obtaining consent for additional safety evaluations,

if needed, and subsequent disease-related treatments, or to obtain information about his or her survival status.

If a subject is unwilling or unable to do a visit post randomization, sites should collect as much information as possible, including contacting the subject or legally acceptable representative by telephone or by mail to determine vital status and if an outcome event has occurred, as agreed to by the subject during the initial informed consent process.

The subject will be given enough time to read the ICF and the opportunity to ask questions. After this explanation and before entry into the study, consent should be appropriately recorded by means of the subject's personally dated signature. After having obtained the consent, a copy of the ICF must be given to the subject.

If the subject is unable to read or write, an impartial witness should be present for the entire informed consent process (which includes reading and explaining all written information) and should personally date and sign the ICF after the oral consent of the subject.

15.2.4. Privacy of Personal Data

The collection and processing of personal data from subjects enrolled in this study will be limited to those data that are necessary to fulfill the objectives of the study.

These data must be collected and processed with adequate precautions to ensure confidentiality and compliance with applicable data privacy protection laws and regulations. Appropriate technical and organizational measures to protect the personal data against unauthorized disclosures or access, accidental or unlawful destruction, or accidental loss or alteration must be put in place. Sponsor personnel whose responsibilities require access to personal data agree to keep the identity of subjects confidential.

The informed consent obtained from the subject includes explicit consent for the processing of personal data and for the investigator/institution to allow direct access to his or her original medical records (source data/documents) for study-related monitoring, audit, IEC/IRB review, and regulatory inspection.

The subject has the right to request through the investigator access to his or her personal data and the right to request rectification of any data that are not correct or complete. Reasonable steps will be taken to respond to such a request, taking into consideration the nature of the request, the conditions of the study, and the applicable laws and regulations.

16. ADMINISTRATIVE REQUIREMENTS

16.1. Protocol Amendments

Neither the investigator nor the sponsor will modify this protocol without a formal amendment by the sponsor. All protocol amendments must be issued by the sponsor and signed and dated by the investigator. Protocol amendments must not be implemented without prior IEC/IRB approval, or when the relevant competent authority has raised any grounds for non-acceptance, except when necessary to eliminate immediate hazards to the subjects, in which case the amendment must be promptly submitted to the IEC/IRB and relevant competent authority. Documentation of amendment approval by the investigator and IEC/IRB must be provided to the sponsor. When the change(s) involves only logistic or administrative aspects of the study, the IRB (and IEC where required) only needs to be notified.

During the study, in situations where a departure from the protocol is unavoidable, the investigator or other physician in attendance will contact the appropriate sponsor representative. Except in emergency situations, this contact should be made before implementing any departure from the protocol. In all cases, contact with the sponsor must be made as soon as possible to discuss the situation and agree on an appropriate course of action. The data recorded in the eCRF and source documents will reflect any departure from the protocol, and the source documents will describe this departure and the circumstances requiring it.

16.2. Regulatory Documentation

16.2.1. Regulatory Approval/Notification

This protocol and any amendment(s) must be submitted to the appropriate regulatory authorities. A study may not be initiated until all local regulatory requirements are met.

16.2. Subject Identification Enrollment and Screening Logs

The investigator agrees to complete a subject identification and enrollment log to permit easy identification of each subject during and after the study. This document will be reviewed by the sponsor study-site contact for completeness.

The subject identification and enrollment log will be treated as confidential and will be filed by the investigator in the study file. To ensure subject confidentiality, no copy will be made. All reports and communications relating to the study will identify subjects by subject identification and date of birth. In cases where the subject is not randomized into the study, the date seen, and date of birth will be used.

The investigator must also complete a subject screening log, which reports on all subjects who were seen to determine eligibility for inclusion in the study.

16.3. Source Documentation

At a minimum, source documentation must be available for the following to confirm data collected in the eCRF: subject identification, eligibility, and study identification; study discussion and date of signed informed consent; dates of visits; results of safety and efficacy parameters as required by the protocol; record of all adverse events and follow-up of adverse events; concomitant medication; drug receipt/dispensing/return records; study drug administration information; and date of study completion and reason for early discontinuation of study drug or withdrawal from the study, if applicable.

In addition, the author of an entry in the source documents should be identifiable.

At a minimum, the type and level of detail of source data available for a subject should be consistent with that commonly recorded at the study site as a basis for standard medical care. Specific details required as source data for the study will be reviewed with the investigator before the study and will be described in the monitoring guidelines (or another equivalent document).

16.4. Case Report Form Completion

Case report forms are provided for each subject in electronic format.

Electronic Data Capture (eDC) will be used for this study. The study data will be transcribed by study-site personnel from the source documents onto an eCRF and transmitted in a secure manner to the sponsor within the timeframe agreed upon between the sponsor and the study site. The electronic file will be the eCRF.

Worksheets may be used for the capture of some data to facilitate completion of the CRF. Any such worksheets will become part of the subject's source documentation. All data relating to the study must be recorded in eCRFs prepared by the sponsor. Data must be entered into eCRFs in English. Study site personnel must complete the eCRF as soon as possible after a subject´s visit, and the forms should be available for review at the next scheduled monitoring visit.

The investigator must verify that all data entries in the eCRFs are accurate and correct.

All eCRF entries, corrections, and alterations must be made by the investigator or other authorized study-site personnel. If necessary, queries will be generated in the eDC tool. The investigator or an authorized member of the study-site personnel must adjust the eCRF and complete the query.

16.5. Data Quality Assurance/Quality Control

Steps to be taken to ensure the accuracy and reliability of data include the selection of qualified investigators and appropriate study sites, review of protocol procedures with the investigator and study-

site personnel before the study, and periodic monitoring visits by the sponsor, and transmission of clinical laboratory data from a central laboratory into the sponsor's data base.

Guidelines for eCRF completion will be provided and reviewed with study-site personnel before the start of the study. The sponsor will review eCRFs for accuracy and completeness during on-site monitoring visits and after their return to the sponsor; any discrepancies will be resolved with the investigator or designee, as appropriate. The data will be entered into the study database and verified for accuracy and consistency with the data sources.

16.6. Record Retention

In compliance with the ICH/GCP guidelines, the investigator/institution will maintain all case report forms and all source documents that support the data collected from each subject, as well as all study documents as specified in ICH/GCP Section 8, Essential Documents for the Conduct of a Clinical Trial, and all study documents as specified by the applicable regulatory requirement(s). The investigator/institution will take measures to prevent accidental or premature destruction of these documents.

Essential documents must be retained until at least 2 years after the last approval of a marketing application in an ICH region and until there are no pending or contemplated marketing applications in an ICH region or until at least 2 years have elapsed since the formal discontinuation of clinical development of the investigational product. These documents will be retained for a longer period if required by the applicable regulatory requirements or by an agreement with the sponsor.

If the responsible investigator retires, relocates, or for other reasons withdraws from the responsibility of keeping the study records, custody must be transferred to a person who will accept the responsibility. The sponsor must be notified in writing of the name and address of the new custodian. Under no circumstance shall the investigator relocate or dispose of any study documents before having obtained written approval from the sponsor.

If it becomes necessary for the sponsor or the appropriate regulatory authority to review any documentation relating to this study, the investigator/institution must permit access to such reports.

16.7. Monitoring

The sponsor will perform on-site monitoring visits as frequently as necessary. The monitor will record dates of the visits in a study site visit log that will be kept at the study site. At these visits, the monitor will compare a sample of the data entered into the eCRFs with the hospital or clinic records (source documents). The nature and location of all source documents will be identified to ensure that all sources of original data required to complete the eCRF are known to the sponsor and study-site personnel and are accessible for verification by the sponsor study-site contact. If electronic records are maintained at the study site, the method of verification must be discussed with the study-site personnel.

Direct access to source documentation (medical records) must be allowed for the purpose of verifying that the data recorded in the eCRF are consistent with the original source data. Findings from this review of eCRFs and source documents will be discussed with the study-site personnel. The sponsor expects that, during monitoring visits, the relevant study-site personnel will be available, the source documentation will be accessible, and a suitable environment will be provided for review of study-related documents. The monitor will meet with the investigator on a regular basis during the study to provide feedback on the study conduct.

In addition to on-site monitoring visits, remote contacts can occur. It is expected that during these remote contacts, study-site personnel will be available to provide an update on the progress of the study at the site.

16.8. Study Completion/Termination

16.8.1. Study Completion

The study is considered completed with the last study assessment for the last subject participating in the study. The final data from the study site will be sent to the sponsor (or designee) after completion of the final subject assessment at that study site, in the time frame specified in the Clinical Trial Agreement.

16.8.2. Study Termination

The study will be discontinued if the sponsor or its designee judges it necessary for medical, safety, regulatory, or other reasons consistent with applicable laws, regulations, and GCP guidelines.
The following events, if applicable, may cause premature termination of the clinical study:

- Early evidence of overt inferiority of the treatment according to the recommendation of the DSMB (decision taken by the sponsor);
- Unjustifiable risk and/or toxicity in risk-benefit analysis (decision taken by the sponsor), e.g. when adverse events occur, unknown to date with respect to their nature, severity, duration or frequency in relation to the currently established safety profile (substantial changes to the risk-benefit ratio), and therefore medical and/or ethical reasons affect the continuation of the study;
- New scientific evidence provided during the study that could affect the patient's safety (benefit-risk analysis no longer positive);
- Request of the sponsor or regulatory agency.

In case the risk profile of the study worsens and a modification of the maximum insurance coverage is not possible.

16.9. On-Site Audits

Representatives of the sponsor's clinical quality assurance department may visit the study site at any time during or after completion of the study to conduct an audit of the study in compliance with regulatory guidelines and company policy. These audits will require access to all study records, including source documents, for inspection and comparison with the case report forms. Subject privacy must, however, be respected. The investigator and study-site personnel are responsible for being present and available for consultation during routinely scheduled study-site audit visits conducted by the sponsor or its designees.

Similar auditing procedures may also be conducted by agents of any regulatory body, either as part of a national GCP compliance program or to review the results of this study in support of a regulatory submission. The investigator should immediately notify the sponsor if he or she has been contacted by a regulatory agency concerning an upcoming inspection.

16.10. Use of Information and Publication

The results of the study will be reported in a Clinical Study Report generated by the sponsor and will contain eCRF data from all study sites that participated in the study, and transmission of clinical laboratory data from a central laboratory into the sponsor's database. Results of exploratory analyses performed after the Clinical Study Report has been issued will be reported in a separate report and will not require a revision of the Clinical Study Report.

Authorship of publications resulting from this study will be based on the guidelines on authorship, such as those described in the Uniform Requirements for Manuscripts Submitted to Biomedical Journals, which state that the named authors must have made a significant contribution to the design of the study or analysis and interpretation of the data, provided critical review of the paper, and given final approval of the final version.

Registration of Clinical Studies and Disclosure of Results

The sponsor will register and/or disclose the existence of and the results of clinical studies as required by law.

17. SUBSTUDIES

There is one prespecified substudy:

17.1. Bio-banking (optional)

Participation in sampling for bio-banking is voluntary and not a pre-requisite for participation in the trial. Bio-banking samples will be taken only after a separate informed consent has been given in accordance with local ethical and regulatory requirements. Banked samples may be analyzed in the future for scientific evaluations or to further, for example, the mechanistic understanding of drug effects and/or identify genetic or other factors associated with response therapy or the risk of adverse drug reactions.

18. Protocol amendments

18.1. Amendment 1

Amendment 1 is dated 20 AUG 2021.

18.1.1 Overview of changes

Amendment 1: 20 AUG 2021

This amendment is considered to be substantial based on the criteria set forth in Article 10(a) of Directive 2001/20/EC of the European Parliament and the Council of the European Union.

Overall Rationale for the Amendment

The initial planned sample size based on statistical calculations was 400 patients. Due to decreasing COVID incidence rates, it became very likely not to be able to recruit the planned patient number within a reasonable time frame to contribute with the results to the treatment of patients suffering from COVID.

Section # and Name	Description of Change	Brief Rationale
**4.2 Exclusion Criteria**<br><br>**and**<br><br>**4.3 Prohibitions and restrictions**<br><br>**and**<br><br>**8. Prestudy and concomitant therapy**	**4.2 Exclusion Criteria**<br>2 b: Any medical condition (e.g. atrial fibrillation) that requires use of any therapeutic parenteral or oral anticoagulant(s) (e.g. warfarin sodium or other vitamin K antagonists, Factor IIa or FXa inhibitors, fibrinolytics) concomitantly with study medication. In case of “off label” use of a NOAC, the patient can be included in the study if the NOAC was stopped for 24 hours or more.<br>**4.3 Prohibitions and restrictions**<br>Anticoagulation with warfarin sodium or other vitamin K antagonists is prohibited during the study. Factor IIa inhibitors or other FXa inhibitors than rivaroxaban must not be used as concomitant therapy during the study. If a patient doesn’t have an indication for anticoagulation but received a NOAC with an “off label” use, the NOAC has to be stopped 24 hours prior to randomization.<br>**8. Prestudy and concomitant therapy**<br>The following concomitant medications are not allowed while enrolled in the	In the course of the study, it became obvious that some centers started the treatment with a NOAC for thromboprophylaxis in COVID patients as off-label use. Therefore it was added how such a patient should be handled to ensure the patient safety.

	study. Please also refer to the SmPC of the respective study drug.<br>• ….<br>• apixaban within 24 hours prior to randomization<br>• dabigatranetexilat within 24 hours prior to randomization<br>• edoxaban within 24 hours prior to randomization<br>• ……	
**9.2.1. Efficacy Evaluations, Outcomes and Adjudication**<br>*Primary endpoints*	~~This is a clinical outcomes study.~~ The primary ~~efficacy~~ endpoint is ~~are~~ the following:<br>• D-dimer at day 7 post randomization<br>The co-primary endpoint is the following:<br>• Seven-category ordinal scale recommended by the WHO at day 7 post randomization (Attachment 2)<br>• ~~VTE (DVT and/or fatal or non-fatal PE)~~<br>• ~~Arterial thromboembolism~~<br>• ~~New MI~~<br>• ~~Non-hemorrhagic stroke~~<br>• ~~All-cause death~~<br>• ~~Progression to intubation and invasive ventilation~~	The primary endpoint was changed to account for the lower patient number recruited to allow a meaningful analysis. The biomarker D-dimer was chosen as surrogat marker for the clinical prognosis of COVID patients.<br>As co-primary endpoint the WHO recommended „seven-category ordinal scale“ was chosen as objective parameter for the clinical course of COVID-patients.<br>Given the lower overall patient number an objectification of the clinical course based on the WHO recommended scale seems to be more sensitive than only documenting the randomly occurring clinical endpoints. Moreover, it is common practice for studies on COVID but also on pneumonia to use this scale as an endpoint to judge on the clinical course.
**9.2.1. Efficacy Evaluations, Outcomes and Adjudication**<br>*Secondary endpoints*	The secondary combined efficacy endpoint~~s~~ is ~~are~~ the following:<br>• Venous thromboembolism (VTE) (deep venous thrombosis (DVT) and/or fatal or non-fatal pulmonary embolism (PE))<br>• Arterial thromboembolism	Except the „seven-category ordinal scale“ all other secondary endpoints were ventilation related and on development of DIC. Given that we were not allowed to continue with the patient observation even in case of

	• New myocardial infarction (MI)<br>• Non-hemorrhagic stroke<br>• All-cause death<br>• Progression to intubation and invasive ventilation<br><br>• ~~Development of DIC~~<br>• ~~Seven-category ordinal scale recommended by the WHO as clinical improvement scale for patients with respiratory infections (23-25)~~<br>• ~~Number of days requiring invasive ventilation~~<br>• ~~Number of days requiring non-invasive ventilation~~	loss of ability to consent only if the patient agreed to be re-consented. Therefore many data needed for these secondary endpoints couldn´t be adequately documented.
**9.2.1. Efficacy Evaluations, Outcomes and Adjudication**<br><br>*Other endpoints of interest*	**Other endpoints of interest:**<br>• Length of hospital stay<br>• Time to intubation<br>• Re-hospitalization due to heart failure<br>• Re-hospitalization due to any other reason<br>• ~~Proportion of patients requiring catecholamines, time to catecholamines and time on catecholamines~~<br>• Effects on coagulation parameters for thrombosis (TAT, PAI-1, PF-4, TF, TF-activity)<br>• Effects on inflammatory and fibrotic ~~coagulation~~ parameters ~~for thrombin formation~~ (~~D- dimer~~ interleukines, interferons, growth factors)<br>• Change of N-terminal prohormone brain natriuretic peptide (NT-pro-BNP)<br>• Unscheduled outpatient visits	The endpoint “proportion of patients requiring catecholamines” was deleted due to the fact that patients during their time of ICU were not allowed to be documented only in case of a re-consenting after regaining the ability to give consent. Therefore, the information on catecholamine application couldn´t be captured adequately.<br><br>The measurement of markers for thrombosis, inflammation, fibrosis and growth was added as “other endpoints on interest” for those patients who gave the consent for this substudy. The aim is to characterize both treatment groups in regard of the different markers and to understand the course of the disease under the different treatment conditions.
**11.7 sample size determination**	Sample size calculation is based on the primary endpoint D-dimer at day 7	As mentioned above, the endpoints were changed which also lead to an

	corrected for baseline values. Since the distribution of D-dimer concentrations is skewed, calculations are done on the log scale. Using an analysis of covariance (ANCOVA) (significance level alpha=0.05 two-sided) with baseline values as covariate a total sample size of 80, i.e. 40 per group, gives a power of 80% to detect a mean difference of 0.44 (on the log scale) between the treatment group and the control group at day 7. Based on a blinded review, a common standard deviation of SD=0.8 and a correlation to baseline of r=0.5 (both on the natural log scale) were assumed. The effect assumed translates to a reduction of 36% in D-Dimer values on the original scale. The co-primary endpoint is a seven-category ordinal scale previously recommended by the WHO (Attachment 2) as clinical improvement scale for patients with respiratory infections. Here 40 patients per group will give a power of 80% to detect a relative treatment effect p1=0.68 between treatment and control group at day 7 using a Wilcoxon (Mann-Whitney) rank- sum test (two sided, alpha=0.05). The probabilistic index or relative effect p1 denotes the probability that an observation in the treatment group will be less than an observation in the control group. Targeting a total of at least 80 evaluable patients, we aim to recruit about 100 patients.<br><br>~~A total of 120 primary endpoint events yields a power of 80% at a two-sided significance level of 5% given a hazard ratio of 0.60, i.e. a reduction of 40% in terms of hazards for the primary endpoint, and 1:1 randomization (Schoenfeld formula). This event-driven study is completed once 120 events are observed. The sample size per group is then given depending on the proportion of patients in the control group having at least one primary endpoint event.~~	adaptation of the sample size. Based on the new primary endpoint D-dimer on day 7 a new sample size was calculated. The aim is now to close the study with approximately 100 patients. This sample size is also sufficient for the new „co-primary“ endpoint „seven-category ordinal scale“.<br>111 patients have been randomized into the study until the trial has been set on-hold on June 11, 2021. After the proposed amendment has been evaluated by the regulatory authorities, it is planned to stop the study without recruiting additional patients into the study.

**11.2 Primary and co-primary efficacy endpoints**<br><br>**and**<br><br>**11.3 Secondary Efficacy endpoints**	**11.2**<br>The primary efficacy endpoint is D-dimer at day 7 post randomization will adjusted for baseline measurement. The logarithmic D-dimer measurements (using the natural logarithm) will be analyzed by an analysis of covariance (ANCOVA). The model will include treatment group and stratification variables of the randomization as factors and the logarithmic baseline D-dimer measurement as covariate. Least squares means for D-dimer at day 7 in the two treatment groups will be presented with 95% confidence intervals as well as the difference between the treatment groups at day 7 with 95% confidence interval and p-value testing the null hypothesis of no treatment difference, i.e. a mean difference (on the logarithmic scale) of 0. For ease of interpretation the results will also be converted to the original measurement scale.<br><br>The co-primary endpoint is the seven-category ordinal scale recommended by the WHO (Attachment 2) at day 7 post randomization adjusted for baseline score. Frequencies and percentages of the scores at day 7 will be provided stratified by treatment group. The distributions of the scores will be compared between the two treatment groups using a nonparametric Wilcoxon (Mann-Whitney) rank-sum test stratified by baseline score dichotomized as smaller or equal 3 vs. larger 3. The treatment effect will be reported as probabilistic index or relative effect, i.e. the probability that an observation in the treatment group will be smaller (more favourable) than an observation in the control group, with 95% confidence interval.<br><br>~~will be analyzed using a Cox proportional hazards regression model with treatment and the stratification variables of the randomization (site, gender (male, female, diverse), age (<~~	For the new endpoints the respective statistical tests for their analyses were added. For the other endpoints the initially planned analyses were kept. Nevertheless, there were changes in the presentation as for example the explanation for the analysis of the combined endpoint was moved to the secondary endpoints.

	~~65 versus > 65 years), kidney function (subjects with eGFR ≥ 30 mL/min/1.73m^2 and < 50 mL/min/1.73m^2 versus subjects with an eGFR > 50 mL/min/1.73m^2), history of CAD or heart failure (yes/no), oxygen demand on admission to the hospital (supplementary oxygen required versus not required), setting (outpatients versus hospitalized patients)) as factors. The treatment effect will be reported as hazard ratio (HR) with 95% confidence interval and p-value for the null hypothesis H0: HR=1. The primary endpoint will be visualized as Kaplan-Meier curves. Sensitivity analyses will explore the robustness of these analyses. These include models adjusting for important prognostic factors including, e.g. smoking status, diabetes, presence of coronary heart disease or heart failure.~~<br><br>**<u>11.3</u>**<br><br><u>Time to first event of either of the components of the secondary composite endpoint will be analyzed using a Cox proportional hazards regression model with treatment and the stratification variables of the randomization (site, gender (male, female, diverse), age (< 65 versus ≥ 65 years), kidney function (eGFR ≥ 30 mL/min/1.73m^2 and < 50 mL/min/1.73m^2 versus eGFR ≥ 50 mL/min/1.73m^2), history of CAD or heart failure (yes/no), oxygen demand on admission to the hospital (supplementary oxygen required versus not required), setting (outpatients versus hospitalized patients)) as factors. The treatment effect will be reported as hazard ratio (HR) with 95% confidence interval and p-value for the null hypothesis H0: HR=1. The primary endpoint will be visualized as Kaplan-Meier curves. Sensitivity analyses will explore the robustness of these analyses.</u>	

	Longitudinal D-dimer measurements assessed at day 7, 35 and 60 will be analyzed on the logarithmic scale using a mixed model for repeated measures (MMRM) approach. The model will include treatment, visit, treatment-by-visit interaction and stratification variables of the randomization as factors as well as logarithmic D-dimer baseline value as covariate. The residuals will be assumed to follow a multivariate normal distribution with unstructured covariance matrix. LS means of the group means and their differences will computed for the various timepoints and reported with 95% confidence intervals.<br><br>Number of days requiring invasive/non-invasive ventilation will be analyzed using a multistate model (28) with the following states: not requiring ventilation, requiring non-invasive ventilation, requiring invasive ventilation and death. The models will be adjusted for the stratification variables of the randomization.	

19. REFERENCES

1. GmbH RLS. <Xarelto 10mg Fachinfo.pdf>. 2019.
2. GmbH RLS. <Xarelto 2,5mg Fachinfo.pdf>. 2019.
3. GmbH RLS. <Xarelto 20mg Fachinfo.pdf>. 2019.
4. Perlman S. Another Decade, Another Coronavirus. N Engl J Med. 2020;382(8):760-2.
5. Danzi GB, Loffi M, Galeazzi G, Gherbesi E. Acute pulmonary embolism and COVID-19 pneumonia: a random association? Eur Heart J. 2020.
6. Guan WJ, Ni ZY, Hu Y, Liang WH, Ou CQ, He JX, et al. Clinical Characteristics of Coronavirus Disease 2019 in China. N Engl J Med. 2020.
7. Thachil J, Tang N, Gando S, Falanga A, Cattaneo M, Levi M, et al. ISTH interim guidance on recognition and management of coagulopathy in COVID-19. J Thromb Haemost. 2020.
8. Huang C, Wang Y, Li X, Ren L, Zhao J, Hu Y, et al. Clinical features of patients infected with 2019 novel coronavirus in Wuhan, China. The Lancet. 2020;395(10223):497-506.
9. Tang N, Li D, Wang X, Sun Z. Abnormal coagulation parameters are associated with poor prognosis in patients with novel coronavirus pneumonia. J Thromb Haemost. 2020;18(4):844-7.
10. Han H, Yang L, Liu R, Liu F, Wu KL, Li J, et al. Prominent changes in blood coagulation of patients with SARS-CoV-2 infection. Clin Chem Lab Med. 2020.
11. Zhou F, Yu T, Du R, Fan G, Liu Y, Liu Z, et al. Clinical course and risk factors for mortality of adult inpatients with COVID-19 in Wuhan, China: a retrospective cohort study. The Lancet. 2020;395(10229):1054-62.
12. Klok FA, Kruip M, van der Meer NJM, Arbous MS, Gommers D, Kant KM, et al. Incidence of thrombotic complications in critically ill ICU patients with COVID-19. Thromb Res. 2020.
13. Cui S, Chen S, Li X, Liu S, Wang F. Prevalence of venous thromboembolism in patients with severe novel coronavirus pneumonia. J Thromb Haemost. 2020.
14. Llitjos JF, Leclerc M, Chochois C, Monsallier JM, Ramakers M, Auvray M, et al. High incidence of venous thromboembolic events in anticoagulated severe COVID-19 patients. J Thromb Haemost. 2020.
15. Luo W, Yu H, Gou J, Li X, Sun Y, Li X, et al. <Clinical pathology of critical patient with novel coronavirus pneumonia (COVID-19).pdf>. 2020.
16. Tang N, Bai H, Chen X, Gong J, Li D, Sun Z. Anticoagulant treatment is associated with decreased mortality in severe coronavirus disease 2019 patients with coagulopathy. J Thromb Haemost. 2020.
17. Driggin E, Madhavan MV, Bikdeli B, Chuich T, Laracy J, Bondi-Zoccai G, et al. Cardiovascular Considerations for Patients, Health Care Workers, and Health Systems During the Coronavirus Disease 2019 (COVID-19) Pandemic. J Am Coll Cardiol. 2020.
18. Ruan Q, Yang K, Wang W, Jiang L, Song J. Clinical predictors of mortality due to COVID-19 based on an analysis of data of 150 patients from Wuhan, China. Intensive Care Med. 2020.
19. Lippi G, Lavie CJ, Sanchis-Gomar F. Cardiac troponin I in patients with coronavirus disease 2019 (COVID-19): Evidence from a meta-analysis. Prog Cardiovasc Dis. 2020.
20. Bansal M. Cardiovascular disease and COVID-19. Diabetes Metab Syndr. 2020;14(3):247-50.
21. Cohen AT, Spiro TE, Buller HR, Haskell L, Hu D, Hull R, et al. Rivaroxaban for thromboprophylaxis in acutely ill medical patients. N Engl J Med. 2013;368(6):513-23.

22. Spyropoulos AC, Ageno W, Albers GW, Elliott CG, Halperin JL, Hiatt WR, et al. Rivaroxaban for Thromboprophylaxis after Hospitalization for Medical Illness. N Engl J Med. 2018;379(12):1118-27.
23. Cao B, Wang C, Guo L, Lu B, Zheng J, Pan J, et al. Comparative Effectiveness of Combined Favipiravir and Oseltamivir Therapy Versus Oseltamivir Monotherapy in Critically Ill Patients With Influenza Virus Infection. The Journal of Infectious Diseases. 2020;221(10):1688-98.
24. Cao B, Wang Y, Wen D, Liu W, Wang J, Fan G, et al. A Trial of Lopinavir-Ritonavir in Adults Hospitalized with Severe Covid-19. N Engl J Med. 2020;382(19):1787-99.
25. WHO. Coronavirus disease (COVID-2019) R&D. Geneva 2019 [Available from: https://www.who.int/teams/blueprint/covid-19
26. Bikdeli B, Madhavan MV, Jimenez D, Chuich T, Dreyfus I, Driggin E, et al. COVID-19 and Thrombotic or Thromboembolic Disease: Implications for Prevention, Antithrombotic Therapy, and Follow-up. J Am Coll Cardiol. 2020.
27. Mehran R, Rao SV, Bhatt DL, Gibson CM, Caixeta A, Eikelboom J, et al. Standardized bleeding definitions for cardiovascular clinical trials: a consensus report from the Bleeding Academic Research Consortium. Circulation. 2011;123(23):2736-47.
28. Meira-Machado L, de Uña-Álvarez J, Cadarso-Suárez C, Andersen PK. Multi-state models for the analysis of time-to-event data. Statistical Methods in Medical Research. 2009;18(2):195-222.

Attachment 1: Lower-dose prophylactic anticoagulation

For prophylactic anticoagulation, the following doses should not be exceeded:

UFH:

- 5.000 - I.U. every 8 or 12 hours or
- .7500 I.U. every 12 hours
- Maximal cumulative dose of 15.000 I.U. when infused continuously i.v. over 24 hours

LMWH:

Enoxaparin

- 40 mg every 24 hours or 30mg every 12 hours s.c. or
- 40 mg every 12 hours s.c. for weight >150kg or BMI >40-50 or
- 60 mg every 12 hours s.c. for BMI >50

Dalteparin

- 5.000 I.U. every 24 hours s.c.

Nadroparin

- for patients ≤ 70kg 3.800 I.U. every 24 hours s.c.
- for patients > 70kg 5.700 I.U. every 24 hours s.c.

Tinzaparin

- 3.500 I.U. every 24 hours s.c.

Certoparin

- 3.000 I.U. every 24 hours s.c.

Reviparin

- 1.750 I.U. every 24 hours s.c.

Attachment 2: Seven category ordinal scale recommended by the WHO (23-25)

7: Death
6: ICU, requiring invasive mechanical ventilation
5: ICU, not requiring invasive mechanical ventilation
4: Non-ICU, requiring oxygen
3: Non-ICU, not requiring oxygen
2: Discharged without resumption of normal activities
1: Discharged with resumption of normal activities

Attachment 3: CKD-EPI Formula

eGFR = 141 * min $(Scr/\kappa,1)^{\alpha}$ * max$(Scr/\kappa, 1)^{-1.209}$ * 0.993^{Age} * 1.018 [if female] * 1.159 [if black]

- Scr is serum creatinine (mg/dL)
- κ is 0.7 for females and 0.9 for males
- α is -0.329 for females and -0.411 for males
- min indicates the minimum of Scr/κ or 1
- max indicates the maximum of Scr/κ or 1

Attachment 4: Secondary efficacy endpoints and other endpoints of interest

All efficacy evaluations and endpoints described in Section 9.2 will be evaluated and adjudicated by the CEC (defined below). Safety endpoints are defined in Section 9.4.

Secondary efficacy endpoints

1. DVT – displaying signs or symptoms of proximal or distal lower extremity DVT, upper extremity DVT, or other DVT and confirmed by adjudication, based on 1 or more of the following diagnostic criteria:
    1. a non-compressible venous segment on compression ultrasonography, or in patients with a history of previous DVT, either a new non-compressible venous segment or a substantial increase (4 mm or more) in the diameter of the vein during full compression in a previously abnormal segment on ultrasonography, or
    2. the presence of an intraluminal filling defect on venography, or
    3. DVT documented at autopsy.

    If in patients with history of previous DVT or incomplete documentation of the previous episode is available, additional criteria may be integrated into the adjudication of the current event, such as: ultrasonography appearance of the thrombus. The totality of available clinical, imaging and laboratory findings should be considered.

2. PE – displaying signs or symptoms suggestive of PE and confirmed by adjudication based on 1 or more of the following diagnostic criteria:
    1. an intraluminal filling defect on CT angiography or spiral CT, or
    2. an intraluminal filling defect on pulmonary angiography or cutoff of a vessel more than 2.5 mm in diameter, or
    3. a perfusion lung scan defect of at least 75% of a segment with corresponding normal ventilation (high probability ventilation-perfusion (V-Q) scan), or
    4. a non-high probability V-Q scan abnormality associated with DVT documented by ultrasonography or venography, or
    5. in the absence of imaging test in a hemodynamically unstable patient, evidence of right ventricular dysfunction by transthoracic or transesophageal echocardiogram (ESC criteria), or
    6. PE documented at autopsy.

    The anatomic extent of PE will be classified by the adjudication committee as either segmental or greater, or sub-segmental. The totality of clinical, imaging and laboratory findings should be considered.

3. New myocardial infarction: New Type 1 MI and Type 2 MI. Requires combination of evidence of myocardial necrosis (either changes in cardiac biomarkers or post-mortem pathological findings), and supporting information from clinical presentation, ECG changes or the results of myocardial imaging or coronary artery imaging. The totality of available clinical, ECG and cardiac biomarker should be considered.

4. Ischemic stroke – acute episode of focal cerebral, spinal, or retinal dysfunction caused by infarction of central nervous system tissue.

5. Death will be assigned to 1 of the following:
    1. CV death
        - i) VTE-related: VTE-related death – death due to confirmed PE documented by objective testing or autopsy
        - ii) VTE-related, PE cannot be ruled out – death, which cannot be attributed to a documented cause and for which pulmonary embolism cannot be ruled out (unexplained death)
        - iii) Other CV death
    2. Non-CV death – death not included in 1 of the above categories

6. Progression to intubation and invasive ventilation defined as the use of any instrument penetrating via the mouth (such as an endotracheal tube), nose, or the skin (such as a tracheostomy tube) to serve as an artificial airway.

Other endpoints of interest

Re-hospitalization for heart failure:

A heart failure event for patients admitted to a hospital and treated as an inpatient should be defined by meeting at least one criterion from each subheading below (biomarker evidence, radiographic or hemodynamic evidence, heart failure symptoms, and intensification of treatment) and/or at least one for heart failure signs.
Biomarker evidence: At least one of the following:

- Brain natriuretic peptide (BNP; e.g. ≥100 ng/L)
- N-terminal pro-BNP (e.g. ≥300 ng/L)

Radiographic or hemodynamic evidence: At least one of the following:

- Non-invasive diagnostic evidence of heart failure (e.g. echocardiographic, cardiac MRI)
- Radiographic evidence of pulmonary congestion
- Invasive catheterization with evidence of heart failure (e.g., pulmonary capillary wedge pressure [or LVEDP] >18 mm Hg, right arterial pressure [or central venous pressure] >12 mm Hg, or CI < 2.2 L/min per m^2)

Heart failure symptoms: At least one of the following:

- Dyspnea
- Decreased exercise tolerance
- Fatigue
- Worsened end-organ perfusion
- Other symptoms of volume overload (e.g. swelling of legs, etc.)

Heart failure signs: At least one of the following:

- Peripheral edema
- Increasing abdominal distension or ascites
- Pulmonary rales or crackles, or crepitation
- Increased jugular venous pressure, hepatojugular reflux, or both
- Third heart sound or gallop
- Clinically significant rapid weight gain related to fluid accumulation)

Intensification of treatment: At least one of the following:

- Augmentation of oral diuretic therapy
- Intravenous diuretic or intravenous vasoactive therapy

Attachment 5: Clinical Classification of Severity of COVID-19

We use the modified classification released by National Health Commission & State Administration of Traditional Chinese Medicine on March 3, 2020 [27].

1. Moderate cases: Showing fever and/or respiratory symptoms and clinical signs of pulmonary distress, such as:

    a. Respiratory rate >22/min;
    b. Reduced oxygen saturation ≤95 % at rest;
    c. Radiological findings of pneumonia.

2. Severe cases: Cases meeting any of the following criteria:
    1. Respiratory distress (≧30 breaths/ min);
    2. Oxygen saturation ≤93% at rest;
    3. Arterial partial pressure of oxygen (PaO_2)/ fraction of inspired oxygen (FiO_2)≦ 300mmHg (l mmHg=0.133kPa). In high-altitude areas (at an altitude of over 1,000 meters above the sea level), PaO_2/ FiO_2 shall be corrected by the following formula: PaO_2/ FiO_2 x [Atmospheric pressure (mmHg)/760]

    Cases with chest imaging that showed obvious lesion progression within 24-48 hours >50% shall be referred to as severe cases.
